# Supplementary material for: A new paravian dinosaur from the Late Jurassic of North America supports a late acquisition of avian flight
Source: PeerJ. 2019 Jul 10;7:e7247. doi: 10.7717/peerj.7247 (PMC6626525; doi:10.7717/peerj.7247)
Supplement: Supplemental Information 1 — Includes a historical chart of Theropod Working Group-based phylogenetic analyses (S1) and a complete strict consensus tree resulting from our phylogenetic analysis (S2). The complete revised character list and taxonomic considerations, including OTU selection and exclusion, as well as supplemental citations used in character scoring. [file peerj-07-7247-s001.doc]

**Supplemental phylogenetic data for: A new paravian dinosaur from the Late Jurassic of North America sheds light on avialan phylogeny and supports a late acquisition of avian flight**

Scott A. Hartman^1^, Mickey Mortimer^2^, William R. Wahl^3^, Dean R. Lomax^4^,

Jessica Lippincott^3^, David M. Lovelace^5^

^1^Department of Geoscience, University of Wisconsin-Madison, 1215 W. Dayton Street, Madison, WI, USA, 53706

^2^27988 Maple Ridge Way SE, Maple Valley, WA, USA 98038

^3^Wyoming Dinosaur Center, 110 Carter Ranch Road, Thermopolis, WY, USA, 82443

^4^The University of Manchester, School of Earth and Environmental Sciences, Oxford Road, Manchester, UK, M13 9PL

^5^UW Geology Museum, University of Wisconsin-Madison, 1215 W. Dayton Street, Madison, WI, USA, 53706

Corresponding Author:

Scott A. Hartman^1^

Email address: sahartman@wisc.edu

**Norell et al., 2001 (Ostrom volume)**

**Xu, 2002 (thesis)**

**Xu and Wang, 2004b (*Graciliraptor*)**

**Xu and Zhang, 2005 (*Pedopenna*)**

**Xu et al., 2002a (*Sinovenator*)**

**Wahl, 2006 (thesis)**

**Clark et al., 2002 (Mesozoic Birds)**

**Hwang et al., 2002 (*Microraptor*)**

**Mayr et al., 2005 (*Archaeopteryx*)**

**Xu et al., 2002c (*Incisivosaurus*)**

**Ji et al., 2003 (*Shenzhousaurus*)**

**Makovicky et al., 2003 (*Byronosaurus*)**

**Calvo et al., 2004 (*Unenlagia paynemili*)**

**Hwang et al., 2004 (*Huaxiagnathus*)**

**Gohlich and Chiappe, 2006 (*Juravenator*)**

**Kobayashi, 2004 (thesis)**

**Lu, 2004 (thesis)**

**Makovicky et al.; Makovicky and Norell; Norell and Makovicky, 2004 (Dinosauria 2)**

**Xu and Norell, 2004 (*Mei*)**

**Novas and Pol, 2005 (*Neuquenraptor*)**

**Makovicky et al., 2005 (*Buitreraptor*)**

**Norell et al., 2006 (*Tsaagan*)**

**Turner et al., 2007a (*Shanag*)**

**Xu et al., 2007 (*Gigantoraptor*)**

**Turner et al., 2007b (*Mahakala*)**

**Turner 2008 (thesis)**

**Turner et al., 2012 (Dromaeosauridae)**

**Evans et al., 2013 (*Acheroraptor*)**

**Han et al., 2014 (*Changyuraptor*)**

**Lu and Brusatte, 2015 (*Zhenyuanlong*)**

Brusatte, 2013 (thesis)

Brusatte et al., 2014 (coeluro) (102)

Cau et al., 2015 (*Balaur*) (4)

Cau et al., 2017 (*Halszkaraptor*) (0)

Pei, 2015 (thesis) (9)

Brusatte et al., 2016 (*Timurlengia*) (0)

Shen et al., 2017 (*Daliansaurus*) (0)

Gianechini et al., 2018 (*Buitreraptor* postcrania) (6)

Yu et al., 2018 (*Anomalipes*) (4)

**Sues and Averianov, 2014 (*Itemirus*)**

**Balanoff et al., 2015 (*Khaan* coelur brain)**

**Azuma et al., 2016 (*Fukuivenator*)**

Gianechini et al., 2017 (*Buitreraptor* skull) (3)

**Novas et al., 2009 (*Austroraptor*)**

**Li et al., 2010, Makovicky et al., 2010 (*Beishanlong*, *Xiongguanlong*)**

**Zanno and Makovicky, 2011 (EC Tyrann)**

**Makovicky et al., 2012 (*Alnashetri*)**

**Serrano-Branas et al., 2015 (*Tototlmimus*)**

**Csiki et al., 2010 (*Balaur*)**

**Nesbitt et al., 2011 (*Albinykus*)**

**Zanno et al., 2009 (*Nothronychus graffami*)**

**Zanno, 2010b (Therizinosauria)**

**Dececchi et al., 2012 (*Yixianosaurus*)**

**Pu et al., 2013 (*Jianchangosaurus*)**

**Sues and Averianov, 2015 (Bissekty theriz)**

**Xu et al., 2013 (*Yixianosaurus*)**

**Prieto-Marquez et al., 2012 (Oosh deinonychosaur)**

**Hwang, 2007 (thesis)**

**Novas et al., 2008 (*Orkoraptor*)**

**Xu et al., 2009 (*Anchiornis*)**

**Martinelli and Vera, 2007 (*Achillesaurus*)**

**Kirkland et al., 2005 (*Falcarius*)**

**Senter, 2007 (coelurosaurs)**

**Zhang et al., 2008 (*Epidexipteryx*)**

**Hu et al., 2009 (*Anchiornis*)**

**Agnolin and Novas, 2011 (Unenlagiidae)**

Agnolin and Novas, 2013 (Paraves) (2)

**Jiang, 2011 (thesis)**

**Xu et al., 2011 (*Xiaotingia*)**

**O'Connor and Sullivan, 2014 (*Zhongornis*)**

Foth et al., 2014 (*Archaeopteryx*) (1)

Foth and Rauhut, 2017 (*Ostromia*) (1)

**Xu et al., 2015 (*Yi*)**

**Xu et al., 2017 (*Jianianhualong*)**

**Hu et al., 2018 (*Caihong*)**

**Xu et al., 2011 (*Linhevenator*)**

**Xu et al., 2012 (*Philovenator*)**

**Gao et al., 2012 (*Mei*)**

**Tsuihiji et al., 2014 (*Gobivenator*)**

**Averianov and Sues, 2016 (*Urbacodon*)**

van der Reest and Currie, 2017 (*Latenivenatrix*) (2)

**Tsuihiji et al., 2015 (IGM 100/140)**

**Shen et al., 2017 (*Liaoningvenator*)**

**Zheng et al., 2010 (*Tianyuraptor*)**

**Novas et al., 2012 (*Bicentenaria*)**

**DePalma et al., 2015 (*Dakotaraptor*) (0)**

**Senter, 2010 (creation)**

**Senter et al., 2010 (*Geminiraptor*)**

Senter, 2011 (creation 2) (20)

Senter et al., 2012a (*Yurgovuchia*) (0)

Senter et al., 2012b (*Martharaptor*) (0)

**Dal Sasso and Maganuco, 2011 (*Scipionyx*)**

**Figure S1**. Geneology of Theropod Working Group analyses, with indented references being expanded from the preceding reference. All Mesozoic maniraptoromorphs from these analyses have been used here, and each **bolded** reference has had its character list completely utilized. Number of characters informative within Maniraptoromorpha yet to be analyzed in parentheses.


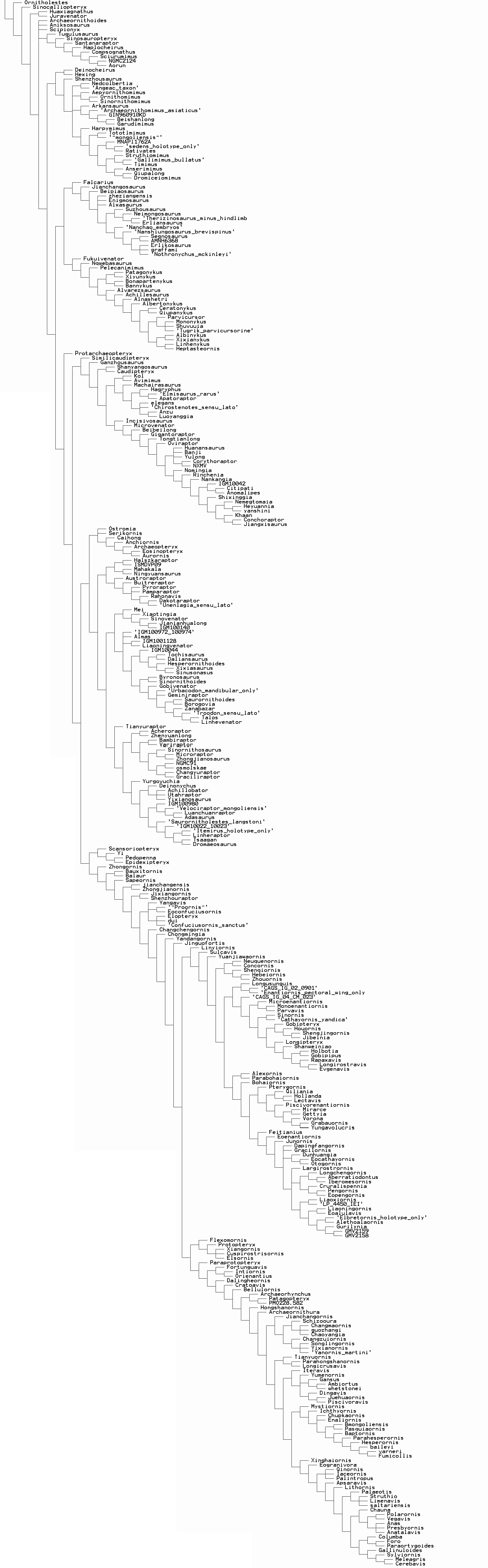


**Figure S2**. Strict consensus of 99999 most parsimonious trees (12123 steps, consistency index = 0.073, retention index = 0.589) with several taxa excluded a posteriori to increase resolution (see Positions of maniraptoromorphs pruned a posteriori below) and OTUs stemward of *Ornitholestes* not shown.

**Characters**

Characters are designed to incorporate all of those previously used in matrices using the Theropod Working Group (TWiG) as their base through 2012 with the exception of Senter (2011) a baraminology paper which was recognized too late in the coding cycle to be fully incorporated. Functionally, this led to all proposed TWiG maniraptoromorph characters through mid 2018 being used except 20 from Senter (2011), 102 from Brusatte et al. (2014) and 23 from eight other published analysis (see Fig. S1). We do note when characters from these newer analyses are the same as those we include, and provide commentary on their formulation and correlation with other characters. Note Turner et al.'s (2012) characters derive from Turner's (2008) thesis with at least two added and about eighty missing, each of which was listed in the thesis as "excluded because it has not been thoroughly examined." These mostly tyrannosaur-centric characters were left unscored in the thesis for taxa except *Allosaurus*, tyrannosauroids, *Compsognathus* and *Buitreraptor*, and were not explicitly examined in our matrix either. Similarly, Brusatte et al.'s (2014) characters derive from Brusatte's (2013) thesis. Although both versions have 853 characters, published character 853 is new while character 631 from the thesis is not in the published version. Thus their character numbers differ after character 630. In both Turner's and Brusatte's cases, we've used the character numbers from the published versions instead of the theses. Characters are for the most part listed in chronological order, then order within each publication. References are given to character number for each TWiG publication that independently added the character to their list or refined that character. Note that in many cases the character was previously used in a non-TWiG quantitative analysis (e.g. character 1 was first used by Zhou, 1999) and generally proposed to be phylogenetically useful prior to that. Characters not derived from TWiG analyses are referenced with their earliest known use.

Several characters are known to vary ontogenetically among Mesozoic theropods. These are noted under their descriptions and have been scored with 'N' in the NEXUS file if only young specimens can be coded. This prevents juveniles from being analyzed as adults and indicates the OTU was not merely left uncoded by accident. Unfortunately, TNT has no way to designate additional states equivalent to unknown, so the N codings were changed to ? in the TNT file.

1. Primary and secondary remiges - width of leading vane - longer or subequal to trailing vane (0); shorter than trailing vane (1) (1 in Norell et al., 2001; 55 in Xu, 2002; 878 in Gianechini et al., 2018). This specifies primaries and secondaries to the exclusion of coverts, tertials and/or alular feathers. Gianechini et al. added a new character scoring for the presence and symmetry of remiges, weighting the latter which was already character 1 in their matrix.

2. External naris - posterior extent with maxilla ventral border horizontal - ends anterior to antorbital fossa (0); extends posterior to anterior edge of antorbital fossa (1) (2 in Norell et al., 2001). This specifies state 1 which in its original form also included a naris subjectively "nearly reaching" the antorbital fossa.

3. Nasal - dorsolateral surface posterior to external naris - solid (0); pneumatized via fossae (1) (3 in Norell et al., 2001). This specifies the original subjective divide between "poorly" and "extensively" pneumatized nasals. It does not include pneumatization of the antorbital fossa, as occurs in carnosaurs. Brusatte et al. (2014) made that condition a second state, but it is separated here as character 502.

4. Maxilla - lateral surface of antorbital fossa anterior to antorbital fenestra - solid (0); with small maxillary fenestra entering maxillary antrum, greatest diameter <15% of orbit+jugal height (1); with medium maxillary fenestra 15-27% of orbit+jugal height (2); with large maxillary fenestra >27% of orbit+jugal height (3) (ordered) (4 in Norell et al., 2001; states 1 and 2 separated after 240 in Dal Sasso and Maganuco, 2011; states 2 and 3 separated after 27 in Turner et al., 2007b). Orbit+jugal height is used as an attempt at a neutral baseline for proportions of cranial features, which is also easy to estimate in even poorly preserved specimens. It is defined as the greatest distance between the jugal's ventral margin and the dorsal external margin of the orbit. This character formulation excludes the composite variables of "pronounced" and "round" (partly covered by character 340) in the original and quantifies maxillary fenestra size to be independent of antorbital fossa wall length (character 339) unlike Turner et al.. It similarly quantifies the "large" and "small" variables in Dal Sasso and Maganuco's character, which was the first to distinguish size only (instead of shape and position) between their states 0 and 4.

5. Maxilla - lateral surface at ventral margin of antorbital fossa - fully visible laterally (0); external surface projects dorsally to form a lip that overlaps ventral edge of antorbital fossa (1) (5 in Norell et al., 2001). This specifies the ventral rim to the exclusion of the anterior rim in its original form.

6. Maxilla - anteroposterior position of maxillary fenestra - close to anterior edge of antorbital fossa (distance between them <11% of orbit+jugal height) (0); far from anterior edge of antorbital fossa (>10%) (6 in Norell et al., 2001). This and other characters coding for the maxillary fenestra are also coded for the homologous maxillary fossa if such a structure is present. The state formulation corrects the original, as many taxa scored 0 do not have the maxillary fenestra strictly "at [the] rostral border of [the] antorbital fossa.

7. Maxilla - lateral surface at anterior margin of antorbital fossa - solid (0); with promaxillary fenestra entering promaxillary recess (1) (7 in Norell et al., 2001).

8. Orbit - length compared to height - >66% (0); <67% (1) (unknown in juveniles) (8 in Norell et al., 2001; 52 in Senter, 2011). This is quantified compared to Norell et al.'s original contrasting "round" to "dorsoventrally elongate" orbits. Brusatte et al. (2014) used the same quantification.

9. Postorbital - shape of anterior margin of ventral process - with projecting orbital process (0); smoothly curved or straight (1) (unknown in juveniles) (9 in Norell et al., 2001).

10. Jugal and postorbital - convexity of posterior edge of postorbital bar - concave to minimally convex (0-5% of orbit+jugal height) (0); strongly convex (>5%) (1) (10 in Norell et al., 2001). This quantifies the original character which had an unstated amount of constriction of the laterotemporal fenestra and included the extraneous specifier of "rectangular in shape" for state 0.

11. Suborbital fenestra - size - greatest diameter >24% of orbit height (0); <25% (1) (11 in Norell et al., 2001). Note contra Senter (2007), oviraptorids retain a suborbital fenestra, so an extra absent state for them is unneeded.

12. Braincase - separation of anterior tympanic and subotic recesses - separated by a surface which is not bounded by walls and depressed (0); both within a common lateral depression bounded on all sides (1) (12 in Norell et al., 2001; refined after 453 in Turner et al., 2012 and 6 in Zanno et al., 2009). This follows Turner et al.'s character 453 in excluding the often incorrect composite variables of crista prootica orientation (vertical in state 0), shape (crescent-shaped in state 1) and prominence (well-developed in state 1; here separated as character 438) in Norell et al.'s version. Contra Zanno et al.'s extra state, we do not recognize taxa with separated anterior tympanic and subotic recesses as having a lateral depression.

13. Basisphenoid - number of paired foramina in basisphenoid recess - none (0); at least one pair separated at midline (1) (13 in Norell et al., 2001). This excludes the composite variables of foramen placement (posterior), shape (circular) and degree of separation ("thin bar of bone") in Norell et al.'s original.

14. Opisthotic - lateral extent of interfenestral crest - confluent with lateral surface (0); depressed from lateral surface (1) (14 in Norell et al., 2001).

15. Exoccipital - convexity of posterior surface containing vagus (X), accessory (XI) and hypoglossal (XII) nerve foramina - flat to convex (0); concave, forming paracondylar recess (15 in Norell et al., 2001).

16. Basisphenoid - pneumatic invasion of dorsolateral surface of basipterygoid process - flat (0); excavated by an extension of the anterior tympanic recess (1) (16 in Norell et al., 2001).

17. Basisphenoid - pneumatic invasion of surface ventral to otic recess - excavated by subotic recess, an extension of the anterior temporal recess (0); flat (1) (17 in Norell et al., 2001). Note that as in Norell et al.'s original, the state of having a recess is 0, which could cause accidental misscorings as the plesiomorphic state for coelurosaurs seems to be 1.

18. Basioccipital and basisphenoid - position of basisphenoid recess - between basisphenoid and basioccipital (0); entirely within basisphenoid (1) (18 in Norell et al., 2001). Norell et al.'s original formulation also has a state 2 for "absent", but absence/presence is a different variable than position so is separated here as character 19. Brusatte et al. (2014) used Norell et al.'s formulation and noted it was left unordered "because there is not a clear nested set of primary homologies", but this also has the effect of hiding the homology between recesses entirely within the basisphenoid and those shared with the basioccipital.

19. Basisphenoid - pneumatic invasion of ventral surface - pneumatic median recess present (0); flat to convex (1) (18 in Norell et al., 2001).

20. Parasphenoid - transverse expansion of base of cultriform process - absent, process rodlike or vertically platelike (0); present (1) (19 in Norell et al., 2001). Norell et al. include the composite variable of pneumatization. While the expansion is due to a pneumatic recess in ornithomimosaurs and troodontines, other taxa can have pneumatic but slender cultriform processes, so that is not the variable being scored here.

21. Basisphenoid - transverse angle of basipterygoid process - less than 51 degrees laterally from vertical (0); >50 degrees (1) (20 in Norell et al., 2001). This quantifies the "lateroventrally" specifier of Norell et al.'s original, and excludes their mention of anterior angling in state 0. Turner et al. (2012) split the second state, coding ornithuromorphs as having horizontal processes, but since *Chauna* (DigiMorph Staff, 2001) has processes only 60 degrees from vertical, no further distinction is made here.

22. Basisphenoid - length of basipterygoid process - >7% of median occipital condyle height (0); <8% but present (1); absent (2) (ordered) (21 in Norell et al., 2001; states 1 and 2 separated after 281 in Turner et al., 2012). Our formulation quantifies Norell et al.'s original "well developed" and "abbreviated" qualifiers for states 0 and 1. Gianechini et al. (2017) deleted Turner et al.'s character leaving abbreviated and absent conditions scored the same.

23. Basisphenoid - pneumatic invasion of interior of basipterygoid process - absent, process solid (0); present, process hollow (1) (22 in Norell et al., 2001).

24. Prootic - pneumatic invasion of lateral surface - flat (0); excavated by shallow depression for dorsal tympanic recess (1); excavated by posterolaterally directed cavity for dorsal tympanic recess (2) (ordered) (23 in Norell et al., 2001). Note the dorsal tympanic recess is on the prootic, not the opisthotic as originally stated in Norell et al.'s character.

25. Opisthotic - pneumatic invasion of surface dorsal to interfenestral crest - flat (0); excavated by extension of posterior tympanic recess (1) (24 in Norell et al., 2001). Norell et al. used an additional ordered state for pneumatic invasion that is "extensive with indirect pneumatization", but this is not used here as only IGM 100/42 was coded that way, making it an autapomorphy.

26. Opisthotic - pneumatic invasion of lateral surface of paroccipital process or inside otic recess - flat (0); excavated by main diverticulum of posterior tympanic recess (1) (25 in Norell et al., 2001). Norell et al.'s original includes states for the position of the entrance of the posterior tympanic recess, which is here made into character 27 as absent is not a position.

27. Opisthotic - position of main entrance of posterior tympanic recess - on anterior surface of paroccipital process (0); opens into otic recess (1) (25 in Norell et al., 2001).

28. Premaxilla - length of subnarial process- does not contact nasal (0); contacts nasal, but does not extend posterior to external naris (1); extends posterior to external naris (2) (ordered) (26 and 27 in Norell et al., 2001). Norell et al.'s character 27 is the same as states 0/1 vs. 2 in their character 26 with the composite variable of "blunt" for its state 0.

29. Premaxillae - cross section of combined dorsal processes - transversely compressed or not compressed (0); dorsoventrally compressed (1) (28 in Norell et al., 2001). While traditionally only scored state 1 for a few maniraptoriform clades, many other theropods have dorsoventrally compressed combined processes as well, suggesting this might need to be refined in the future to only count a certain amount of compression. Note Senter (2011) incorrectly interpreted this character to indicate a straight dorsal margin over the naris in lateral view when he replaced it with his character 11 coding for naris proportions and angle.

30. Premaxillae - angle of symphysis in dorsal or ventral view - low, so that snout is pointed (0); high, so that snout is rounded (1) (29 in Norell et al., 2001).

31. Premaxilla - shape of ventral edge - smooth (0); crenulated (1) (30 in Norell et al., 2001).

32. Maxilla - posterior extent of palatal shelf - anterior edge of choana placed <18% down maxillary length (0); >17% (1) (31 in Norell et al., 2001). This follows Makovicky et al.'s (2005) redefinition to score for palatal shelf length instead of shelf composition. It also quantifies the previous "short" and "long" qualifiers. Brusatte et al. (2014) added a second state for the supposedly longer shelves of tyrannosauroids, ornithomimosaurs and *Austroraptor*, but this seems to reflect tooth size more than shelf length. For instance, *Gorgosaurus* has Brusatte's state 2 of a shelf "extending back at least to the level of alveolus 4" while *Dromaeosaurus* was specifically cited as an example which does "not possess the extremely elongate morphology of tyrannosauroids" and indeed has a shelf extending only to alveolus three. Yet in both cases the shelf extends to ~25% of maxillary length (Carr, 1999: Fig. 2I; Currie, 1995: Fig. 2D), its just that *Dromaeosaurus* has longer alveoli. Additionally, many small-toothed taxa (e.g. *Shuvuuia*, *Erlikosaurus*- covers 9 alveoli, *Xixiasaurus*- covers 15 alveoli) would clearly fall into their state 2 but are scored state 1, and how some taxa without maxillary teeth are scored state 1 (oviraptorosaurs, Aves) and others state 2 (ornithomimids) is unclear. In the absence of any clear difference in shelf length, and given tooth size is already scored as character 91, Brusatte et al.'s additional state is rejected.

33. Maxilla- ventral projection of posteromedial portion of palatal shelf - absent (0); present, forming 'tooth-like' process (1) (32 in Norell et al., 2001). Note we have scored this conservatively only for taxa whose posterior maxillary shelves are visible and not potentially hidden or broken.

34. Jugal - length of dorsal process - >34% of orbit+jugal height, measured from lower edge of orbit (0); <35% (1) (33 in Norell et al., 2001; refined after 32 in Dal Sasso and Maganuco, 2011). We agree with Dal Sasso and Maganuco in not combining lengths of the dorsal jugal process and ventral postorbital process (separated here as character 35) in one character, as there is a varying amount of overlap between processes. We have also quantified the character, although our boundary of 34% of orbitojugal height is slightly different from theirs of >50% of orbital height.

35. Postorbital - length of ventral process - height of orbit taken up by ventral process / orbital height <80% (0); >79% (1) (33 in Norell et al., 2001). We have quantified Norell et al.'s specifier of "ventrally elongate."

36. Jugal - cross section of posterior process - transversely compressed (0); dorsoventrally or not compressed (1) (34 in Norell et al., 2001). Note this allows scoring of dorsoventrally compressed processes unlike Norell et al.'s original, which was also slightly different in having a more restricted state 0- "twice or more as tall dorsoventrally as it is wide transversely."

37. Jugal - pneumatization of anterodorsal surface near antorbital fossa - present, surface invaded by pneumatic diverticulum of antorbital sinus (0); absent, surface solid (1) (35 in Norell et al., 2001).

38. Jugal - medial surface below postorbital process - penetrated by foramen (0); solid (1) (36 in Norell et al., 2001).

39. Quadratojugal - length of posterior process (defined as area posterior to intersection of ventral edge with line drawn along posterior edge of dorsal process) <36% of length of rest of quadratojugal (0); >35% (1) (37 in Norell et al., 2001). We have quantified this from Norell et al.'s original contrasting L shape with T or Y shape.

40. Quadratojugal - contact with jugal - sutured (0); fused (1) (unknown in juveniles) (38 in Norell et al., 2001).

41. Lacrimal - projection of dorsal surface - unprojected (0); with lateral ridge or boss projecting dorsally, forming lacrimal horn (1) (unknown in juveniles) (39 in Norell et al., 2001). While Li et al. (2010) added a state for a cornual process, coded only in tyrannosaurids, Carr (2005) indicates that this term is synonymous with a lacrimal horn as defined above.

42. Lacrimal - lateral projection at dorsal edge - unprojected or minimally projected (0); strongly projected (1) (39 in Norell et al., 2001; 732 in Brusatte et al., 2014). While originally included as a state of their lacrimal horn character by Norell et al., this is separated here as the lateral projection is not necessarily homologous with a dorsal horn. Brusatte et al. added a new character scoring for this when they already included it as a state of their character 37, weighting the condition.

43. Lacrimal - surface at posterodorsal corner of antorbital fossa - solid (0); pneumatized via foramen (1) (40 in Norell et al., 2001).

44. Lacrimal - length of posterior process measured from anterior orbital edge - <15% of ventral process length, measured from internal corner (0); 15-30% (1); >30% (2) (ordered) (41 in Norell et al., 2001; states 0 and 1 separated after 42 in Senter et al., 2012a). We have separated the lengths of anterior and posterior processes unlike Norell et al.'s composite character (see character 45), and have quantified the difference between "absent", short and T-shaped. Note Brusatte et al. (2014) incorrectly ordered their version of this character (39 in their analysis), making a present and unreduced process intermediate between an absent process and a reduced process.

45. Lacrimal - length of anterior process measured from internal corner - <131% of ventral process length, measured from internal corner (0); >130% (1) (41 in Norell et al., 2001; 374 in Xu et al., 2011a). We have quantified this and compared anterior process length to ventral process length instead of posterior process length as Norell et al. did, to keep characters 44 and 45 uncorrelated. While Xu et al. (2011a) also separated anterior and posterior process characters, their state 1 was an anterior process "extending anteriorly to [the] interfenestral bar" which requires a maxilla to score and depends on antorbital fenestra size.

46. Prefrontal - size - large, greatest length >34% of orbit+jugal height (0); small, <35% of orbit+jugal height (1); absent or fused to another element (2) (ordered) (42 in Norell et al., 2001). This quantifies Norell et al.'s version which used "dorsal exposure similar to lacrimal" for state 0, when lacrimal dorsal area depends on states 44 and 45 above, and is not often visible in specimens.

47. Nasals and frontals - angle of sutures from median to lateral edge of contact - anteriorly convex <60 degrees (0); anteriorly convex 60 degrees or more to posteriorly convex (1) (43 in Norell et al., 2001). We quantify Norell et al.'s original which merely contrasted wedge-shaped and transverse sutures, and also specify posteriorly convex taxa would be scored as state 1. Note for taxa which have irregular sutures, the angle is based on straight lines which extend from the anteromedian corner to the lateral edge of nasofrontal contact. The angle is based on the pair of frontals, not a single element. Taxa which have premaxillary-frontal contact are coded inapplicable.

48. Frontal - convexity of supratemporal fossa edge - completely posteriorly concave to straight (0); with posteriorly convex portion (1) (44 in Norell et al., 2001). This excludes the composite variable of extent on the postorbital process, which Norell et al. had in state 1.

49. Frontal - shape of orbital margin - straight to smoothly curved (0); abruptly angled at postorbital process (1) (45 in Norell et al., 2001).

50. Frontal - shape of anterior edge of nasolacrimal articular surface - smooth (0); notched anterolaterally (1) (46 in Norell et al., 2001). Note while this has been coded in the past for numerous taxa, the notch is in the edge overlapped by the nasal, so cannot be seen in fully articulated specimens.

51. Postorbital - curvature of anterior process - decurved to straight (0); upcurved (1) (47 in Norell et al., 2001).

52. Parietals - median proximity of supratemporal fossae - far, leaving flat dorsal surface between them (0); close, separated by ridge forming sagittal crest (1) (unknown in juveniles) (48 in Norell et al., 2001; 582 in Brusatte et al., 2014). Contra Norell et al., there does not appear to be an obvious difference in height between the parietal sagittal crests of tyrannosaurids and troodontids and those of other taxa. Thus their state 2 ("dorsally convex with well-developed sagittal crest") is combined into state 1, which now covers all taxa with sagittal crests. The composite variable of dorsal convexity in Norell et al.'s version is scored here as character 237. Brusatte et al. added an additional character scoring for the medial proximity of parietal supratemporal fossae, effectively weighting the character.

53. Parietals - median contact - sutured (0); fused (1) (unknown in juveniles) (49 in Norell et al., 2001).

54. Squamosal - angle of ventral process - <60 degrees from posterior quadrate edge (0); >59 degrees (1) (50 in Norell et al., 2001). This quantifies Norell et al.'s version which used "parallels" and "nearly perpendicular" as specifiers.

55. Squamosal - lateral surface - smooth (0); horizontal ridge over ventral process, extends to posterior process (1) (51 in Norell et al., 2001).

56. Quadratojugal - extent of squamosal contact - contacting (0); non-contacting (1) (52 in Norell et al., 2001).

57. Quadrate - divergence of otic articular surface - if present, articular surface confluent with shaft and squamosal head (0); distinct otic head caused by intercotylar incisure (1) (53 in Norell et al., 2001; 297 in Turner et al., 2012). Turner et al. based their character 351 on 36 of Norell and Clarke (2001), which separated the supposed primitive condition of a single articular surface with the supposed intermediate condition of having an unprojected but separate otic facet. However, the basal condition seems to be that present in many coelurosaurs where the otic facet is located far ventral on the quadrate shaft (e.g. *Daspletosaurus* in Fig. 28 of Currie, 2003). As this differs from Turner et al.'s coding and separating taxa with merged otic and squamosal surfaces from those with no otic surface is difficult, only the presence of a distinct otic head is considered here. The presence of braincase contact is then separated as character 564.

58. Quadrate - orientation of posterior edge compared to ventral margin of jugal + quadratojugal - inside angle <106 degrees (0); >105 degrees (1) (54 in Norell et al., 2001). This quantifies Norell et al.'s original character contrasting "vertical" and "strongly inclined" quadrates, and specifies anterodorsally inclined quadrates would be scored as state 0.

59. Quadrate - width from the paraquadrate foramen medial edge to the lateral edge of the dorsal quadrate - <30% of width across quadrate condyles (0); >29% (1) (55 in Norell et al., 2001). Note this is scored even if the dorsal quadratojugal process is too short to form a paraquadrate foramen. It quantifies Norell et al.'s character scoring for the presence of a lateral tab.

60. Quadrate - pneumaticity - solid (0); invaded by diverticulum of the tympanic airsac (1) (56 in Norell et al., 2001; 299 in Turner et al., 2012). Turner et al. also include Norell and Clarke's (2001) character 40 (as character 299), coding for a posteromedial quadrate foramen, but as that overlaps with the general quadrate pneumaticity character, is is not used here. Our character excludes the composite variable of a posterior quadrate fossa, specified by Norell et al. for state 1.

61. Foramen magnum - dorsoventral elongation - transversely elongate or height and width equal (0); height greater than width (1) (57 in Norell et al., 2001).

62. Exoccipital and basioccipital - lateral margin of occipital condyle - posteromedially sloped (0); constricted forming condylar neck (1) (58 in Norell et al., 2001). Note that unlike Brusatte et al.'s (2014) rewording of this character (their 53), we do not score for ventral constriction.

63. Exoccipital - length of paroccipital process - height halfway out from lateral edge of occipital condyle / length from lateral edge of occipital condyle to tip of process <71% (0); >70% (1) (59 in Norell et al., 2001). Norell et al.'s original character codes for both paroccipital process elongation and distal tapering, but as distinct dorsal and ventral edges are often curved or hard to define on the structure only newly quantified elongation is coded here. This is measured parallel to the long axis of the paroccipital process.

64. Exoccipital - orientation of paroccipital process - long axis angled ventrally <41 degrees from horizontal (0); >40 degrees (1) (60 in Norell et al., 2001). Unlike Norell et al.'s original, this is quantified and scores only for orientation, not curvature.

65. Exoccipital - torsion of paroccipital process - untwisted (0); twisted so that distal ends face posterodorsally (1) (61 in Norell et al., 2001).

66. Ectopterygoid - ventromedial surface - flat (0); excavated by a pneumatic fossa (1) (62 in Norell et al., 2001; 57 in Gohlich and Chiappe, 2006). Norell et al. defined state 0 as having a "constricted opening into [a] fossa" and scored it for oviraptorids, but such a structure has never been described or illustrated. Thus we redefine state 0 here, as if oviraptorids have a pneumatic ectopterygoid, it is not via an obvious fossa on the ventromedial surface as in most theropods.

67. Ectopterygoid - dorsal surface - flat (0); excavated by pneumatic fossa (1) (63 in Norell et al., 2001).

68. Pterygoid - size of posterolateral flange anterior to basipterygoid articulation- projects ventrolaterally from element (0); unprojected (1) (64 in Norell et al., 2001). Note Brusatte et al. (2014) rewords this character to refer to the "posterior flange for articulation with quadrate and epipterygoid" (character 553 here), but contra Norell et al.'s coding, this posterodorsal flange is well developed in *Shuvuuia*, *Erlikosaurus* and *Archaeopteryx*.

69. Palatine - development of jugal process - projected to form posterior concavity between it and main palatine body (0); unprojected (1) (65 in Norell et al., 2001).

70. Ectopterygoid - palatine contact - absent (0); present (1) (66 in Norell et al., 2001).

71. Dentary - medial curvature of symphyseal region - none (0); low (1); high (2) (ordered) (67 in Norell et al., 2001).

72. Surangular - shape of dorsal margin - smooth or with small coronoid eminence (0); with prominent coronoid eminence (1) (68 in Norell et al., 2001). While Norell et al.'s original character used "absent" for state 1, most taxa have low but present coronoid eminences (e.g. *Sinraptor dongi*- Currie and Zhao, 1994: Fig. 11E, between features 7 and 8; *Tyrannosaurus rex*- Brochu, 2003: Fig. 40A, directly above external mandibular fenestra).

73. Dentary - amount of anterior taper - anterior depth at symphysis <60% of maximum posterior depth (0); >59% (1) (69 in Norell et al., 2001). This quantifies Norell et al.'s original which contrasted "subtriangular" and "subparallel" states. Though Zanno et al. (2009) divided state 0 for the supposedly more tapered dentary of therizinosaurs, they are not consistently different from other coelurosaurs so the state isn't used here.

74. Dentary - curvature in lateral view - upcurved (0); straight (1); downcurved (2) (ordered) (70 in Norell et al., 2001; 262 in Zanno et al., 2009; 368 and 371 in Xu et al., 2011a; states 0 and 1 separated after 253 in Senter, 2007; 629 and 849 in Brusatte et al., 2014). Note while Senter (2007) divided decurved dentaries into two distinct states (gradual and weak throughout dentary vs. abrupt at anterior end), the latter state was coded almost entirely in taxa with short dentaries, such as oviraptorids. This creates the illusion of an abrupt curvature since it occurs over a shorter distance, but the curvature is actually quite weak in some such as *Rinchenia* and IGM 100/42. The only taxon with a long dentary Senter codes this way is *Neimongosaurus*, which does not preserve the posterior region so may actually be curved throughout the length of the bone. Zanno et al.'s and Xu et al.'s new characters 262 and 371 scoring for a concave ventral dentary margin (anteroventral in Zanno et al.'s case) are correlated with their characters 65 and 67 respectively scoring for a ventrally curved anterior dentary. Xu et al.'s character scoring for a convex dorsal dentary margin is similarly correlated with a decurved dentary. Foth et al. (2014; character 243) lost information by recombining straight and concave dorsal margins from Senter's version and proposed a new sigmoid state for caenagnathoids. Yet most theropods also have a sigmoid dorsal outline, but of a much more gradual variety since they lack the shortened dentaries of oviraptorids and extreme upturn of most caenagnathids. Thus only the main dentary curvature is scored for here.

75. Dentary - lateral surface of posterior dentary - roughly flat (0); with horizontal shelf (1) (71 in Norell et al., 2001). This specifies the posterior dentary as only that portion has a shelf in the therizinosaurs scored 1 by Norell et al..

76. Dentary - length of posterodorsal process - absent (0); less than half length of external mandibular fenestra, measured parallel to long axis of mandible (1); more than half length of external mandibular fenestra (2) (ordered) (72 in Norell et al., 2001; 303 in Turner et al., 2012). Note this refers to the process which contacts the external mandibular fenestra dorsally, not the process on the dorsal edge of the mandible. Turner et al. added their character 303 from Clarke's bird analysis, scoring for a posterior dentary that is "strongly forked with the dorsal and ventral rami approximately equal in posterior extent", but this is correlated with state 2 of the present character (68 in their analysis).

77. Dentary - position of lateral nutrient foramina - on flat surface (0); inside longitudinal groove (1) (73 in Norell et al., 2001). Norell et al.'s original character specified a "deep groove" for state 1, but considering the difficulty of objectively distinguishing shallow and deep grooves, any groove is scored for here.

78. Surangular - shape of external mandibular fenestra border - concave to evenly convex (0); with distinct process (1) (74 in Norell et al., 2001).

79. Prearticular - concavity of anteroventral margin - convex to straight (0); concave (1) (75 in Norell et al., 2001). Norell et al. used a character for internal mandibular fenestra shape ("small and slit-like" vs. "large and rounded"), but this is a composite created by both anteroventral prearticular shape and posterodorsal splenial shape (character 329 here). Turner et al. (2012) used both Norell et al.'s composite and a splenial character (their character 250) leading to correlated scores.

80. Surangular - size of posterior foramen - absent (0); small foramen (1); enlarged fenestra (2) (ordered) (76 in Norell et al., 2001; states 1 and 2 separated after 258 in Turner et al., 2012, 120 in Senter, 2011, 631 in Brusatte, 2013 and 72 in Brusatte et al., 2014). Note Senter (2011) changed this character to score for state 2 instead of states 1+2. Brusatte's thesis contains a character 631 scoring for a large surangular fenestra, which was deleted from the published version presumably because his character 72 already scored for this.

81. Splenial - lateral exposure - absent or narrow (0); broad (1) (77 in Norell et al., 2001).

82. Coronoid - size - large (0); small (1); absent (2) (ordered) (78 in Norell et al., 2001; 280 in Turner et al., 2012). Turner et al. added their character 280 from Clarke's bird analysis scoring for coronoid presence, but also included the present character (their character 76) thus scoring for coronoid absence twice.

83. Articular - dorsal surface of medial retroarticular process - on level with rest of element (0); projected dorsally into distinct process (1) (79 in Norell et al., 2001). This character differs from Norell et al.'s original in that they also included medial processes such as those in *Shuvuuia* and many birds and posteromedial processes, but they are not considered homologous here. It also eliminates the extraneous qualifiers of "slender" and "elongate" for the process.

84. Articular - retroarticular process elongation - taller than long to equidimensional (0); longer than tall (1) (80 in Norell et al., 2001).

85. Articular - dorsal surface of glenoid - concave to flat in lateral view (0); convex (1) (81 in Norell et al., 2001). Norell et al.'s character scores for the anteroposterior length of the mandibular glenoid, but we instead score for glenoid convexity as a more visible aspect of the caenagnathoid jaw apparatus.

86. Premaxilla - presence of teeth - toothed (0); toothless (1) (82 in Norell et al., 2001). While *Limusaurus* has recently been shown to lose all premaxillary, maxillary and dentary teeth as it aged, this is not true of most other edentulous theropods (e.g. *Sinornithomimus*, *Citipati*, *Gobipipus*) so is not considered an ontogenetically variable character here.

87. Premaxilla - size of second premaxillary tooth compared to third and fourth premaxillary teeth - smaller to equal in size (0); much larger (1) (83 in Norell et al., 2001).

88. Maxilla - presence of teeth - toothed (0); toothless (1) (84 in Norell et al., 2001).

89. Lateral teeth - serration extent - present on mesial and distal carinae (0); absent on mesial carinae but present on distal carinae (1); absent on mesial and distal carinae (2) (ordered) (unknown in very young juveniles) (85 in Norell et al., 2001). This character formulation differs from Norell et al.'s original in coding taxa with absent mesial serrations on only some teeth as being polymorphic instead of possessing another intermediate state. Xu (2002; character 118) adds yet another intermediate state to his version of the character, for taxa like microraptorians which have only some maxillary and dentary teeth lacking serrations entirely. Here the first few maxillary and dentary teeth are counted as anterior teeth to be scored along with the premaxillary teeth. Hwang et al. (2002- characters 63 and 64) divide Norell et al.'s original character into two- one for maxillary teeth and one for dentary teeth. Why this is done is uncertain, as every taxon in their matrix is coded the same for each even when one of the bones is unknown. *Troodon* sensu lato is a possible exception, as Currie (1987) described maxillary teeth as having mesial serrations while dentary teeth sometimes lacked them. Yet no maxilla has been described with teeth, making Currie's identifications based on size alone, and it's possible some of the smaller teeth are posterior maxillary teeth instead. It is also possible isolated teeth referred to *Troodon* belong to more than one taxon. Given this lack of demonstrable variation, both maxillary and dentary teeth are covered by character 89 here. This also allows taxa which preserve lateral teeth of uncertain placement to be coded, increasing information content. The coding of juveniles for this character applies only to extremely young individuals thought to be hatchlings or embryos (*Sciurumimus*, *Juravenator*, *Scipionyx*, *Archaeornithoides*, the Nanchao oviraptorids, *Scansoriopteryx*, IGM 100/972 and 100/974, *Zhongornis* and LP-4450-IEI), as *Lourinhanosaurus* and *Troodon* embryos show serrations develop with age.

90. Dentary - number of teeth - 1-11 (0); 12-24 (1); 25-30 (2); >30 (3) (ordered) (unknown in very young juveniles) (86 in Norell et al., 2001; states 0 and 1 separated after 85 in Senter, 2007, 80 in Foth et al., 2014 and 650 in Brusatte et al., 2014). This separates the composite variable of size in Norell et al.'s version as character 91 below. Senter's version of this character retained Norell et al.'s composite nature but added a state "small number of dentary teeth (?11)" without a size qualifier, which would not allow the small size of e.g. *Shenzhousaurus'* ~8-9 teeth to be homologized with taxa with numerous small teeth. He also added a state for toothless dentaries, which here is part of character 290. Foth et al. (2014) changed this to measure maxillary tooth number, with states <9, 9-21 and >21, state 1 being large and state 2 being small.

91. Lateral teeth - largest crown height - >15% of orbit + jugal height (0); 5-15% (1); <5% (2) (ordered) (86 in Norell et al., 2001; states 1 and 2 separated after 145 in Senter, 2011). Note Senter used a different quantification comparing crown height to snout height and dentary ramus height.

92. Teeth - serration size - <26 on distal carina (0); >25 (1) (87 in Norell et al., 2001). While Norell et al. cited Farlow et al. (1991) as the quantification between their "large" and "small" serration states, that publication merely has a 95% Confidence Interval equation for theropod teeth in their database excluding those referred to *Troodon* and *Spinosaurus*. To avoid statistical uncertainty and the need to analyze FABL and serration length using their model, we instead quantify this based on the number of serrations that could fit on the distal crown edge. Note the state order could lead to misscoring since state 1 is primitive for coelurosaurs.

93. Teeth - distal serration shape - symmetrically rectangular to rounded (0); hooked apically (1) (88 in Norell et al., 2001). This eliminates Norell et al.'s "large" qualifier for state 1 which is covered by character 92 above, and the "and often mesial edges" qualifier that does not always apply.

94. Teeth - crown root transition - constricted mesial and/or distal edges (0); undifferentiated mesial and distal edges (1) (89 in Norell et al., 2001; 283 in Li et al., 2010; 754 in Brusatte et al., 2014). Hwang et al. (2002- character 68 and 69) divide Norell et al.'s original character into two- one for maxillary teeth and one for dentary teeth. As with their characters 63/64 discussed above, every taxon is coded the same for each even when one of the bones is unknown (except *Allosaurus* and *yanshini*, which are coded unknown for the dentary tooth character this time). While several taxa do show variation in tooth constriction, this is usually between anterior and posterior teeth (e.g. *Protarchaeopteryx*, *Microraptor*) or within a single element (e.g. *Jinfengopteryx*, *Archaeopteryx*), not between elements. This and the potential loss of information when coding taxa with isolated teeth led to combining codings for maxillary and dentary teeth here. Li et al.'s character is a composite also adding curvature as a condition of state 0. Note the state order could lead to misscoring since state 1 is primitive for coelurosaurs.

95. Dentary teeth - size of anterior teeth - smaller than posterior two thirds of teeth (0); similar to posterior third of teeth, leaving teeth in the middle third the largest (1); larger than posterior two thirds of teeth (2) (ordered) (90 in Norell et al., 2001; state 2 added from 231 in Kirkland et al., 2005, 286 in Li et al., 2010 and 265 in Zanno et al., 2009). Norell et al.'s original was composite in scoring for both size and spacing (separated as character 96 here) and only for anterior teeth being smaller and more closely spaced. Kirkland et al., Zanno et al. and Li et al. independently added separate characters for anterior teeth being larger.

96. Dentary teeth - difference in spacing between anterior and posterior teeth - anterior more widely spaced (0); subequal spacing (1); anterior more closely spaced (2) (ordered) (90 in Norell et al., 2001; states 0 and 1 separated after 89 in Senter, 2010).

97. Premaxilla, maxilla and dentary - medial bone between teeth - undifferentiated from interdental septa and/or main body of element (0); forms distinct interdental plates (1) (91 in Norell et al., 2001). Xu (2002- character 124) adds an additional state distinguishing absent and fused interdental plates, but the distinction between these conditions has been historically contentious and is not evaluated here, as in most TWiG matrices.

98. Dorsal vertebrae - separation of postzygapophyses - joined into median lamina (0); separated over neural canal (1) (92 in Norell et al., 2001). We do not use Norell et al.'s composite variable of hyposphene transverse position, overlapping vs. lateral to the neural canal. As per Brusatte et al.'s (2014) commentary on this character (his 103), state 1 also covers taxa which have dorsally separated hyposphenes whitch join ventrally via a horizontal lamina.

99. Premaxillary teeth - convexity of lingual surface - subequally or more convex compared to labial surface (0); much flatter to concave (1) (93 in Norell et al., 2001; 345 in Zanno et al., 2009; 136 in Senter, 2011). Contra Senter (2007; 138 in Senter, 2011), the condition in *Protarchaeopteryx* and *Incisivosaurus* where only the first pair of teeth are apomorphic is considered polymorphic, not a separate unordered state. Zanno et al. created new character 345 for incisiform anterior teeth, so score for this morphology twice.

100. Cervical vertebrae - number - ten or less (0); eleven or more (1) (94 in Norell et al., 2001). Note Norell et al.'s states of "10" vs. "12 or more" do not let taxa with eleven cervicals be scored, a problem that persists at least through Brusatte et al. (2014).

101. Cervical vertebrae - posterior extent of epipophyses on axis - does not extend past postzygapophyses (0); extend past postzygapophyses (1) (95 in Norell et al., 2001). Note this may be scored as derived in taxa without preserved axes if more posterior cervicals have posteriorly extensive epipophyses, as epipophyseal size increases anteriorly in all known examples.

102. Axis - development of spinopostzygapophyseal lamina - deep, broadly connecting neural spine with postzygapophyses (0); narrow to absent, so that neural spine and postzygapophyses are separated (1) (96 in Norell et al., 2001; 263 in Turner et al., 2012). This is usually phrased as an issue of neural spine compression, but even taxa with state 0 can have narrow, blade-like spines. Turner et al. (2012) added their new character 263 contrasting "sheetlike" and "anteroposteriorly reduced and rodlike" spines, which is the same as this character (their character 94) contrasting spines that are "compressed mediolaterally" with those that are "flared transversely", and so weighted this morphology. Note also that Norell et al.'s wording of "flared" for state 0 implies dorsal transverse expansion, which is scored for in character 508 here.

103. Cervical vertebrae - anteroposterior position of epipophyses - partly over postzygapophyseal facets (0); anterior to postzygapophyseal facets (1) (97 in Norell et al., 2001).

104. Cervical vertebrae - posterior extent of centrum on cervicals 3-4 - completely under neural arch (0); extending posteriorly past neural arch (1) (98 in Norell et al., 2001). The vertebrae are measured with the neural canal horizontal. This specifies Norell et al.'s "anterior" cervicals.

105. Cervical vertebrae - anteroventral surface of cervicals 9-10 - convex or flat (0); transversely concave due to carotid processes (1) (99 in Norell et al., 2001). This specifies Norell et al.'s "posterior" cervicals.

106. Cervical vertebrae - anterior central articular surface dimensions of cervicals 3-4 - less than 1.71 times wider than tall (0); over 1.70 times wider than tall (1) (100 in Norell et al., 2001). Our formulation eliminates Norell et al.'s composite variable of median constriction ("kidney-shaped") in state 1, quantifies "distinctly wider than high" and specifies "anterior" cervicals.

107. Cervical vertebrae - neural spine anteroposterior length of cervicals 3-5 - length / neural arch length excluding epipophyses >29% (0); <30% (1) (101 in Norell et al., 2001). We specify which cervicals qualify compared to Norell et al.'s version, ignore the composite variable of how centered the neural spine is on the neural arch, and quantify how short the spines must be.

108. Cervical vertebrae - number of pneumatic foramina in each centrum - one pair anteriorly located behind parapophyses (0); two pairs (1) (102 in Norell et al., 2001).

109. Cervical vertebrae - convexity of anterior articular surface - flat or concave (0); convex (1); convex dorsoventrally, concave horizontally (2) (unordered) (103 in Norell et al., 2001; state 2 after 101 in Turner et al., 2007b). Note that contra Brusatte et al.'s (2014) version of this character (their 99), this analysis counts weakly convex articular surfaces as different from flat surfaces. We also separate cervical and anterior dorsal centra, the latter scored in character 110, based on taxa such as *Monolophosaurus*, *Megaraptor* and *Compsognathus* which have different anterior convexities for each.

110. Dorsal vertebrae - convexity of anterior articular surface of centra 1-3 - flat or concave (0); convex (1); convex dorsoventrally, concave horizontally (2) (unordered) (103 in Norell et al., 2001; state 2 added from 101 in Turner et al., 2012).

111. Dorsal vertebrae - hypapophysis size on dorsals 1-3 - hypapophyses absent or all less than 25% of posterior central articular surface height (0); at least one hypapophysis more than 25% of posterior central articular surface height (1) (104 in Norell et al., 2001). This quantifies "large" hypapophyses from Norell et al.'s character.

112. Dorsal vertebrae - lateral projection of parapophyses on dorsals 6-13 - parapophysis width measured from ventromedial corner <20% of centrum width (0); >19% (1) (105 in Norell et al., 2001). We quantify Norell et al.'s "distinctly projected" qualifier for state 1. For this and other characters coding posterior dorsals (6-13 primitively for avepods), the posterior half of the dorsal series is compared in taxa with reduced dorsal counts (e.g. 3-6 in *Meleagris* which has six dorsals).

113. Dorsal vertebrae - orientation of medial postzygapophyseal edge - undifferentiated from postzygapophyseal facet (0); bent ventrally to form hyposphene which articulates with hypantrum on previous vertebra (1) (106 in Norell et al., 2001). Note this is sometimes absent in the anteriormost dorsals (e.g. first two in *Dromiceiomimus*- Makovicky, 1995), so should not be scored state 0 if only anterior dorsals are known.

114. Dorsal vertebrae - extent of central pleurocoels in series - absent (0); only present anteriorly (1); present in anterior and posterior dorsals (2) (ordered) (107 in Norell et al., 2001; 106 in Senter, 2007; 106 in Dal Sasso and Maganuco, 2011; states 0 and 1 separated after 265 in Turner et al., 2012 and 106 in Zanno et al., 2009). Note this does not include large central fossae or grooves as found in some avialans, which have an uncertain homology with central foramina. Norell et al.'s original character had no state for taxa with only some pleurocoelus dorsals, while Senter's revision only scores for middle and posterior dorsals. Turner et al. and Zanno et al. independently fix Norell et al.'s version. Dal Sasso and Maganuco's revision of Senter's character unfortunately simplifies it to pleurocoelous versus non-pleurocoelous dorsals, so loses information.

115. Dorsal vertebrae - lateral extent of transverse processes on dorsals 1-4 - project far beyond prezygapophyses laterally (0); project slightly past prezygapophyses laterally (distance from midline to transverse process tip / distance from midline to prezygapophysis tip <180% for d1, <200% for d2, <215% for d3 and <250% for d4) (1) (108 in Norell et al., 2001). We quantify Norell et al.'s "long" and "short" qualifiers, and exclude the composite variables of "thin" versus "wide" and state 1 being "only slightly inclined."

116. Dorsal vertebrae - transverse width of neural spine distal tip - <151% of minimum neural spine width (0); >150% (1) (109 in Norell et al., 2001). Norell et al.'s original version has "not expanded" as state 0, but most taxa scored that way actually have slight expansions on at least some dorsals so we use a different quantification.

117. Sacral vertebrae - number - 5 or less (0); 6 (1); 7 (2); 8 (3); 9 (4); 10 (5); 11-14 (6); >14 (7) (ordered) (111 in Norell et al., 2001; 105 in Foth et al., 2014; states 2 and 4 after 110 in Turner et al., 2007b; states 5-7 after 110 in Turner et al., 2012). This codes for the number of vertebrae fused together, when this differs from the number contacting the ilia (e.g. *Nomingia*). While some taxa with six sacrals seem to add a dorsal, others add a caudal. This makes the states potentially non-homologous, but the exact condition is known in so few taxa that much information would be lost if the state were divided into the number of dorsosacrals and caudosacrals present. Norell et al.'s original character has no state for taxa with less than five or with seven sacrals, while Turner et al.'s (2007b) revision adds a state for seven sacrals but lacks a state for ten or more sacrals. Turner et al. (2012) fixed the latter issue when adding Clarke's states for derived birds, and also fixed state 0 to include taxa with less than five sacrals. Foth et al. (2014) removed information from the character by recombining 7-10 sacrals as a single state.

118. Sacral vertebrae - ventral surface of posterior centra - transversely rounded (0); transversely flattened with median groove on some (1); with median keel on some (2) (unordered) (112 in Norell et al., 2001). This excludes the composite variable of "strongly constricted transversely" for state 2 of Norell et al.'s character.

119. Sacral vertebrae - number of vertebrae with central pneumatic foramina - none (0); at least one vertebra but not present in all (1); all (2) (ordered) (113 in Norell et al., 2001). We slightly redefined state 1 of Norell et al.'s character (originally "present

on anterior sacrals only") to account for taxa with e.g. sacrals 1-4 being pleurocoelous or only posterior sacrals being pleurocoelous.

120. Sacral vertebrae - convexity of posterior articular surface of last sacral centrum - concave to flat (0); convex (1) (114 in Norell et al., 2001). This adds "concave" to state 0 to account for taxa with that condition.

121. Sacral vertebrae - dorsal connection of zygapophyses - each zygapophyseal articulation distinct from that of adjacent vertebrae (0); zygapophyseal articulations joined to form ridge (1) (115 in Norell et al., 2001). This excludes the composite specifier of fused zygapophyses from state 1, found in Norell et al.'s original.

122. Caudal vertebrae - number with transverse processes of any size - eleven or more (0); seven to ten (1); less than seven (2) (ordered) (117 in Norell et al., 2001; states 1 and 2 separated after 116 in Makovicky et al., 2005). Note this includes any amount of lateral projection, not merely well developed processes. Norell et al.'s original version scored for the number of caudals before the transition point, which we have specified to those with transverse processes. We do this because other variables like centrum elongation, neural spine height and chevron morphology will often change at different points along the caudal series.

123. Caudal vertebrae - transverse section of proximal centra - rounded (0); rectangular (1) (118 in Norell et al., 2001). This eliminates the composite variable of "tall" from state 0, found in Norell et al.'s version. Their character also had a state 2 scoring for "anterior caudal centra laterally compressed with ventral keel" which we have separated as character 124.

124. Caudal vertebrae - ventral surface of centra 1-2 - convexly rounded, flat or grooved (0); with a median keel (1) (118 in Norell et al., 2001). As in character 123, we eliminate Norell et al.'s variable "laterally compressed" for state 1.

125. Caudal vertebrae - dorsal outline of neural spines in caudals ~5-30 - convex or flat (0); concave to form accessory anterior spine (1) (119 in Norell et al., 2001). Since the exact caudals with accessory spines varies between taxa (e.g. 13-28 in *Tyrannosaurus* vs. 5-12 in *Sinosauropteryx*) a representative number of caudals in the range of 5-30 is necessary to score a taxon as lacking this character. This and other characters involving mid and distal caudals and chevrons are coded inapplicable for taxa with extremely short tails and pygostyles such as pygostylians.

126. Caudal vertebrae - transverse convexity of dorsal midline in distal caudals - narrowly convex to form neural spine (0); flat with no spine or groove (1); concave to form median groove (2) (ordered) (120 in Norell et al., 2001). Contrary to Brusatte et al.'s (2014) interpretation, we score this for distal caudals (e.g. through at least caudal 36 in *Tyrannosaurus*, and at least caudal 26 in *Gallimimus*).

127. Caudal vertebrae - length of longest prezygapophyses in distal caudals - <33% of centrum length (0); 33-100% of centrum length (1); >100% of centrum length (2) (ordered) (121 in Norell et al., 2001).

128. Caudal vertebrae - number - >40 (0); 25-40 (1); 21-24 (2); <21 (3) (ordered) (122 in Norell et al., 2001; states 2 and 3 separated after 121 in Senter, 2007 and 116 in Foth et al., 2014). Taxa with extremely short tails and pygostyles such as pygostylians are coded as state 3 based on extremely young examples such as enantiornithine IVPP V14238 even though generally the number of vertebrae incorporated into the pygostyle cannot be determined. Foth et al. added a state for <17 caudal vertebrae, which is equivalent to our state 3 as regards taxon scoring except for *Eosinopteryx* (20 caudals).

129. Chevrons - anteroposterior length compared to dorsoventral depth - <76% through chevron 10 (0); >75% by chevron 10 (1) (123 in Norell et al., 2001). This only scores for length versus height and not cross section ("cylindrical" or "flattened and plate-like") unlike Norell et al.'s version.

130. Chevrons - transverse convexity of anterior ventral edge in distal elements - convex to straight (0); concave to form bifurcated anterior ventral process (1) (124 in Norell et al., 2001). This is inapplicable if taxa lack anterior ventral processes (character 400).

131. Cervical ribs - length - greater than centrum (0); equal or less than centrum (1) (125 in Norell et al., 2001). This eliminates the composite variables "slender" versus "broad" from Norell et al.'s version.

132. Uncinate processes - ossification - unossified or absent (0); ossified (1) (unknown in juveniles) (126 in Norell et al., 2001). This and the following character are only considered absent if the specimen is relatively complete and well articulated in the appropriate area.

133. Sternal ribs - ossification - unossified or absent (0); ossified (1) (unknown in juveniles) (127 in Norell et al., 2001).

134. Gastralia - length of lateral segments - shorter than medial segment (0); longer (1) (128 in Norell et al., 2001).

135. Sternum - fusion of sternal plates and ventral projection of median surface - sternal plates unfused, without ventral keel (0); sternal plates fused, without ventral keel (1); sternal plates fused, with low ventral keel (2); sternal plates fused, with tall ventral keel (3) (ordered) (unknown in juveniles) (129 in Norell et al., 2001; states 2 and 3 separated after 326 in Turner et al., 2012). Norell et al. originally scored taxa with sternal keels as 2, though they did not define this state. While this character is unusual in combining two varying traits, developmental biology suggests "the formation of the keel must always lag behind fusion of the sternal midline" (O'Connor et al., 2015). It is thus provisionally left as a composite character.

136. Sternum - shape of lateral edge posterior to rib articulations - evenly rounded or straight (0); distinct posterolateral process present (1) (unknown in juveniles) (130 in Norell et al., 2001).

137. Sternum - surface of coracoid articulation - concave dorsoventrally forming groove (0); flat to convex (1) (132 in Norell et al., 2001).

138. Furcula - size of hypocleideum - absent (0); small ridge or tubercle (1); large process (2) (ordered) (133 in Norell et al., 2001; states 1 and 2 separated after 132 in Turner et al., 2012 and Senter, 2010).

139. Scapula - eversion of dorsal edge of acromion - absent, acromion completely dorsally to medially projected (0); present, edge of acromion laterally projected (1) (134 in Norell et al., 2001; 243 in Xu et al., 2009). Note all scapular characters assume a horizontal long axis, although it would be diagonal in life. Foth et al. (2014; character 128) added "or hooked" to state 1, but that is just a form of lateral projection so is not specified here.

140. Humerus - length - less or equal to scapula (0); greater than scapula (1) (135 in Norell et al., 2001).

141. Coracoid - transverse convexity of posterior edge (anteroposterior convexity of lateral edge in Aves) - convex to flat (0); concave forming subglenoid fossa (1) (136 in Norell et al., 2001). This excludes the composite qualifier of "triangular" for the subglenoid fossa and the specification that the fossa is bounded by the coracoid tuber, as there are taxa like *Aerosteon* that have a fossa but no tuber. Note all coracoid characters assume a posterior glenoid edge for taxa without elongate coracoids, though this would have been largely laterally-facing in taxa with bent coracoids such as most paravians. For taxa which reduce the proximal coracoid body such as crown birds, the alternative description assuming lateral and medial edges is provided in parentheses.

142. Scapulocoracoid - form of contact between components - sutured or freely articulated (0); fused (1) (unknown in juveniles) (137 in Norell et al., 2001).

143. Coracoid - proximodistal elongation - proximodistal depth <91% of anteroposterior length from anterior edge to glenoid (mediolateral length from medial edge to glenoid in Aves) (0); 91-200% (1); >200% (2) (ordered) (138 in Norell et al., 2001; states 1 and 2 separated after 136 in Senter, 2007 and 136 in Turner et al., 2007b; 682 in Brusatte et al., 2014). Note the anteroposterior length excludes the posterodistal process. This excludes the composite variables of "subcircular" versus "subquadrangular" shape (scored here as character 336) and "elongate posteroventral process" found in Norell et al.'s original.

144. Coracoid - angle formed in posterior (lateral in Aves) view at level of coracoid tubercle - <31 degrees (0); >30 degrees (1) (139 in Norell et al., 2001). Taxa with no significant portion of the coracoid proximal to the coracoid tubercle (character 607) are coded as inapplicable. This quantifies Norell et al.'s character, which phrased the difference as abruptness of the bend instead of the more easily quantified angle.

145. Scapula - orientation of glenoid - mostly on ventral edge (0); wraps onto lateral surface (1) (140 in Norell et al., 2001). Like Senter's (2007) version of this character, this codes for glenoid orientation compared to the scapular blade, not the orientation compared to the body.

146. Humerus - angle between distal deltopectoral crest edge at apex and posterior edge of shaft at that point - >85 degrees (0); 46-85 degrees (1); <46 degrees (2) (ordered) (141 in Norell et al., 2001). This quantifies the "triangular", "quadrangular" and "arc"-shaped crests described by states in Norell et al.'s character. Their character also included a number of composite variables covered by characters 147 and 148 here.

147. Humerus - proximodistal placement of deltopectoral crest apex - <30% of element length (0); 30-50% (1); >50% (2) (ordered) (141 in Norell et al., 2001; 684 in Brusatte et al., 2014; refined by 252 in Xu et al., 2009 and 286 in Zanno et al., 2009). This measures to the most projected tip of the process, not its entire distal extent. It quantifies Norell et al.'s state 3 of "extremely long" for alvarezsaurids. Xu et al. added a character that quantified deltopectoral crest length but left a gap for crests 25-30% of humeral length, while Zanno et al. added a character with states scoring for crests <25% and >50% of humeral length. Brusatte et al. used 25% and 35% as the cut-offs between states and had no state for crests >50% of humeral length.

148. Humerus - depth of deltopectoral crest - width of humerus at deepest part of deltopectoral crest <301% minimal shaft depth (0); >300% (1) (141 in Norell et al., 2001; 364 in Turner et al., 2012). This quantifies Norell et al.'s states scoring for "large and distinct", "less pronounced" and "very weakly developed" crests from state 4, "extremely broad" crest. Turner et al. added a correlated character from Clarke's bird analysis scoring for crests shallower, equal and deeper than humeral shaft width. Yet scaling even seemingly divergent taxa like *Gallimimus* and *Shuvuuia* to the same shaft width results in crests of similar depth (196% vs. 193%). Thus only a state coding for hypertrophy as in *Mononykus* and *Confuciusornis* is retained here.

149. Humerus - lateral surface of distal deltopectoral crest - flat (0); with longitudinal ridge (1) (142 in Norell et al., 2001).

150. Ulna - length of olecranon process - proximal tip to apex of coronoid process compared to length of element <15% (0); 15-25% (1); >25% (2) (ordered) (143 in Norell et al., 2001; states 0 and 1 separated after 142 in Senter, 2007). This quantifies Norell et al.'s qualifiers of "weakly developed" and "distinct and large" and Senter's "hypertrophied" and "not hypertrophied."

151. Ulna - transverse convexity of distal end - rather straight to concave (0); highly convex (1) (144 in Norell et al., 2001; 259 in Xu et al., 2009; 765 in Brusatte et al., 2014). This would ideally be quantified, but objective landmarks for the proximodistal baseline for convexity are difficult to define. Xu et al. added an identical correlated character.

152. Ulna - separation of proximal cotylae - single cotyla (0); separate cotylae (1) (145 in Norell et al., 2001). Contrary to Norell et al.'s original wording, cotyla are not always concave, nor are they always separated by a ridge as opposed to a groove.

153. Manual phalanx I-1 - shape of proximoventral edge - convex to flat (0); concave due to flexor tubera (1) (768 in Brusatte et al., 2014). This specifies Brusatte et al.'s character to phalanx I-1, the only scorable phalanx in alvarezsaurids.

154. Distal carpals I and II - form of contact - unfused (0); fused (1) (unknown in juveniles) (147 in Norell et al., 2001). This eliminates any reference to which metacarpals each distal carpal contacts, found in Norell et al.'s version. While Norell et al. were neutral with regard to the homology of the avian semilunate, Xu et al. (2014) have shown it is formed by a fusion of distal carpals.

155. Distal carpal I - form of contact with metacarpal I - unfused (0); fused (1) (unknown in juveniles) (148 in Norell et al., 2001). Norell et al. used a character scoring for distal carpals fused to metacarpals, but we divide this as e.g. some alvarezsaurids fuse only metacarpal I, while e.g. some basal birds fuse only metacarpals II and III.

156. Distal carpal II - form of contact with metacarpal II - unfused (0); fused (1) (unknown in juveniles) (148 in Norell et al., 2001).

157. Distal carpals I and II - combined transverse width - covers >74% of proximal transverse width of metacarpals I and II (0); <75% (1) (149 in Norell et al., 2001). This quantifies Norell et al.'s character which distinguishes semilunate carpals "covering all

of proximal ends of metacarpals I and II", "covering about half of base of metacarpals I and II" and "covers bases of all metacarpals." The latter state was only scored in *Shuvuuia* and *Mononykus*, but these actually have less extensive semilunate carpals (Perle et al., 1994: Fig. 13B-C; IGM 100/975). This and other manual characters assume a manus flexor side that is ventral, though in life the flexor side faced medially due to the inability to pronate.

158. Metacarpal I - length compared to metacarpal II - <50% (0); 50-65% (1); 66-80% (2); >80% (3) (ordered) (150 in Norell et al., 2001; states 1 and 2 separated after 149 in Senter, 2010). Norell et al.'s original character lacks states scoring for taxa whose metacarpal I is between half the length of and subequal to metacarpal II, or those whose metacarpal I is longer than subequal. The former gap was addressed by Senter who added a state for "about two-thirds", which along with the term subequal is not quantified well. We have quantified all states precisely. State 1 is almost equivalent to Brusatte et al.'s (2014) new state of ~50-70% in their character 146.

159. Manual digit III - number of phalanges - four (0); three (1); two (2); one (3); none (4) (ordered) (151 in Norell et al., 2001; states 1/2/3 separated from 4 after 301 in Senter, 2007; states 1-3 separated after 147 in Cau et al., 2015). The original version used by Norell et al. only scored for phalanges present versus absent as in tyrannosaurids, while Senter added a correlated character scoring for four versus less than four phalanges. As tyrannosaurids were scored unknown by Senter, this hid potential homology between partial and complete loss of phalanges on that digit. We combine these states into a single character, and add the intermediate states which were first used in a TWiG analysis by Cau et al..

160. Manual unguals - proximodistal position of flexor tubercle apex - <25% down length of ungual (measured on the ventral curve, excluding the tubercle) (0); >24% (1) (152 in Norell et al., 2001; refined in 151 in Senter, 2007, 151 in Zanno et al., 2009 and 740 in Brusatte et al., 2014). Norell et al. initially used a composite character scoring for ungual curvature, flexor tubercle size and flexor tubercle proximodistal placement. Zanno et al. separated weakly curved and straight unguals and added a state for weakly curved unguals with low and proximally placed tubercles, but their character is still composite and lacks states for five of the nine possible permutations. We follow Senter in separating these variables (see characters 161-164), but his third state of "proximodistally elongated with proximal end close to articular facet" for basal ornithomimosaurs is not used here, as this absent in *Shenzhousaurus*, only present in ungual II of *Harpymimus*, and not verifiable in published photos of *Pelecanimimus*. We also quantify proximodistal placement for the first time.

161. Manual ungual I - curvature, measured as maximum distance between dorsal edge and line drawn between proximodorsal and distoventral tips divided by length of latter line - >16% (0); 10-16% (1); <10% (2) (ordered) (152 in Norell et al., 2001; states 1 and 2 separated after 151 in Hwang et al., 2004 and 151 in Zanno et al., 2009; refined after 298 in Senter, 2007). As noted above, Norell et al.'s original character was a composite of three variables, which Zanno et al. expanded in part by Zanno et al. in separating straight and weakly curved unguals. Hwang et al. independently separated straight and weakly curved unguals but only had states for three of the nine possible permutations. Senter separated characters scoring for the curvature of manual ungual I and unguals II-III, which we follow and expand upon here. We also quantify this for the first time.

162. Manual ungual II - curvature, measured as maximum distance between dorsal edge and line drawn between proximodorsal and distoventral tips divided by length of latter line - >16% (0); 10-16% (1); <10% (2) (ordered) (152 in Norell et al., 2001; states 1 and 2 separated after 151 in Hwang et al., 2004 and 151 in Zanno et al., 2009; refined by 299 in Senter, 2007). The discussion for character 161 applies, with the addition that we divided Senter's character scoring for the curvature of unguals II and III into a character for each ungual (162 and 163).

163. Manual ungual III - curvature, measured as maximum distance between dorsal edge and line drawn between proximodorsal and distoventral tips divided by length of latter line - >16% (0); 10-16% (1); <10% (2) (ordered) (152 in Norell et al., 2001; states 1 and 2 separated after 151 in Hwang et al., 2004 and 151 in Zanno et al., 2009; refined by 299 in Senter, 2007). The discussion for character 162 applies.

164. Manual unguals - depth of flexor tubercles - >32% of articular facet height (0); <33% (1) (152 in Norell et al., 2001; 475 in Brusatte et al., 2014; refined by 348 in Senter, 2007). The flexor tubercle is measured from the ventral edge of the articular facet. As noted for character 160, Norell et al.'s original character was a composite of three variables which were separated by Senter. We follow Senter's quantification.

165. Manual ungual I - size compared to manual ungual II - smaller (0); equal to three times larger (1); over three times larger (2) (ordered) (153 in Norell et al., 2001; states 0 and 1 separated after 152 in Zanno et al., 2009). This is based on length, or with proximal height as a proxy, though these can be different in fringe cases of highly disparate anatomy between digits. It can often be coded based on penultimate phalanx size. State 2 quantifies the "large" variable in Norell et al.'s original character, while states 0 and 1 follow Zanno et al.'s modification adding a state for ungual II being largest.

166. Manual ungual I - shape of proximodorsal margin - smoothly convex (0); with lip rising dorsally (1) (154 in Norell et al., 2001; refined after 153 in Senter, 2007 and 153 in Zanno et al., 2009). Norell et al.'s original character merely scores for lips on "some manual unguals", which is potentially non-homologous if e.g. one taxon has a lip on ungual I only and another taxon on ungual II only, but both taxa would be scored as state 1. Zanno et al. changed the states to be absent on all unguals, present only on unguals II and III, and present in all unguals. Yet there are taxa which only have a lip on ungual I, like *Deinocheirus* and *Scansoriopteryx*, which are impossible to score in this formulation. Senter improved Norell et al.'s composite character by scoring for a lip on ungual I in a separate character, which is followed here.

167. Manual ungual II - shape of proximodorsal margin - smoothly convex (0); with lip rising dorsally (1) (154 in Norell et al., 2001; refined by 300 in Senter, 2007 and 153 in Zanno et al., 2009). As discussed above, Norell et al.'s original character formulation was problematic. Both Senter and Zanno et al. separated the scoring of ungual I from that of unguals II and III, and we have further separated the scorings of unguals II and III into separate characters (167 and 168).

168. Manual ungual III - shape of proximodorsal margin - smoothly convex (0); with lip rising dorsally (1) (154 in Norell et al., 2001; refined by 300 in Senter, 2007 and 153 in Zanno et al., 2009). The discussion for character 167 applies.

169. Ilium - ventral expansion of preacetabular process - expansion (measured as distance preacetabular tip extends ventrally past highest point at ventral edge of preacetabular process / height of preacetabular process at that point) >18% (0); <19% but present (1); absent (2) (ordered) (155 in Norell et al., 2001; 39 in Xu, 2002). It quantifies the difference between "gently curved" and "hooked" plus "very strongly hooked" in Norell et al.'s original. Given this method of measurement, therizinosauroids no longer fall into a separate state as they do in Norell et al.'s matrix. Xu added the correlated character of an anteroventral process being present or absent.

170. Ilium - length of preacetabular process, measured from notch below process - <50% of postacetabular length, measured from posterior edge of ischial peduncle base (0); 50-120% (1); >120% (2) (ordered) (156 in Norell et al., 2001; states 0 and 1 separated after 155 in Turner et al., 2012). The posterior margin of the ischial peduncle is defined as the point where the posteroventral edge is more horizontal than vertical. Norell et al.'s original character scored two subtly different ratios, a preacetabular process "markedly longer" than the postacetabular process, and a preacetabular process "more than two-thirds of total ilium length." We have quantified the character based on the former ratio. Turner et al. added the state "postacetabular blade much longer", which we've also quantified and correctly ordered.

171. Ilium - angle of anterior edge of preacetabular process - less than 11 degrees posteroventrally (0); 10-70 degrees posteroventrally (1); more than 70 degrees posteroventrally, often being the anteroventral edge of a round process (2) (ordered) (157 in Norell et al., 2001; 269 in Xu et al., 2009; 289 in Senter, 2011). Our wording attempts to formulate Norell et al.'s states (anterior end of ilium "gently rounded or straight", "strongly curved" or "pointed at anterodorsal corner") as consequences of a single variable and quantify the differences. Xu et al. and Senter independently added characters scoring for the anteroposterior position of the anteroventral preacetabular corner, which is just another way to state this variable.

172. Ilium - dorsal margin of acetabulum in dorsal/ventral view - markedly convex due to well developed supracetabular crest (0); slightly convex due to poorly developed supracetabular crest (1); straight or concave due to absence of supracetabular crest (2) (ordered) (158 in Norell et al., 2001).

173. Ilium - lateral surface at dorsal edge posterodorsal to acetabulum - flat (0); developed into supratrochanteric process (1) (159 in Norell et al., 2001).

174. Ilium - shape of postacetabular process - quadrangular posteriorly (0); triangular posteriorly (1) (160 in Norell et al., 2001).

175. Ilium - dorsal postacetabular edge angled posterolaterally - <11 degrees between right and left ilia (0); >10 degrees (1) (161 in Norell et al., 2001). This quantifies Norell et al.'s character contrasting "parallel" postacetabular processes and those that "diverge posteriorly." Brusatte et al. (2014) argue in their commentary that taxa with contacting dorsal ilial edges (character 469:0) such as *Garudimimus* and *Gallimimus* should be scored as state 0 because their divergent posacetabular process are "a result of the reorientation of the lateral surface to face dorsolaterally, due to the medial orientation of the two blades to contact each other (or nearly contact) above the acetabulum." However, contrary to their statement "without this medial rotation the postacetabular processes would be parallel or nearly parallel to each other", the processes diverge even when they are in this oblique view down their long axis (e.g. left ilium of *Gallimimus* in Osmolska et al., 1972: plate XLV Fig. 2a).

176. Ilium - depth of brevis fossa - deep (0); shallow (1); absent (2) (ordered) (162 in Norell et al., 2001; states 1 and 2 separated after 161 in Zanno et al., 2009). Norell et al.'s original contrasted "shelf-like" and "deeply concave" ventral surfaces, which Zanno et al. added a new "strongly reduced" state to.

177. Ilium - lateral projection of antitrochanter - undifferentiated from supracetabular area and postacetabular process (0); projected further laterally (1) (163 in Norell et al., 2001). Note this refers only to laterally projecting processes, not the distinct but unprojected articular surface described as an antitrochanter in basal theropods. It differs slightly from Norell et al.'s version which grouped "poorly developed" and absent antitrochanters together.

178. Ilium - posterior extent of m. cuppedicus fossa over pubic peduncle in lateral view - over at least half of peduncle (0); over less than half (1) (164 in Norell et al., 2001). This quantifies Norell et al.'s character.

179. Ilium - ventral surface on preacetabular process for m. puboischiofemoralis internus (iliofemoralis internus and iliotrochantericus cranialis in Aves) - forms cuppedicus fossa which is overhung laterally (0); forms flat surface or laterally angled fossa which is not overhung laterally (1); muscles attach to undifferentiated blade of ilium (2) (ordered) (165 in Norell et al., 2001; 411 in Turner et al., 2012; 835 in Brusatte et al., 2014). Note Turner et al. added Clarke's bird matrix character scoring for cuppedicus fossa absence, weighting this trait in their analysis. Brusatte et al.'s character 835, scoring for "pronounced horizontal shelf continuing from anterior margin of pubic peduncle to demarcate the cuppedicus fossa dorsally" is a necessary correlate of state 0.

180. Ischium - proximodorsal edge - unprojected (0); projected into low process separated from ilial peduncle by shallow concavity (1); projected into tall process and separated from ilial peduncle by notch (2) (ordered) (166 in Norell et al., 2001; states 1 and 2 separated after 230 in Makovicky et al., 2005). This eliminates the composite variables of "tab-like or pointed" (state 1) and "proximodorsally hooked" from Makovicky et al.'s new character.

181. Ischium - distal curvature - curved dorsally (0); straight (1); curved ventrally (2) (ordered) (167 in Norell et al., 2001; states 0 and 1 separated after 167 in Makovicky et al., 2005 and 166 in Senter, 2007). In taxa with an obturator process, this is based on the curvature distal to the process. In taxa without the process, it is based on the total curvature of the element. Norell et al.'s original character included extra states scoring for twisting of the ischial shaft and lateral convexity, while we have followed Makovicky et al. in limiting states to curvature in side view and adding a state for ventral curvature. Senter used Makovicky et al.'s formulation for this and the following character.

182. Ischium - dorsoventral convexity of lateral surface - concave (0); flat or convexly rounded (1); ridged (2) (ordered) (167 in Norell et al., 2001; state 2 after 168 in Makovicky et al., 2005 and 330 in Senter, 2007). This does not include the commonly found concavity between the obturator process and ischial body. While Norell et al. included a state for "laterally concave curvature in anterior view" in their original character scoring for curvature in multiple dimensions, Makovicky et al. separate and reinterpret it as dorsoventral concavity and add a state for ridged ischia. Senter (2011) adds another state to this character (his 318), "with longitudinal groove", to describe the morphology of *Sinornithosaurus* and *Microraptor*. However, this groove is a consequence of the dorsal margin of their longitudinal ridge. Including it as a separate state a priori assumes non-homology of their ridge with e.g. *Buitreraptor*, though dorsoventral placement or dorsal emargination might be coded for in new characters.

183. Ischium - proximodistal position of obturator process apex - <34% down shaft (0); 34-66% (1); >66% (2) (ordered) (168 in Norell et al., 2001; 306 in Senter, 2007). For taxa which have an obturator plate undifferentiated proximally (e.g. *Monolophosaurus*), the distal extent of this plate is measured. This quantifies Norell et al.'s original character and deletes obturator process presence which was already scored by their character 168. TWiG matrices continued scoring for obturator process presence twice through at least Brusatte et al. (2014; their 166 and 168). Senter created a correlated character scoring for an obturator process that reaches the ischial tip, while also stating (p. 19) this was a consequence of the interaction of his characters 167 and 171. Unlike Senter, we view obturator process position purely in relation to its placement on the ischium and not with respect to the ischiopubic ratio. Foth et al. (2014; character 161) reduced the informativeness of this character by combining middle and distal placement into a single state. Brusatte et al. use a slightly different quantification of 30-50% for state 1.

184. Ischium - contact between obturator process and pubis - absent (0); present (1) (169 in Norell et al., 2001; 255 in Zanno et al., 2009). Though Zanno et al. (2009) add a state for supposed incomplete contact in *Nothronychus mckinleyi* and *N? graffami*, the only preserved ischium of the former and left ischium of the latter show a contact surface over most of the distal obturator process.

185. Ischium - development of ischial apron - notch proximally between obturator process and pubic peduncle (0); notch enclosed fully forming obturator foramen (1); no ventral projection or foramen in ischium ventral margin (2) (ordered) (168 in Norell et al., 2001; 324 in Senter, 2011).

186. Ischium - ventral edge of pubic peduncle - flat (0); projected as obturator tuber (1) (172 in Norell et al., 2001). Contra Turner (2008), this is not the same as the more distally located obturator process. The obturator process is for the M. adductor femoris 1 and M. pubo-ischio-femoralis externus according to Hutchinson (2001a), while the obturator tuber is for the ligamentum ischiopubicum.

187. Ischium - length compared to pubis - >100% (0); 66-100% (1); <66% (2) (ordered) (173 in Norell et al., 2001; states 0 and 1 separated after 362 in Zhang et al., 2008; 174 in Senter, 2010). Senter (2010) added character 174 for the short pubofemoral ratio of scansoriopterygids, which has the same purpose as Zhang et al.'s character 362 coding for a long ischiopubic ratio. Foth et al. (2014; character 165) instead used 70% and 50% as cut-offs for states.

188. Ischium - form of median contact - fused (0); unfused but contacting (1); no contact (2) (ordered) (partly unknown as 0/1 in juvenile non-ornithothoracines, where state 1 may transform into state 0 in adults) (174 in Norell et al., 2001). This is slightly different from Norell et al.'s original which had state 1 as "approach one another but do not form symphysis" and state 2 as "widely separated", as contact is much more easy to determine than distance of separation in life.

189. Ischium - dorsoventral expansion of distal end - wider than shaft (0); narrower or equal to shaft (1) (175 in Norell et al., 2001; 363 in Zhang et al., 2008). Zhang et al. added the correlated character of ischia being "distally narrow" versus "distally wide."

190. Pubis - orientation of proximal half in lateral view - <67 degrees from anteriorly horizontal (0); 67-120 degrees (1); 121-150 degrees (2); >150 degrees (3) (ordered) (176 in Norell et al., 2001; states 2 and 3 separated after 198 in Xu, 2002 and 175 in Hwang et al., 2004). This quantifies Norell et al.'s original states, as well as the new states introduced by Xu and Hwang et al. for the condition in Aves.

191. Pubis - anteroposterior length of anterior boot, measured as flare from prior curvature of pubis - >3% of pubic length (0); <4% (1) (177 in Norell et al., 2001; 745 in Brusatte et al., 2014). This length is based on extending the anterior pubic edge distally through any anterior boot, and measuring from the distal extent of that line to the anterior edge of the distal pubis. Both this and the next character are measured parallel to the boot's ventral edge. It quantifies Norell et al.'s original character scoring for "little or no anterior process" or "no anteroposterior projections" and separates it from posterior boot length, scored for character 192 below.

192. Pubis - anteroposterior length of posterior boot, measured as distal depth of pubis excluding anterior boot - >29% of pubic length (0); 15-29% (1); 6-14% (2); <6% (3) (ordered) (177 in Norell et al., 2001; states 2 and 3 separated after 275 in Xu et al., 2009 and 169 in Dal Sasso and Maganuco, 2011; states 0 and 1 separated after 169 in Senter, 2007 and 169 in Dal Sasso and Maganuco, 2011). The posterior boot is measured from the distal extent of the line formed by the prior character to the posterior edge of the distal pubis. It quantifies Xu et al.'s discontinuous states of "about 30% or longer (0) about 20% or shorter (1) than the pubic length" for the entire boot in their new correlated character, Senter's discontinuous states of ?30% and ?40% for entire boot length, and Dal Sasso and Maganuco's ordered character for posterior boot length having cut-off points at present, a fifth and a third of pubic length. Senter (2011) added another state to his version of these last two characters (his 313) coding for the gradually expanded distal pubis of microraptorians. In doing so however, their lack of an anterior boot and presence of a distal expansion are a priori considered nonhomologous with other taxa. This morphology is here analyzed as character 699.

193. Pubis - anteroposterior position of apron - in middle or at posterior edge of shaft (0); at anterior edge of shaft (1) (178 in Norell et al., 2001). This excludes Norell et al.'s composite qualifiers of "cylindrical" versus "anteroposteriorly flattened" pubic shafts for states 0 and 1. Turner et al. (2012) added a state for the supposedly absent aprons in ornithothoracines, but while narrower than most other theropods, taxa like *Sinornis* (Sereno et al., 2002) and *Apsaravis* (Clarke and Norell, 2002) still retain a sharp medial edge. This state is thus rejected. While Zanno et al. (2009) divided state 0 and uniquely coded therizinosauroids as having a posteriorly placed apron, this is also true in at least some of the taxa they coded as having an apron in the middle of the shaft (e.g. *Allosaurus*, *Tyrannosaurus*, *Patagonykus*), so the states are not separated here.

194. Pubis - curvature in lateral view - anteriorly concave (0); straight or sigmoid (1); posteriorly concave (2) (ordered) (179 in Norell et al., 2001; states 1 and 2 separated after 179 in Calvo et al., 2004, 201 in Xu, 2002, 180 in Makovicky et al., 2005, 308 in Senter, 2007 and 306 in Senter, 2011). Numerous authors corrected Norell et al.'s original character that lacked a way to score taxa with posteriorly curved pubes. While Senter (2011) distinguishes posteriorly curved and posteriorly kinked states, nothing is coded as having posteriorly curved pubes in his matrix, with all taxa actually having this condition being miscoded as having straight pubes instead. Even if it were possible to objectively distinguish these states, a separate character would be necessary to allow the pubic curvature character to remain ordered and not assume a priori that curving is nonhomologous to kinking as Senter's matrix does.

195. Pubis - proximal extent of symphysis, measured to median edge - >41% of pubic length (0); <42% but present (1); absent (2) (ordered) (180 in Norell et al., 2001; 274 in Xu et al., 2009; state 2 after 413 in Turner et al., 2012 and 178 and 408 in Brusatte et al., 2014). This quantifies Norell et al.'s discontinuous states of "about half of pubic shaft length" versus "less than one-third." Contra Xu et al. (2009), there is no consistent difference between the symphyseal lengths of tyrannosauroids, carnosaurs and *Achillobator* compared to other taxa, so their extra state is not included. Brusatte et al. left Turner et al.'s character scoring for no contact (their 408) but also added a state to the current character (their 178) scoring for this, weighting it in their matrix.

196. Femur - medial surface of head - flat to convex (0); concave to form capital ligament fossa (1) (181 in Norell et al., 2001). This eliminates Norell et al.'s original composite qualifiers of a circular shape and position in the center of the medial surface.

197. Femur - separation of anterior and greater trochanters - depth of the intertrochanteric notch (from the tip of the anterior trochanter) >65% the minimum anteroposterior diameter of the femur (0); <66% but still present (1); absent, trochanters fused forming trochanteric crest (2) (ordered) (182 in Norell et al., 2001; 325 in Zanno et al., 2009). This quantifies Norell et al.'s original states 0 and 1 scoring for a "deep cleft" compared to a "small groove." Zanno et al. added a state for fused trochanters to their new character 325, weighting the state since it was already present in their character 184.

198. Femur - section of anterior trochanter in proximal view - anteroposteriorly elongate, forming aliform trochanter (0); equal to transversely elongate (1) (183 in Norell et al., 2001). Note this is often inapplicable for taxa with fused trochanters, but can still be scored if indications of the location of fusion are present.

199. Femur - posterolateral surface at proximal end - flat (0); posterior trochanter for insertion of m. ischiotrochantericus (m. ischiofemoralis in Aves) present but separate from more anterior m. iliofemoralis externus insertion (trochanteric shelf homolog) (1); posterior trochanter present and congruent with trochanteric shelf (2) (ordered) (184 in Norell et al., 2001; states 1 and 2 separated after 414 in Turner et al., 2012). As Hutchinson (2001b) determined, the apparently hypertrophied posterior trochanter of enantiornithines is actually due to their posterior trochanter connecting to their trochanteric shelf. Turner et al. scored for a posterior trochanter twice, first as character 186 then as 414.

200. Femur - posterior surface of midshaft - with convexity for insertion of M. caudofemoralis longus, forming fourth trochanter (0); flat or depressed (1) (185 in Norell et al., 2001).

201. Femur - convexity of anterior edge of anterior trochanter in lateral view - convex or straight (0); concave, forming accessory trochanter distal to it (1) (186 in Norell et al., 2001).

202. Femur - anteromedial surface of distal end - uniformly convex transversely (0); with proximodistal ridge forming medial epicondyle (1) (187 in Norell et al., 2001). Note this is not as restrictive as Brusatte et al.'s (2014) version (their character 186) which must be visible in posterior view.

203. Femur - distal enclosure of popliteal fossa between distal condyles - distally open (0); distally closed (1) (188 in Norell et al., 2001).

204. Fibula - contact with proximal tarsals - contact present (0); contact absent (1) (189 in Norell et al., 2001). This eliminates the composite variable of "tapering distally" that Norell et al. connected to state 1.

205. Fibula - concavity of proximomedial surface - flat to convex (0); shallow concavity (1); deep concavity (2) (ordered) (190 and 191 in Norell et al., 2001). This combines the correlated characters of Norell et al. scoring for proximodistal concavity (190) and a deep fossa (191) on the medial surface, and eliminates the composite variable of the fossa's shape being oval.

206. Proximal tarsals - depth of concavity between medial and lateral condyles in distal view - <20% of anteroposterior depth of deepest condyle (0); >19% (1) (192 in Norell et al., 2001). This quantifies the prominence of the tendinal groove in Norell et al.'s character.

207. Tibia - anteromedial surface of proximal portion - flat (0); with proximodistal ridge forming medial cnemial crest (1) (193 in Norell et al., 2001).

208. Astragalus - height of ascending process, from distal tibiotarsal edge to ascending process apex - >84% of astragalocalcanear width (0); <85% (1) (194 in Norell et al., 2001). We separate the composite variables of ascending process height, width and lateral concavity. This quantifies tall versus short processes in Norell et al.'s character, although note as in their character "short" is state 1, which is counterintuitive given it is plesiomorphic for dinosaurs.

209. Astragalus - mediolateral width of ascending process - measured halfway up, <58% width of astragalocalcaneum (0); >57% (1) (194 in Norell et al., 2001; refined by 215 in Xu, 2002). As noted for character 208, this is separating ascending process width from Norell et al.'s composite character. In addition, it quantifies the width. Xu added a state to Norell et al.'s which merely specified a narrow process (and thus potentially overlapped his state 2) but probably intended to imply a short process as well.

210. Astragalus - concavity of medial edge of ascending process - convex to slightly concave (0); markedly concave, at least comparable in depth to *Shuvuuia* and *Mononykus* (1) (194 in Norell et al., 2001). As noted for character 208, this separates ascending process lateral concavity from other states in Norell et al.'s composite character. While not yet quantified, this codes for a depth at least similar to that in parvicursorines (e.g. Fig. 4a2 in Chiappe et al., 1998).

211. Astragalus - confluence of condyles and ascending process - confluent (0); separated by groove (1) (195 in Norell et al., 2001). This separates Norell et al.'s original composite character in which state 1 scored for a groove or a fossa (the latter covered by character 212 below).

212. Astragalus - convexity of anterior surface at base of ascending process - flat compared to rest of ascending process (0); distinctly depressed as a fossa (1) (195 in Norell et al., 2001; 298 in Makovicky et al., 2012; 776 in Brusatte et al., 2014). Makovicky et al. add a second state for lateral displacement of the fossa in some alvarezsaurids, but contra their coding, the fossa does not seem to be more laterally positioned in e.g. *Patagonykus* and *Alnashetri* compared to e.g. *Garudimimus* or *Caudipteryx*. The state is thus unused here.

213. Astragalus - form of contact with tibia - unfused (0); fused (1) (unknown in juveniles) (196 in Norell et al., 2001). This separates Norell et al.'s original composite character scoring for both tibiotarsal and astragalocalcanear fusion (the latter covered by character 214 below).

214. Astragalus - form of contact with calcaneum - unfused (0); fused (1) (unknown in juveniles) (196 in Norell et al., 2001; refined after 198 in Turner et al., 2012).

215. Distal tarsals - form of contact with metatarsals - unfused (0); fused (1) (unknown in juveniles) (197 in Norell et al., 2001). This eliminates the composite variables of an intercondylar prominence (partly scored by character 668 here) and the timing of tarsometatarsal fusion.

216. Metatarsals II-IV - extent of fusion - unfused (0); at least two fused proximally, but not distally (1); fused proximally and distally, but not distal to distal vascular foramen between III and IV (2); fusion encloses distal vascular foramen (3) (ordered) (unknown in juveniles) (198 in Norell et al., 2001; states 2 and 3 separated after 200 in Turner et al., 2012). Note this avoids the developmental assumption of Norell et al.'s state 1- "co-ossification of metatarsals begins proximally."

217. Metatarsal II - depth of distal articular groove - absent or shallow (0); deep (1) (199 in Norell et al., 2001).

218. Metatarsal III - depth of distal articular groove - absent or shallow (0); deep (1) (200 in Norell et al., 2001). Agnolin and Novas (2011) separate absent and shallow states, but contra their statements and coding, most included taxa have a shallow groove. Thus the states are not differentiated here.

219. Metatarsal III - proximal transverse constriction - anterior exposure wider or subequal to metatarsals II and IV (0); anterior exposure significantly narrower than II and IV but still present at proximal end, subarctometatarsal (1); hidden anteriorly at proximal end by contact between II and IV but still exposed on proximal surface, arctometatarsal (2); does not extend to proximal surface, hyperarctometatarsal (3) (ordered) (201 in Norell et al., 2001; states 0 and 1 separated after 222 in Xu, 2002, 200 in Novas and Pol, 2005 and 203 in Makovicky et al., 2005; 358 in Senter, 2007; 369 in Senter, 2011; 428 in Turner et al., 2012). Note numerous authors independently added a state for subarctometatarsaly to Norell et al.'s original. Though usually coded as a separate character, the condition in derived ornithuromorphs generally described as 'proximal end of metatarsal III displaced plantarily' (as in 428 of Turner et al., 2012) is equivalent to the subarctometatarsal condition of e.g. *Sinovenator* where proximal exposure of metatarsal III is greater posteriorly than anteriorly.

220. Metatarsal IV - transverse width at midshaft - <117% of depth at midshaft (0); >116% of depth (1) (202 in Norell et al., 2001; 313 in Senter, 2007). This quantifies Norell et al.'s original description of "mediolaterally widened and flat" for state 1. Senter created a character scoring for mediolateral compression in all metatarsals, which is partially correlated with this. We have divided his composite character, and score metatarsal II proportions as character 386.

221. Metatarsal I - presence - present (0); absent (1) (203 in Norell et al., 2001; 360 in Senter, 2011; 447 in Turner et al., 2012; 742 in Brusatte et al., 2014). We follow Senter and Turner et al. in separating this from the positional states in Norell et al.'s original composite character, as absence is not a position. Brusatte et al. used two characters for this condition, their 442 and 742, weighting it compared to other features.

222. Metatarsal I - proximodistal position - distance between distal ends of metatarsals I and II when articulated >30% length of metatarsal II (0); 18-30% (1); 6-18% (2); <6% (3) (ordered) (203 in Norell et al., 2001; 45 in Xu, 2002; states 2 and 3 separated after 446 in Turner et al., 2012). This eliminates reference to posteromedial position in Norell et al.'s original composite character (which is very difficult to determine even in very well preserved fossils). It also quantifies the original's states "near its proximal end", "in the middle" and "distal quarter." Turner et al. proposed a new character for metatarsal I "inline distally with others" which is correlated with this in their matrix, thus we quantify it and add it here as an additional state.

223. Metatarsal I - proximal extent - ends distal to tarsus (0); extends to contact tarsus (1) (204 in Norell et al., 2001). This eliminates the composite variable of proximal tapering found in Norell et al.'s original. Senter (2007) eliminated the variable of proximal articulation instead (his character 203), and reworded this for his character 362 in 2011.

224. Pedal ungual II - length on straight line compared to ungual III - <141% (0); >140% (1) (205 in Norell et al., 2001; refined after 201 in Senter, 2007). This separates the variables of length, curvature (now character 225) and hyperextension (rejected, see below) from Norell et al.'s original, and quantifies the length. Senter (2007) also separated length as a separate character. Senter (2011) adds a third state to this character (his 388), comparing enlarged ungual II size with phalanx II-1 length. However the latter is an independent variable, and having such an unordered character results in the condition in taxa with the new state (four troodontids in his matrix) a priori assumed to be non-homologous to the enlargement seen in other paravians.

225. Pedal ungual II - curvature - depth of the dorsal arc <30% of ungual length (measured in a straight line from the proximodorsal tip to the distal tip) (0); >29% (1) (205 in Norell et al., 2001). This separates curvature from other composite variables in Norell et al.'s original, and quantifies the character.

226. Metatarsal IV - transverse width at midshaft compared to metatarsal II - <66% (0); 66-166% (1); >166% (2) (ordered) (206 in Xu et al., 2002a; 869 in Pei, 2015; states 0 and 1 separated after 434 in Turner et al., 2012 and 205 in Dal Sasso and Maganuco, 2011). State 2 quantifies Xu et al.'s original "slender MTII and very robust MT IV", while state 0 for a much narrower metatarsal IV was added after Dal Sasso and Maganuco and part of Turner et al.'s composite character 434, and quantified. While Pei (2015) uses >200% as his quantification, the taxa scored for the state are the same.

227. Skull - depth of snout - height of preorbital snout one fourth from tip >39% of orbit+jugal height (0); <40% (1) (1 in Xu, 2002; 233 in Senter, 2007). This gives a more neutral quantification to where snout depth is measured than Xu's "at the posterior margin of the external naris" or Senter's "at middle of naris" which would vary based on narial position and size (characters 2 and 233 here).

228. Premaxilla - angle between anterior subnarial and ventral margins - >34 degrees (0); <35 degrees (1) (2 in Xu, 2002; 235 in Senter, 2007; 8 in Senter, 2011). This newly quantifies the character, although Xu did provide a graph and commentary on quantification.

229. Premaxilla - subnarial height compared to alveolar length - >180% (0); 101-180% (1); 50-100% (2); <50% (3) (ordered) (state 3 refined after 3 in Xu, 2002 and 260 in Turner et al., 2012; state 0 refined after 21 in Gohlich and Chiappe, 2006; states 1 and 2 separated after 237 in Senter, 2007). Length is measured parallel to the ventral premaxillary margin and height perpendicular to that. Height is from the naris' ventral margin to the alveolar margin of the snout, even if the maxilla underlies that part of the naris. State 0 is quantified from Gohlich and Chiappe's "very high, much shorter rostrocaudally than dorsoventrally" and Turner et al.'s "significantly higher than wide" states. Brusatte et al. (2014) use 100% and 200% as cut-off points in their equivalent character 256.

230. Premaxilla and maxilla - space between toothrows of each element - similar to that between teeth in each element (0); greater, forming diastema between elements (1) (4 in Xu, 2002).

231. Maxilla- shape of promaxillary fenestra - minimum diameter >35% of maximum diameter (0); <36% (1) (6 in Xu, 2002). This quantifies Xu's description of "slit-like."

232. Nasal - convexity of dorsal edge in lateral view - convex or straight (0); concave (1) (7 in Xu, 2002; 245 in Senter, 2007). Note Xu's formulation lacks a state to score taxa with convex nasals. Senter (2010) rejected this character, claiming that Norell et al. (2006) found it to be a taphonomic artifact, but the latter merely said the shape was exaggerated by transverse crushing, not completely caused by it.

233. External naris - length - longest axis <49% of orbit+jugal height (0); 49-80% (1); >80% (2) (ordered) (8 in Xu, 2002; 274 in Turner et al., 2012; 237 in Senter, 2010; 270 in Brusatte et al., 2014). Xu quantified this, but compared it to skull length, while Turner et al. and Brusatte et al. used Clarke's character comparing it to antorbital fossa length and Senter compared it to orbit length. Here we have quantified Xu's three states compared to orbit+jugal height.

234. Postorbital - length of posterior process - shorter or equal to ventral process (0); longer (1) (9 in Xu, 2002). Both processes measured from opposite edge of element. Xu's original character formulation lacked a state for taxa with longer posterior processes, only contrasting "shorter" with "subequal."

235. Frontal - dorsal surface of supratemporal fossa - flat (0); with pit (1) (10 in Xu, 2002; 466 in Turner et al., 2012).

236. Parietal - minimum width - less or equal to minimum frontal transverse width (0); more (1) (11 in Xu, 2002).

237. Parietal - dorsal outline - concave to straight (0); convex (1) (12 in Xu, 2002; 584 in Brusatte et al., 2014).

238. Quadrate - proximodistal position of pterygoid process apex - >45% (0); <46% (1) (13 in Xu, 2002). Measured as percentage of element from ventral edge, along line drawn between posterior edges of quadrate head and condyles. It quantifies Xu's original character scoring for a "ventrally located" apex.

239. Basioccipital - shape of notch between basal tubera - rounded (0); angled (1) (14 in Xu, 2002; 222 in Hwang et al., 2004). Hwang et al.'s character also includes the composite variable of distance between basal tubera, here scored as character 294.

240. Laterosphenoid - lateral surface dorsal to exit for oculomotor (III) nerve - flat to convex (0); excavated by fossa (1) (15 in Xu, 2002; 221 in Hwang et al., 2004).

241. Dentary - posterior depth compared to length - <15% (0); 15-50% (1); >50% (2) (ordered) (16 in Xu, 2002; 343 in Senter, 2007; 70 in Zanno et al., 2009; 621 in Brusatte et al., 2014). Xu quantifies this in his discussion, which we largely follow except use a value of <15% for state 0 instead of <8% to better match his taxon scores.

242. Teeth - DSDI - <1.19 (0); >1.2 (1) (18 in Xu, 2002; 247 in Turner et al., 2007a; 258 in Senter, 2007). DSDI is the number of mesial serrations per unit divided by the number of distal serrations per that unit. We quantify "significantly" and "markedly" smaller mesial serrations as used by prior authors, although Xu's discussion did indicate a cutt-off point between 1 and 1.35 between states.

243. Dorsal vertebrae - length of column - >119% of femoral length (0); <120% (1) (19 in Xu, 2002). This is measured from the acetabulum to the anterior edge of the first dorsal centrum so covers the anterior sacrum as well. Xu used a hindlimb/trunk ratio, which we change to femur/trunk since we have other characters comparing tibiotarsal and metatarsal length to femoral length (characters 383 and 385).

244. Dorsal vertebrae - centrum proportions of dorsals 6-13 - length <83% of posterior height (0); 83-130% (1); 130-200% (2); >200% (3) (ordered) (667 in Brusatte et al., 2014; states 1 and 2 separated after 20 in Xu, 2002; states 0 and 3 after 264 in Senter, 2007; state 3 after 171 in Senter, 2011). Our states 1 and 2 quantify Xu's "short" and "long" states based on his taxon coding, states 0 and 1 use Senter's discontinuous quantification ("?1.2×taller than long (0) or height?length (1)"), and state 3 uses Senter's quantification for his new state. We also specify posterior dorsals as in Senter (2011), but not Xu or Senter (2007). Brusatte et al. specify mid to posterior dorsals and use a ratio of 100%.

245. Dorsal vertebrae - anteroposterior expansion of neural spine apex in dorsals 6-13 - <116% of minimum anteroposterior spine length (0); >115% (1) (21 in Xu, 2002; 206 in Hwang et al., 2004). This quantifies the "fan-shaped" description given by prior authors for state 1. Senter (2011) added a third state to this character (his 176), "shaped like partial fan, with posterior corner of fan present but anterior corner absent", only scored as present in *Scipionyx*. Here these are considered to be posteriorly sloped neural spines as opposed to a different state of distal expansion.

246. Sacral vertebrae - transverse width of ancestral neotheropod sacral centra 1 and 2 - >69% of ancestral sacral centrum 3 width (0); <70% (1) (22 in Xu, 2002). Width measured is minimum width at constriction of centrum. This quantifies Xu's character scoring for "significantly larger" middle sacral centra.

247. Caudal vertebrae - distal anteroposterior width of transverse processes on caudals 1-4 - tapered (0); subequal (1); expanded (2) (ordered) (23 in Xu, 2002; 237 in Xu et al., 2009; 373 in Xu et al., 2011a). This eliminates the composite reference to cross section in Xu's and Xu et al.'s (2009) character, which describes our states 0-2 as "rod-like", "strap-like" and "plate-like" respectively. Xu et al.'s (2011a) later version only distinguished tapered processes from all others.

248. Caudal vertebrae - length of caudals 10-15 - shorter than caudal 1 (0); 100-200% longer (1); >200% longer (2) (ordered) (24 in Xu, 2002; 193 in Senter, 2011; states 1 and 2 separated after 336 in Senter, 2007; 439 in Turner et al., 2012; 238 in Xu et al., 2009; states 0 and 1 separated after 280 in Zanno et al., 2009). This follows Xu's states for quantification. While he did not explicitly quantify state 2, his commentary states it would be between 1.6 and 2.2. Senter (2007) explicitly uses two times longer, and in 2011 quantifies a similar character to ours except that his new equivalent to our state 1 is 130-200%. Xu et al. only use "significantly longer than" for state 2, and Turner et al. use a discontinuous character not scoring taxa with caudals between 2 and 3 times or over 4 times dorsal vertebral length. Zanno et al. uniquely compare centrum length to centrum width, which approximates our state 0.

249. Sternal ribs - number which contact dorsal ribs - none to three (0); at least four (1) (26 in Xu, 2002). As some taxa have sternal ribs which contact dorsal ribs but not the sternum, this is equal to the number of dorsal ribs with distal expansions in those taxa with unossified sternal ribs, and the number of ossified sternal ribs in taxa with ossified sternal ribs.

250. Furcula - size - length of arm from omal tip to base excluding hypocleidium <36% of scapular length (0); >35% (1) (27 in Xu, 2002). This quantifies Xu's "large" furcula character.

251. Coracoid - surface just distal to coracoid tuber - solid (0); pierced by supracoracoid fenestra (1) (28 in Xu, 2002; 440 in Turner et al., 2012; 249 in Xu et al., 2009).

252. Scapula - contact between acromion and coracoid - present to near anterior tip (0); absent, scapula-coracoid contact limited to short area near glenoid (1) (29 in Xu, 2002; 269 in Senter, 2007; 240 in Xu et al., 2009; 214 in Senter, 2011). Contra Xu et al. (2009), maniraptorans do not have thinner acromial articulations than other theropods and thus do not warrant an intermediate state. Senter (2011) joined this state with his character scoring for acromion shape to form a composite character.

253. Scapulocoracoid - length of glenoid edge formed by scapula, with glenoid edge of each bone simplified to a straight line - <160% the length of glenoid edge formed by coracoid (0); >159% (1) (30 in Xu, 2002; 839 in Brusatte et al., 2014). It quantifies Xu's state of glenoid formed "mainly by scapula" and Brusatte et al.'s state "scapula contribution markedly anteroposteriorly longer."

254. Humerus - length compared to femur - <38% (0); 38-69% (1); 70-100% (2); >100% (3) (ordered) (31 in Xu, 2002; state 3 separated after 274 in Senter, 2007; 266 in Turner et al., 2012; 251 in Xu et al., 2009). States 0-2 are quantified based on Xu's character scoring forelimb elongation compared to femoral length, using his taxon scores to delimit states. Senter, Turner et al. and Xu et al. each added a state for humeri longer than femoral length. Instead of our states 0-2, Senter used 50% as the dividing point while Turner et al. had a discontinuous character scoring for <~40% and >~45%.

255. Femur - distal extent of lateral condyle past medial condyle - <11% of distal femur width (0); >10% of distal femur width (1) (772 in Brusatte et al., 2014). This excludes the composite variable of lateral condyle shape, rounded versus conical, from Brusatte et al.'s character.

256. Radius - midshaft anteroposterior diameter - >71% of ulnar midshaft anteroposterior diameter (0); 51-71% (1); <51% (2) (ordered) (33 in Xu, 2002; states 1 and 2 separated after 278 in Senter, 2007 and 443 in Turner et al., 2012).

257. Manual phalanx I-1 - length compared to metacarpal II - <70% (0); 70-100% (1); >100% (2) (ordered) (34 in Xu, 2002; 284 in Senter, 2007; 739 in Brusatte et al., 2014). The states are based on Xu's quantified but discontinuous character comparing the length of metacarpal I plus phalanx I-1 to metacarpal II, using his taxon scores. Senter and Brusatte et al. used a character distinguishing or state 2 from other conditions. Note that this and other phalangeal length characters include the posterior lips in the total length of the phalanx, unless otherwise specified.

258. Manual phalanx III-2 - length compared to III-1 - >105% (0); 60-105% (1); <60% (2) (ordered) (35 in Xu, 2002; 294 in Senter, 2007; 268 in Xu et al., 2009). This quantifies Xu's and Xu et al.'s states of "significantly longer" and "significantly shorter." Senter used a similar character scoring for <50%. Note that for this and other characters involving phalanges on manual digit III, taxa with a reduced number of phalanges are coded as inapplicable. This is due to the uncertain homology between phalanges (e.g. confuciusornithiforms reduce phalanx III-1, so it's plausible the remaining two phalanges in basal ornithothoracines are homologous to the ancestral III-2 and III-3).

259. Manual phalanx III-2 - proximoventral heel, prominence measured as angle between dorsal and ventral edges of element - <11 degrees (0); >10 degrees (1) (36 in Xu, 2002). Though Xu (2002) and Xu and Zhang (2005) refer to a keel, Xu and Wang (2004b) refer to a heel, which seems to be the correct feature. Note the angle includes the distal condyles as well. It quantifies Xu's qualifier of "prominent" for state 1.

260. Manual phalanx III-3 - dorsoventral depth of shaft just proximal to distal condyles - similar to rest of shaft (0); much shallower than rest of shaft (1) (37 in Xu, 2002).

261. Ilium - length - >63% of femoral length (0); <64% (1) (38 in Xu, 2002; 694 in Brusatte et al., 2014). This specifies Xu's discontinuous character with states "more than 70%" and "less than 60%." Brusatte et al. also had discontinuous states with no state corresponding to 86-94%.

262. Ilium - anteroposterior diameter of external acetabulum (measured between lateral surfaces of peduncles, excluding any medial walls or angled anterior and posterior acetabular surfaces) - >18% of ilial length (0); <19% (1) (40 in Xu, 2002). It quantifies Xu's qualifier of "small" for state 1.

263. Ilium - angle main axis of pubic peduncle projects, compared to line drawn between bases of peduncles - anterior to vertical (0); posterior (1) (41 in Xu, 2002; 310 in Zanno et al., 2009; 806 in Brusatte et al., 2014). Zanno et al.'s and Brusatte et al.'s are composite characters scoring for both peduncle curvature and distal surface orientation, the latter covered by character 472 here.

264. Pubis - lateral surface of mid shaft - smooth (0); with lateral projection (1) (42 in Xu, 2002; 231 in Makovicky et al., 2005; 307 in Senter, 2011).

265. Ischium - dorsal process at midshaft - absent (0); present (1) (43 in Xu, 2002; 232 in Makovicky et al., 2005; 334 in Senter, 2007).

266. Tibia - surface just posterior to fibular crest - flat (0); longitudinally grooved (1) (44 in Xu, 2002).

267. Pedal phalanx II-2 - length compared to II-1 - <60% (0); 60-82% (1); 83-100% (2); >100% (3) (ordered) (46 in Xu, 2002; state 0 separated after 743 in Brusatte et al., 2014; states 0 and 3 separated after 320 in Senter, 2007). States 1 and 2 are based on Xu's taxon scores for his character "pedal phalanx II-1 significantly longer (0) or subequal or shorter than pedal phalanx II-2 (1)" (note we reverse the ratio being measured). States 0 and 3 are based on Senter's quantified states. Brusatte et al. used 60% as the cut-off in their character.

268. Pedal phalanx II-2 - height of distal condyles compared to length (excluding posterior lips) - <61% (0); >60% (1) (47 in Xu, 2002; 321 in Senter, 2007; 378 in Senter, 2011). We follow Senter (2007) in quantifying Xu's character scoring "highly abbreviated" phalanx II-2, although he uses 50% as the cut-off point. Senter (2011) later revised the character to score for much less than 50% versus only slightly less than 50%, which excludes any taxon with a ratio of 50% or more and is subjective.

269. Metatarsal IV - convexity of posterior surface in section - flat or broadly convex (0); sharply keeled (1) (48 in Xu, 2002; 223 in Novas and Pol, 2005; 229 in Makovicky et al., 2005; 333 in Senter, 2007). This excludes the composite variable of medial placement from Xu's original, as did future authors.

270. Pedal phalanx IV-4 - length compared to IV-3 - <101% (0); >100% (1) (49 in Xu, 2002; 255 in Nesbitt et al., 2011).

271. Metatarsal V - length compared to metatarsal IV - <41% (0); >40% (1) (50 in Xu, 2002; 315 in Senter, 2007; 441 in Turner et al., 2012; 436 in Cau et al., 2015). We follow Cau et al. who quantify Xu's "long" state and eliminate the composite variables of "bowed" and "expanded midshaft." Turner et al. also include "bowed" as a composite variable. Senter quantified it as well, but uses a slightly different cut-off value of 50%.

272. Pedal ungual I - depth of the dorsal arc compared to ungual length (measured in a straight line from the proximodorsal tip to the distal tip) - <23% (0); >22% (1) (51 in Xu, 2002; refined after 319 in Senter, 2007). Xu used a composite character scoring for multiple variables of pedal unguals in general (curvature- our characters 272 and 273, cross section- see character 274, and robusticity- rejected). We follow Senter in separating hallux curvature as a character and quantify his "weakly curved" and "strongly curved" states.

273. Pedal unguals III and IV - depth of the dorsal arc compared to ungual length (measured in a straight line from the proximodorsal tip to the distal tip) - <16% (0); 16-21% (1); >21% (2) (ordered) (51 in Xu, 2002; states 0 and 1 separated after 282 in Li et al., 2010 and 746 in Brusatte et al., 2014; refined by 324 in Senter, 2007 and 337 in Zanno et al., 2009). As noted for character 272, Xu's original pedal ungual character was a composite. Here we follow Senter in separating curvature of unguals III and IV (ungual II is covered by character 225 above) and quantify his "straight or weakly curved" versus "strongly curved" states. Zanno et al. followed him in separating this variable as well. Our state 0 is based on Li et al.'s character scoring "straight" versus "curved" pedal unguals, quantified for the first time.

274. Pedal unguals - cross section of III and IV - expanded ventrally, triangular (0); subequal in transverse width dorsally and ventrally (1) (51 in Xu, 2002; 245 in Senter, 2010). This again separates a variable in Xu's originally composite character, which was also done by Senter.

275. Feathers - presence - absent, skin naked or scaled (0); present, unbranched or branched filamentous integument (1) (52 in Xu, 2002; 460 in Turner et al., 2012). This does not include pedal scutes, as seen in e.g. *Concavenator*, NGMC 91 and crown Aves. It is also recognized that some taxa may have had both feathered and extensive unfeathered areas, such as in *Kulindadromeus*, so that taxa coded as lacking feathers may be recoded in the future based on new material.

276. Feathers - arrangement of barbs - unbranched or with basally radiating barbs (0); with barbs arranged in a vane (1) (53 in Xu, 2002; 461 in Turner et al., 2012; 877 in Gianechini et al., 2018).

277. Feathers - length of feathers along metatarsus - absent to comparable to body feathers (0); elongated (1) (54 in Xu, 2002; 560 in Foth et al., 2014; 881 in Gianechini et al., 2018). This specifies metatarsal instead of generic hindlimb feathers, and does not include Xu's, Foth et al.'s or Gianechini et al.'s composite variable of them needing to be vaned.

278. Pubis - angle of posterior pubic boot main axis to distal shaft - >79 degrees (0); <80 degrees (1) (199 in Xu, 2002; 488 in Gianechini et al., 2017). Angle measured at average of center of boot and shaft near boot. Xu made Norell et al.'s original pubic boot character more composite by breaking his 'short anterior but long posterior boot' state into two, one with a hooked posterior boot and one without. Our character separates the hooked morphology and quantifies it. Gianechini et al. added a separate character similar to ours but using 90 degrees as the cut-off point.

279. Chevrons - transverse convexity of posterior ventral edge in distal elements - convex to straight (0); concave to form bifurcated posterior ventral process (1) (123 in Hwang et al., 2004). Note this is equivalent to the ventral edge in taxa which lack anteroventral chevron processes. Hwang et al. made a composite character from Norell's scoring anterior bifurcation by adding a state for chevrons bifurcate at both ends. Each end is a separate character here as they vary independently.

280. Metacarpal I - proportions in dorsal/ventral view - longer than wide (0); wider than long (1) (149 in Hwang et al., 2004; 473 in Brusatte et al., 2014). Hwang et al. added this composite variable as a new state to Norell et al.'s character scoring for metacarpal I length. We have separated it here.

281. Manual phalanx I-1 - minimum transverse width - less than or equal to minimum transverse width of radius (0); greater (1) (207 in Hwang et al., 2004). Note Brusatte et al.'s (2014) state 1 differs in practice, as they state only taxa in which the ratio is "dramatically larger" are scored that way, not others that are only "slightly larger (~10%)."

282. Angular - posterior extent at ventral mandibular edge - extends to reach >94% down mandibular length from symphysis (0); <95% (1) (208 in Hwang et al., 2004). This quantifies Hwang et al.'s character state 1 of "angular suture turns ventrally and meets ventral border of mandible rostral to glenoid."

283. Surangular - dorsolateral surface just anterior to glenoid - unprojected (0); dorsally projected as everted ridge (1) (209 in Hwang et al., 2004). This eliminates the functional qualifier of Hwang et al.'s original- "for articulation with lateral process of lateral quadrate condyle."

284. Metacarpal I - convexity of distal end in dorsal view - deeply concave due to well developed ginglymus (0); slightly concave (1); flat to convex (2) (ordered) (210 in Hwang et al., 2004; states 0 and 1 separated after 213 in Li et al., 2010). Turner et al. (2012) divided metacarpal I morphology into flat and round states to separate the avian and ornithomimid conditions, but most taxa are intermediate so the morphologies are not distinguished here. This separates Hwang et al.'s original character which scored for both metacarpals I and II, and follows Li et al. in scoring only metacarpal I and adding a new state contrasting deep and shallow grooves.

285. Metacarpal II - convexity of distal end in dorsal view - concave (0); flat to convex (1) (210 in Hwang et al., 2004). As noted above, this is a result of separating Hwang et al.'s original composite character.

286. Dentary - convexity of anterodorsal edge - concave to roundly convex (0); with abrupt ventral deflection (1) (212 in Hwang et al., 2004).

287. Quadrate - lateral exposure of head - covered by squamosal (0); visible laterally (1) (213 in Hwang et al., 2004).

288. Ilium - ventral projection of medial brevis ridge - at least partly ventral to lateral brevis fossa edge (0); reduced to not be visible laterally (1) (214 in Hwang et al., 2004). This eliminates the composite qualifier of brevis fossa depth adjacent to the ischial peduncle found in Hwang et al.'s original character.

289. Femur - development of insertion for M. iliofemoralis externus on proximolateral surface - unprojected to low mound (0); forms longitudinal ridge (1); forms trochanteric shelf (2) (unordered) (215 in Hwang et al., 2004; state 2 new to TWiG, originally 40 in Perez-Moreno et al., 1993).

290. Dentary teeth - extent - over most of alveolar edge (0); restricted to anterior tip (1); absent (2) (ordered) (217 in Hwang et al., 2004; state 2 also 85 in Senter, 2007 and 146 in Senter, 2011). Senter (2007) added this state to his character scoring for dentary tooth number, which is an example of ambiguity in how to properly form some morphological characters, as hypothetically either a reduced extent of teeth or reduced number of teeth could lead to edentulousness. In 2011, he added a separate character for a toothless dentary.

291. Coracoid - depth of subglenoid notch - anteroposterior length from anterior coracoid edge to posterior tip of coracoid glenoid (mediolateral length from medial coracoid edge to lateral tip of coracoid glenoid in Aves) <106% of depth from anterior coracoid edge to subglenoid notch (0); >105% (1) (218 in Hwang et al., 2004). This is considered inapplicable in taxa with proximodistally elongated coracoids (character 143, state 2), as the exact axis chosen to be proximodistal heavily influences the outcome and is difficult to assign objectively. It is a quantified version of Hwang et al.'s character scoring for a coracoid "dee[p]ly notched just ventral to glenoid, glenoid lip everted."

292. Articular - curvature of retroarticular process - straight or decurved (0); upcurved (1) (219 in Hwang et al., 2004). Note Hwang et al.'s original character lacks a way to score taxa with decurved or more than "gentle" dorsal curvature.

293. Scapula - lateral surface dorsal to glenoid - flat (0); with dorsoventral ridge forming supraglenoid buttress (1) (220 in Hwang et al., 2004).

294. Basioccipital - transverse width across basal tubera - >164% of occipital condyle transverse width (0); <165% (1) (222 in Hwang et al., 2004). As noted above, Hwang et al.'s original character is a composite also scoring for notch shape between the tubera (character 239 here).

295. Metatarsal II - length compared to metatarsal III - <98% (0); >97% (1) (259 in Xu and Zhang, 2005). This specifies Xu ang Zhang's composite character which also covered metatarsal IV length, and quantifies their "subequal" qualifier for state 1.

296. Basioccipital - posterior surface ventrolateral to occipital condyle - flat (0); excavated by subcondylar recess (1) (223 in Kirkland et al., 2005; 252 in Zanno et al., 2009; 255 in Li et al., 2010; 612 in Brusatte et al., 2014). Zanno et al. divided taxa having subcondylar recesses into two states, one only present in *Tyrannosaurus* where the recesses are "isolated from exits of CN X-XII." Yet *Tyrannosaurus* actually has their state 2 where they share a depression (Brochu, 2003: Fig. 5A), so the states are left undivided here.

297. Humerus - transverse width of entepicondyle - narrow or absent (0); wide, >15% of distal humeral width (1) (225 in Kirkland et al., 2005; 291 and 343 in Zanno et al., 2009; 795 and 797 in Brusatte et al., 2014). Entepicondylar width is measured from the medial edge of the entocondyle. This quantifies previous versions' "large", not "poorly developed" and "located well medial to ulnar condyle" descriptions for state 1. Zanno et al. use their character 343 to score entepicondyle width but also include a state for a "poorly developed, absent" entepicondyle in their character scoring for the compression of the structure. This weights entepicondyle presence in their matrix.

298. Ilium - convexity of ischial peduncle articular surface - concave to flat (0); convex, articulates with socket on ischium (1) (227 in Kirkland et al., 2005; 313 in Zanno et al., 2009; 261 in Li et al., 2010; 748 in Brusatte et al., 2014). This eliminates Zanno et al.'s composite qualifiers of "peg-shaped, ventrally tapering" and Li et al.'s of "peg-like" from state 1.

299. Teeth - labiolingual compression of roots of lateral teeth - labiolingual width <70% FABL (0); >69% (1) (228 in Kirkland et al., 2005). This quantifies Kirkland et al.'s "circular" state 1.

300. Ilium - lateral deflection of preacetabular process - lateral edge of preacetabular process less than 60 degrees from dorsal postacetabular margin (0); 60-90 degrees (1); more than 90 degrees (2) (ordered) (229 in Kirkland et al., 2005; 304 in Zanno et al., 2009; 284 in Li et al., 2010; 802 in Brusatte et al., 2014). This quantifies Kirkland et al.'s "moderately" and "strongly laterally flaring qualifiers for states 1 and 2. Zanno et al., Li et al. and Brusatte et al. all later changed the character to lose information, combining states 1 and 2.

301. Teeth - recurvature - significant (0); slight or absent (1) (230 in Kirkland et al., 2005; 230 in Senter, 2007; 266 in Zanno et al., 2009; 754 in Brusatte et al., 2014). Kirkland et al.'s character only scores for dentary teeth and assumes labiolingual compression for both states, so lacks an ability to score taxa with uncompressed teeth. Senter's revised version is a composite also scoring for labiolingual compression, height versus FABL and basal constriction.

302. Dentary and maxilla - method of tooth implantation - all in distinct sockets (0); some in confluent groove (1) (85 in Makovicky et al., 2005; 260 in Senter, 2007; 155 in Senter, 2011). Note the location of teeth within a groove differs between taxa (e.g. posterior in *Incisivosaurus* vs. anterior in *Urbacodon*), making the state potentially non-homologous. It may thus be divided into multiple characters in future analyses, though the precise condition in most taxa is unreported. While Makovicky et al. and Senter (2007) only specify dentaries, we follow Senter (2011) in also scoring maxillae.

303. Pubis - extent of symphyseal contact - continuous (0); interpubic fenestra (1) (182 in Makovicky et al., 2005; 331 in Senter, 2007). Makovicky et al. also included another state, to describe the morphology in *Unenlagia* and *Archaeopteryx* where there are broad pubic aprons that do not contact until the distal end of the pubes. However, in *Archaeopteryx* ~40% of the pubic apron proximal to the boot remains in contact with the other apron (NHMUK 37001), not qualitatively different from most theropods where a portion of the apron proximal to the symphysis is present as it recedes into the pubic shaft. *Unenlagia*'s holotype (MCF PVPH 78) was reconstructed by Novas and Puerta (1997) as only having a symphysis at the pubic boot, but the pubes were taphonomically splayed apart (cast YPM VP 56517) so that the apron could not maintain contact with its antimere. Whether any of the preserved medial apron edges are natural is unknown. Similarly, the *U. paynemili* holotype (MUCPv-349) lacks its medial edge though Calvo et al. (2004) say it is "apparently ... too narrow to meet at the midline with the opposite element." While it may be possible to design a character that codes for the extent of pubic apron contact proximal to the pubic boot, this will be hindered by the often broken medial edges of disarticulated specimens and difficulty determining where edges contact or where the apron proximally ends in slab specimens. Another interpretation of "no contact" was made by Brusatte et al. (2014), who specified "no contact between pubes distally, pubic apron absent in this part of pubis but present further proximally." This sounds like the condition in coelophysoids where the interpubic fenestra is placed at the distal end causing the pubic tips to appear separate in anterior view. It was only scored in *Archaeopteryx* and IGM 100/1126, but no specimen of the former preserves the pubic tip in anterior view and Pei (2015) describes the latter as having a standard deinonychosaurian "pubic slit." Thus the state is not added here yet, although it should be once more basal theropods are included.

304. Ilium - convexity of dorsal edge of postacetabular process - convex or straight (0); concave (1) (226 in Makovicky et al., 2005; 332 in Senter, 2007; 226 in Zanno et al., 2009; 700 in Brusatte et al., 2014). This codes for any concavity over the postacetabular process, even if the curvature of the total dorsal edge is convex (contra its equivalent in Brusatte et al. [2014- character 223]. Like Senter, it eliminates Makovicky et al.'s further stipulation of "brevis shelf extending caudal to vertical face of ilium" from state 1. Zanno et al.'s version is highly composite, with separate states for how relevant the brevis shelf is to the concavity. The state where concavity is unrelated to a posteriorly projecting brevis shelf has the additional specifier "postacetabular process squared" scored here as character 174 and there as 158, while their other concave state has the specifier "postacetabular process rounded or acuminate and severely reduced" which corresponds both to process shape and size. Brusatte et al.'s character 700 is stated as scoring for an ilium that is "convex anteriorly and straightens out posteriorly", which results in a concave dorsal postacetabular edge in the taxa they score as having it (tyrannosaurids and ornithomimosaurs). Notably their other state "smoothly convex or straight across entire length" actually describes two different conditions that are not obviously more similar to each other.

305. Ilium - posterolateral edge of postacetabular process in dorsal view - ventral edge does not project laterally compared to dorsal edge (0); ventral edge has lateral projection at posterior end of ilium (1) (227 in Makovicky et al., 2005; 227 in Zanno et al., 2009; 699 in Brusatte et al., 2014).

306. Pedal phalanx II-2 - length of proximoventral heel compared to height of distal condyles - absent (0); present but <44% (1); >43% (2) (ordered) (228 in Makovicky et al., 2005; 322 in Senter, 2007). This eliminates the composite variable of the extent of the articular ridge on to the heel, and quantifies Makovicky et al.'s "small" and "long" variables. Senter defines his states 1 and 2 in terms of heel narrowness, which seems more difficult to quantify and is also not scored for here.

307. Ischium - obturator notch distal to process - present (0); absent (1) (233 in Makovicky et al., 2005; 307 in Senter, 2007; 867 in Pei, 2015). To qualify as a notch, the concavity must extend proximally under part of the obturator process.

308. Ischium - angle between edges of obturator process - >29 degrees (0); <30 degrees (234 in Makovicky et al., 2005; 305 in Senter, 2007; 255 in Zanno et al., 2009; 327 in Senter, 2011; 809 in Brusatte et al., 2014). This excludes the assumption of a triangular process in Makovicky et al.'s original character, the additional composite state of obturator-pubis contact in Zanno et al.'s character (also scored in our character 184 and their character 170), or the additional composite state of angle to the shaft in Senter's (2011) character. The latter is an example of poor state formulation where state 0 is "not as in state 1" which has no reason to be considered homologous between taxa. We also quantify this character.

309. Metatarsals II and III - topology of dorsal surface of shaft - no tubercle developed on either metatarsal (0); tubercle for insertion of m. tibialis cranialis on center or medial edge of metatarsal II (1); tubercle for insertion of m. tibialis cranialis on lateral edge of metatarsal II or on metatarsal III (2) (ordered) (235 in Makovicky et al., 2005; states 1 and 2 separated after 235 in Turner et al., 2012).

310. Maxilla - concavity of anterodorsal margin at base of dorsal process - depth of concavity <4% of orbit+jugal height (0); >3% (1) (30 in Gohlich and Chiappe, 2006; 18 in Senter, 2011; 498 in Brusatte et al., 2014). If no concavity is present, the taxon is scored 0. This quantifies Gohlich and Chiappe's character and excludes their composite variable of anterior process proportion (scored as character 311 below). It also excludes the composite variable of Senter's character scoring anterior maxillary angle for taxa with our state 0.

311. Maxilla - length of anterior ramus, measured from point of maximum anterodorsal concavity - <150% of maxillary height at that point (0); >149% (1) (30 in Gohlich and Chiappe, 2006; 19 in Senter, 2011). If no concavity is present, the character is considered inapplicable. This quantifies the character differently than Gohlich and Chiappe or Senter (both have a cut-off point at 100%) to better match the former's taxonomic distribution. Gohlich and Chiappe's was a composite character as noted above, but Senter's was independant like ours.

312. Nasal - extent of antorbital fossa on lateral surface - absent (0); present (1) (31 in Gohlich and Chiappe, 2006). This is coded inapplicable in taxa with no contact between the nasal and antorbital fossa, which is covered by character 326. Gohlich and Chiappe include "minimal" extent under state 0, but to avoid quantification or subjectivity we score any extent as state 1.

313. External mandibular fenestra - length compared to mandibular length - >18% (0); 12-18% (1); <12% (2); absent (3) (ordered) (71 in Gohlich and Chiappe, 2006; separation of state 0 after 367 in Xu et al., 2011a; separation of state 3 after 253 in Li et al., 2010, 73 in Senter, 2010, 73 in Dal Sasso and Maganuco, 2011, 622 in Brusatte et al., 2014 and 864 in Pei, 2015). Gohlich and Chiappe only separated "large" from "reduced or absent" fenestrae, roughly equivalent to our states 0/1 and 2/3. Xu et al.'s "large" state was more restrictive, falling under our state 0. We have quantified each of these. Li et al., Senter and Dal Sasso and Maganuco all added a state for an absent fenestra, but the latter two incorrectly made it a state of their characters scoring for a surangular process invading the fenestra. Pei created a new character for absence of a fenestra, but this was already scored as a state of his character 622, weighting the condition.

314. Anterior teeth - texture of carinae - all serrated (0); at least some unserrated (1) (85 in Gohlich and Chiappe, 2006; 346 in Senter, 2007; 264 in Zanno et al., 2009; 256 in Li et al., 2010; 783 in Brusatte et al., 2014). This refers to premaxillary teeth, the first 1-4 dentary teeth, and often the first 1-4 maxillary teeth. Previous authors only score premaxillary teeth, but anterior dentary covary in all known cases, which also allows taxa known from mandibular remains to be scored.

315. Maxilla - posterior extent of teeth - posterior to anterior orbital margin (0); restricted anterior to anterior orbital margin but extend over half of maxillary length (1); restricted to less than anterior half of maxilla (2) (ordered) (88 in Gohlich and Chiappe, 2006; states 1 and 2 separated after 82 in Zanno et al., 2009 and 848 in Brusatte et al., 2014). This quantifies Zanno et al.'s "significantly rostral to the preorbital bar" for our state 2. Zanno et al. also scored maxillary teeth absent as an additional state for this character, while we score that as character 88. Either method is valid.

316. Metacarpal IV - presence - present (0); absent (1) (146 in Gohlich and Chiappe, 2006; 270 in Li et al., 2010). This is only coded when the manus is complete enough to expect the tiny metacarpal IV of coelurosaurs to be preserved. Gohlich and Chiappe's original character artificially limits state 0 to a fourth digit "largely limited to its metacarpal."

317. Ilium - ventral extent of pubic peduncle compared to ischial peduncle - equal or less (0); greater (1) (160 in Gohlich and Chiappe, 2006; 467 in Turner et al., 2012; 311 in Zanno et al., 2009; 462 in Brusatte et al., 2014). This is slightly different from Gohlich and Chiappe's states of "similar to" and "much greater", which also did not allow for less extensive pubic peduncles. It is equivalent to Turner et al.'s states except those don't allow for shorter pubic peduncles either.

318. Pubis - extent of puboischiadic plate proximally - obturator foramen pierces plate (0); obturator notch formed when foramen opens ventrally (1); obturator notch reduced so that posterior edge of ischial peduncle does not project distally (2) (ordered) (162 in Gohlich and Chiappe, 2006; 171 in Zanno et al., 2009; 707 in Brusatte et al., 2014; states 0 and 1 separated after 273 in Li et al., 2010).

319. Femur - proximal extent of anterior trochanter - completely distal to head (0); extends to lower half of head (1); extends at least to upper half of head (2) (ordered) (172 in Gohlich and Chiappe, 2006; states 1 and 2 separated after 310 in Senter, 2007, 325 in Zanno et al., 2009, 260 in Li et al., 2010 and 710 in Brusatte et al., 2014). All authors except Li et al. make composite characters by combining this with characters scoring for anterior and greater trochanter separation, and each compares anterior trochanter height to greater trochanter height. Our states 0 and 1 specify Gohlich and Chiappe's "short" and "tall" states. Note neither Senter's or Zanno et al.'s states allow scoring of taxa with anterior trochanters taller than their greater trochanters.

320. Astragalus - anteroposterior depth of ascending process - deep and blocky (0); shallow and laminar (1) (181 in Gohlich and Chiappe, 2006). Gohlich and Chiappe made Norell et al.'s character even more of a composite by adding this to their character scoring ascending process height, width and medial concavity. We separate it here.

321. Maxilla - dorsoventral position of maxillary fenestra - distance from ventral antorbital fossa rim <11% of orbit+jugal height (0); >10% (1) (237 in Turner et al., 2007a; 240 in Senter, 2007; 28 in Senter, 2011). If the maxillary fenestra is placed so anteriorly that the ventral fossa rim is angling anterodorsally, the distance is measured perpendicular to the rim at that position. This quantifies Turner et al.'s character. Senter (2007) included this variable in his composite character also scoring for fenestra size and shape, adding "small" to the state description and not specifying a shape. He later (2011) removed the "small" requirement and specified the other three states were not dorsally displaced. However, this is poor character construction as the other three states should be united by having a ventrally placed fenestra, but being unordered nothing ties them together.

322. Maxilla - depth of posterior process - minimal depth <16% orbit+jugal height (0); >15% (1) (238 in Turner et al., 2007a; 246 in Senter, 2007). Note this measures the minimum depth anterior to where the jugal and/or lacrimal overlie the element. This quantifies the dorsoventrally "narrow" and "wide"/"deep" states of Turner et al. and Senter (2007). Brusatte et al. (2014) used the same quantification for states 0 and 1 of their character 235. Characters 24 and 25 of Senter (2011) combine to equate to this character, each coding for a separate portion of the process (antorbital fossa and external surface respectively).

323. Maxilla - convexity of lateral surface surrounding maxillary fenestra - flat (0); concave, forming fossa (1) (239 in Turner et al., 2007a; 862 in Pei, 2015). This fossa may only excavate certain edges of the maxillary fenestra. We eliminate Turner et al.'s composite specifiers of the fossa being deep and necessarily posteriorly or posterodorsally open. Pei creates this character again for his matrix despite already scoring it as his character 236, weighting its presence.

324. Maxilla - size of external portion of dorsal process - robust (0); reduced (1); absent (2) (ordered) (240 and 244 in Turner et al., 2007a). Characters 240 and 244 of Turner et al. are here considered states of the same variable, with the reduced external portion of the dorsal process coded for as 244:1 being intermediate between the broadly exposed process of 240:0 and 244:0 and the absent lateral exposure of 240:1.

325. Maxilla - height of antorbital fossa portion of dorsal process - broadly contacts external portion of dorsal process (0); separated from external portion (1) (241 in Turner et al., 2007a). Turner (2008) separates the state in Aves from that in *Hesperornis* and *Jixiangornis*, but the condition in *Hesperornis* (Witmer, 1990) does not appear different from e.g. *Chauna* (DigiMorph Staff, 2001).

326. Nasal - contact with antorbital fossa - absent (0); present but does not contact antorbital fenestra (1); present and contacts antorbital fenestra (2) (ordered) (242 and 243 in Turner et al., 2007a; 490 in Brusatte et al., 2014). Turner et al.'s separate characters are here partially joined, as 242:1 cannot occur unless 243:1 does.

327. Premaxilla - contact with antorbital fossa - absent (0); present (1) (243 in Turner et al., 2007a; 238 in Senter, 2007). Contra to Turner et al.'s wording, the nasal may also participate in our state 1.

328. Jugal - contact with antorbital fenestra - absent (0); present (1) (246 in Turner et al., 2007a).

329. Splenial - concavity of posterior margin - convex to slightly concave (0); strongly concave (1) (250 in Turner et al., 2007a).

330. Premaxillary teeth - height of first tooth - less than twice height of premaxillary teeth 2-4 (0); more than twice height of premaxillary teeth 2-4 (1) (251 in Turner et al., 2007a; 82 in Senter, 2007; 138 in Senter 2011). Contra Senter (2007), the condition in *Caudipteryx* is not considered to deserve its own state for having all premaxillary teeth be tiny, since the first pair cannot be described this way.

331. Postorbital - angle of anterior process - <96 degrees from long axis of ventral process (0); >95 degrees (1) (4 in Senter, 2007). This angle goes through the distal point of each process and meets at the center of the element's body. Senter reinterpreted Norell et al.'s character as the angle of the anterior process instead of its curvature. We quantify Senter's states of "subhorizontal" and "diagonal" here.

332. Jugal - lateral exposure of posterior process - much of process exposed (0); most of process hidden by quadratojugal (1) (33 in Senter, 2007). Senter added this state to Norell et al.'s character scoring for posterior process cross section, making that character a composite. It is separated here.

333. Frontal - shape of suture with nasal - roughly straight or curved (0); W-shaped (1) (42 in Senter, 2007). Senter added this state to Norell et al.'s character scoring for the angle of the nasofrontal suture, making that character a composite. We separate it here, noting we measure the nasofrontal angle from its medial to its lateral edge regardless of how straight each suture line is between endpoints.

334. Cervical vertebrae - height of neural spines - taller than anteroposteriorly long (0); lower than anteroposteriorly long (1) (99 in Senter, 2007; 271 in Zanno et al., 2009; 660 in Brusatte et al., 2014). Senter made Norell et al.'s character scoring for anteroposterior neural spine length into a composite by creating a state for each combination of length and height. Being unordered, this only succeeds in hiding homology from the phylogenetics program, as now e.g. taxa with anteroposteriorly short neural spines will not be seen as homologous if their spines are different heights. We have separated neural spine height as its own character here. Zanno et al. proposed a character measuring neural spine height as more or less than neural arch height instead.

335. Caudal vertebrae - fusion of distal caudals - absent (0); present, fused into pygostyle (1) (unknown in juveniles) (121 in Senter, 2007; 121 in Turner et al., 2007b; 323 in Turner et al., 2012). Senter made Norell et al.'s character scoring for caudal vertebral number into a composite by adding a state for "<10 caudal vertebrae, followed by a pygostyle." This is especially true as pygostyles can also be present in taxa with more than ten caudals. Turner et al. (2007b) did the same by adding the state "very short, fused into pygostyle", which is also problematic as not all caudals are fused in pygostylians. We follow Turner et al. (2012) in separating caudal fusion as a different character.

336. Coracoid - anterior projection of anterodistal margin (medial projection of mediodistal margin in Aves) - posterior to or on level with anterior margin of scapulocoracoid boundary (0); anterior to anterior margin (medial to medial margin in Aves) of scapulocoracoid boundary (1) (136 in Senter, 2007; 136 in Turner et al., 2007b). Senter and Turner et al. both made Norell et al.'s character scoring for coracoid shape more of a composite by adding a state for a distally expanded coracoid. As many coracoids (e.g. ornithomimids) are already expanded posterodistally, we limit this character to anterodistal expansion and separate it from characters scoring for other aspects of coracoid shape. Note that contrary to Turner et al.'s (2012) formulation, this can also be true for relatively short coracoids (e.g. *Deinonychus*- Ostrom, 1969: Fig. 65B).

337. Skull - length compared to femur - >125% (0); 80-125% (1); 61-79% (2); <61% (3) (ordered) (232 in Senter, 2007; states 0 and 1 separate after 364 in Agnolin and Novas, 2011, 1 in Senter, 2011 and 470 in Gianechini et al., 2017; states 2 and 3 separate after 1 in Senter, 2011). Senter's (2007) original character was discontinuous, scoring >90% and <80%. We have chosen the latter value to distinguish our states 1 and 2. State 0 is based on Agnolin and Novas' character designed to group *Austroraptor* and *Buitreraptor* together. Senter (2011) had a similar character scoring for >129%. He also created a state for <60% which we use, although his new character is still discontinuous between 85% and 90%, and 115% and 130%.

338. External naris - dorsoventral placement - distance between ventral edge and alveolar edge <51% of orbit+jugal height (0); >50% (1) (234 in Senter, 2007). Senter's original character scored this compared to the dorsal orbital margin.

339. Maxilla - posterior extent of medial wall of antorbital fossa - length of antorbital fossa anterior to antorbital fenestra / antorbital fenestra length <46% (0); >45% (1) (239 in Senter, 2007; 17 in Senter, 2011). Senter used a different measure of comparison in both publications, first to the entire antorbital fossa, then to the maxilla itself. The former will get equivalent though smaller ratios, but the latter will be compromised by other factors such as anterior ramus length.

340. Maxilla - elongation of maxillary fenestra - height >50% of length (0); 25-50% (1); <25% (2) (ordered) (240 in Senter, 2007; 28 in Senter, 2011). This is part of Senter's composite character describing maxillary fenestra shape and position, using values measured from fenestrae he scored as being "round", "craniocaudally elongate oblong" and a "craniocaudally elongate slit" for our states 0-2. His 2011 update uses 33% as the cut-off point between the first two states but leaves the last state unquantified.

341. Nasals - median contact - sutured or non-contacting (0); fused (1) (241 in Senter, 2007; 254 in Turner et al., 2012; 346 in Zanno et al., 2009; 263 in Li et al., 2010). While it might be expected that this is unknown in juveniles, the smallest tyrannosaurids and even embryonic oviraptorids (Wang et al., 2016) have already fused their nasals.

342. Nasal - surface texture - smooth (0); rugose (1) (unknown in juveniles) (242 in Senter, 2007; 261 in Turner et al., 2012; 296 in Li et al., 2010).

343. Jugal - minimum suborbital height - >30% of orbital height (0); 20-30% (1); <20% (2) (ordered) (243 in Senter, 2007; states 1 and 2 separated after 53 in Senter, 2011). This quantifies Senter's (2007) qualifiers of "dorsoventrally stout" and "dorsoventrally narrow" but eliminates his composite variables of "short" versus "elongate." Senter's (2011) character is worded as scoring for cross section (platelike vs. rodlike) but more easily scores for an even more slender jugal so is here used as state 2.

344. Nasal - length compared to frontal - longer or equal (0); shorter (1) (244 in Senter, 2007).

345. Skull - ventral deflection of snout - angle between maxilla and jugal+quadratojugal ventral margins <30 degrees (0); >29 degrees (1) (247 in Senter, 2007; 55 in Senter, 2011). Senter's original wording specifies state 1 has a premaxilla forming an acute point, but that is already scored for here as character 228. Senter (2011) is better phrased as scoring for a ventral concavity, which we quantify here.

346. Maxilla - length of ventral margin - >125% of orbit+jugal height (0); <126% (1) (248 in Senter, 2007). Senter uses a character measuring preorbital length, but we used his scores to quantify maxillary length so as not to group taxa with long maxillae and long premaxillae as having homologous conditions.

347. Frontal - level of lateral edge of dorsal surface - equal or depressed compared to rest of frontal (0); raised rim (1) (249 in Senter, 2007).

348. Parietal - length compared to frontal - shorter (0); longer (1) (250 in Senter, 2007). Frontal length is the total length of the element, while parietal length is measured on the midline.

349. Laterotemporal fenestra - greatest anteroposterior length - >30% of orbit+jugal height (0); <31% (1) (251 in Senter, 2007). This quantifies Senter's "much shorter" qualifier for state 1 and compares it to orbit height instead of orbit length, since orbit length variation is covered by character 8.

350. Exoccipital and basioccipital - transverse width of foramen magnum - <101% of occipital condylar width (0); >100% (1) (252 in Senter, 2007; 257 in Zanno et al., 2009; 780 in Brusatte et al., 2014).

351. Dentary - depth of Meckelian groove - deep (0); shallow (1) (254 in Senter, 2007; 630 in Brusatte et al., 2014).

352. Dentary - posterior extent of ventral dentary - ends prior to or partly overlapping external mandibular fenestra (0); extends past external mandibular fenestra (1) (255 in Senter, 2007).

353. Surangular - lateral surface ventral to glenoid - flat (0); with horizontal ridge (1) (256 in Senter, 2007; 631 in Brusatte et al., 2014). This differs slightly from Senter's formulation which counted a "faint ridge" as state 0. This way ridge prominence doesn't have to be subjectively determined.

354. Premaxillary teeth - height of longest compared to longest maxillary tooth - >70% (0); <71% (1) (257 in Senter, 2007; 257 in Li et al., 2010; 647 and 747 in Brusatte et al., 2014). This quantifies Senter's and Li et al.'s "much smaller" and "significantly smaller" qualifiers. Note neither previous character had a way to score taxa with much larger premaxillary teeth. Also note Brusatte et al. have two characters scoring this condition, weighting it in their analysis.

355. Dentary teeth - presence at tip of dentary - present (0); absent for space of more than two FABLs from dentary tip (1) (261 in Senter, 2007; 220 in Zanno et al., 2009; 785 in Brusatte et al., 2014). This is scored inapplicable for taxa without dentary teeth. We quantify the distance necessary to count as state 1 of Senter's character. Zanno et al. added the state to their character scoring for dentary tooth extent. Yet given the state "only with teeth rostrally" in their character, any homology between e.g. anterior toothlessness and complete toothlessness is hidden from the phylogenetics program.

356. Cervical vertebrae - elongation - centrum length less than centrum height (0); equal to twice as long (1); length more than twice height (2) (ordered) (262 in Senter, 2007; 268 in Zanno et al., 2009; 786 in Brusatte et al., 2014; states 0 and 1 separated after 164 in Senter, 2011 and 652 in Brusatte et al., 2014). In future analyses, this should be specified to a region of the cervical column, as elongation varies between vertebrae. Senter (2007) compared mid cervical length to dorsal length, while Zanno et al. compared anterior cervical length to minimal cervical centrum width. We followed Senter (2011) in comparing length to centrum height.

357. Dorsal vertebrae - height of neural spines of posterior dorsals (6-13) - >149% taller than long (0); >75% of posterior central height (1); <76% (2) (ordered) (265 in Senter, 2007; 756 in Brusatte et al., 2014; states 1 and 2 separated after 277 in Zanno et al., 2009). Brusatte et al.'s character merely scores for "much higher" spines.

358. Dorsal vertebrae - posterior extent of postzygapophyses on mid and posterior dorsals (4-13) - end above centrum (0); extend posterior to centrum (1) (266 in Senter, 2007).

359. Chevrons - length of proximal chevrons compared to caudal centrum length - >149% (0); <150% (1) (267 in Senter, 2007).

360. Furcula - interclavicular angle, measured from center of median to tips of arms - >69 degrees (0); <70 degrees (1) (268 in Senter, 2007; 335 in Turner et al., 2012). Note Senter's version of this character is discontinuous, with no state for angles between 60 and 80 degrees. Turner et al. use a different cut-off point of 90 degrees for their character.

361. Scapula - angle of posterior edge of acromion - <80 degrees compared to long axis of proximal shaft (0); 80-20 degrees (1); <20 degrees (2) (ordered) (270 in Senter, 2007; 258 in Li et al., 2010; 259 in Foth et al., 2014). Future analyses might incorporate character 245 from Xu et al. (2009), to further separate those taxa such as *Rahonavis* and *Sapeornis* whose posterior acromion edge angles anteroventrally. Senter's acromion character is a composite scoring both angle and curvature and having the problematic state "does not match any of the following descriptions" that has no basis for being homologous between taxa. We quantify posterior angle here, corresponding to Senter's states 1-3. Li et al. used a bistate character that also mixed angle and shape without quantification. Foth et al. recently used a bistate character but only for acromion angle that corresponds to our quantified states 0 and 1/2.

362. Scapula - angle of anterior edge of acromion - <106 degrees compared to dorsal edge of shaft just posterior to it (0); >105 degrees (1) (270 in Senter, 2007; 258 in Li et al., 2010). This corresponds to part of state 3 of Senter's character and part of state 1 of Li et al.'s, though we quantify it for the first time.

363. Scapulocoracoid - shape of dorsal edge at suture - notched (0); smooth (1) (271 in Senter, 2007). This only applies in taxa where the acromion is not widely separated from the coracoid (state 0 for character 252).

364. Scapula - expansion of posterior end - >184% of minimum shaft width (0); <185% (1); tapered (2) (ordered) (272 in Senter, 2007; state 2 added from 352 in Turner et al., 2012, 246 in Xu et al., 2009 and 283 in Zanno et al., 2009). States 0 and 1 quantify "wide" in Senter's character.

365. Coracoid - position of coracoid tuber - distal to scapular articulation (0); proximal to scapular articulation, forming acrocoracoid process (1) (273 in Senter, 2007; 347 in Turner et al., 2012).

366. Humerus - minimum width in anterior view compared to length - >12% (0); <13% (1) (275 in Senter, 2007). Senter's version measures shaft length "between deltopectoral

crest and distal condyles", but this would vary based on deltopectoral length (character 147 here). We have used his taxon scores to instead score against total humeral length.

367. Ulna - shape of posterior margin - straight, sigmoid or concave (0); convex (1) (276 in Senter, 2007; 294 in Zanno et al., 2009; 688 in Brusatte et al., 2014). Any concavity caused by a projected dorsal condyle as in most birds is not counted against total convexity.

368. Distal carpals I and II - shape of combined proximal surface - flat or irregular (0); strongly and smoothly convex (1) (279 in Senter, 2007; 148 in Zanno et al., 2009; 276 in Li et al., 2010; 689 in Brusatte et al., 2014). This defines the semilunate carpal. Zanno et al. have a composite character scoring carpal flatness as well as carpal fusion. Li et al.'s character is also a composite, scoring the development of a carpal trochlea along with flatness. Yet there are taxa with a flat carpal and trochlea (e.g. *Afrovenator*). Senter (2011; his character 243) adds the intermediate state "shallowly convex" for *Guanlong*+tyrannosaurids, alvarezsauroids, therizinosaurs and basal oviraptorosaurs, but there is no obvious difference as illustrated by Xu et al. (2014; Fig. 2).

369. Metacarpal I - height of medial condyle ventrally compared to lateral condyle - higher (0); lower (1) (281 in Senter, 2007).

370. Metacarpal II - medial expansion of proximal end - much greater than distal width, >119% (0); subequal or less than distal width, <120% (1) (282 in Senter, 2007). This quantifies Senter's character.

371. Metacarpal III - length compared to metacarpal II - >79% (0); <80% (1) (283 in Senter, 2007; 297 in Zanno et al., 2009). We use Senter's quantification. Zanno et al.'s merely says "longer" or "subequal."

372. Metacarpal III - distal extent of contact with metacarpal II - extensive intermetacarpal space (0); metacarpals in contact for almost all of their length (1) (286 in Senter, 2007).

373. Metacarpal III - position of proximal end - mostly lateral to metacarpal II (0); mostly ventral to metacarpal II (1) (287 in Senter, 2007).

374. Manual phalanx II-2 - length compared to II-1 - <101% (0); 101-119% (0); 120-200% (1); >200% (2) (ordered) (288 and 293 in Senter, 2007; states 0 and 1 separated after 400 in Turner et al., 2012). Senter uses two correlated characters to score for this variable.

375. Manual phalanx II-1 - expansion of lateral edge - fully concave (0); with low convexity developed on at least portion of shaft, maximum shaft width <161% of minimum width of metacarpal II (1); with deep convexity, >160% of minimum width of metacarpal II (2) (ordered) (unknown in young juveniles) (290 in Senter, 2007; 264 in Senter, 2011; states 1 and 2 separated after 399 in Turner et al., 2012). We newly quantify state 2 from Turner et al.'s character, which emphasizes flattening instead of lateral expansion.

376. Manual digit II - length of II-1 and II-2 compared to metacarpal II - >100% (0); <101% (1) (291 in Senter, 2007; 300 in Zanno et al., 2009; 799 in Brusatte et al., 2014). Note unlike Senter's version, we exclude carpal length from the metacarpal measurement.

377. Manual phalanx I-1 - ventral curvature - straight (0); bowed (1) (295 in Senter, 2007).

378. Manual ungual I - dorsal projection of dorsal curve - with articular surface oriented vertically, dorsal edge of at least mid portion of ungual does not project dorsally over the level of the proximodorsal ungual corner (0); dorsal edge of at least mid portion of ungual does project dorsally over the level of the proximodorsal ungual corner (1) (296 in Senter, 2007; 857 in Cau et al., 2015). Note this excludes the condition where only the proximal portion of the ungual projects dorsally. Cau et al. lose information by combining this and the following into a single character scoring all manual unguals.

379. Manual ungual II - dorsal projection of dorsal curve - with articular surface oriented vertically, dorsal edge of at least mid portion of ungual does not project dorsally over the level of the proximodorsal ungual corner (0); dorsal edge of at least mid portion of ungual does project dorsally over the level of the proximodorsal ungual corner (1) (297 in Senter, 2007; 857 in Cau et al., 2015). As with the previous character, this excludes the condition where only the proximal portion of the ungual projects dorsally. Foth and Rauhut (2017; character 284) also include manual ungual III in this character, but it has not been shown these digits covary.

380. Manual phalanx III-3 - length compared to combined lengths of III-1 and III-2 - <93% (0); 93-109% (1); >109% (2) (ordered) (302 in Senter, 2007; 301 in Zanno et al., 2009). This quantifies Senter's "markedly shorter", "subequal" and "markedly longer" states. Zanno et al. use a binary character with a cut-off at 100%.

381. Ilium - height of preacetabular process, dorsal extent over line drawn between bases of peduncles, compared to height at base of postacetabular process - <90% (0); 90-150% (1); >150% (2) (ordered) (303 in Senter, 2007; 305 and 307 in Zanno et al., 2009; states 0 and 1 separated after 370 in Xu et al., 2011a). Each process is measured at their base, where the ventral edge becomes more horizontal than vertical. State 2 quantifies Senter's states of "absent or small" versus "extreme arching over the postacetabular process. Zanno et al. and Brusatte et al. use two correlated characters for this concept, one scoring for the height of the preacetabular process and the other for the angle of the preacetabular process' dorsal edge. Our state 0 quantifies Xu et al's "shallow" preacetabular process.

382. Ischium - minimum anteroposterior depth of shaft distal to obturator process compared to pubic shaft - <80% (0); 80-130% (1); >130% (2) (304 in Senter, 2007). Note while Senter coded most paravians as having deep ischia, this is generally due to their extensive and distally located obturator processes. Thus characters that use midshaft depth (e.g. 163 in Brusatte et al., 2014) are comparing non-homologous portions of the ischia. Taxa with reduced post-obturator shafts are here coded as inapplicable (e.g. *Falcarius*, *Velociraptor*, *Archaeopteryx*). Of taxa which lack obturator projections, pygostylians and their close relatives are coded as inapplicable since basal paravians have almost no shaft distal to their obturator process and even taxa with narrow ischia like *Apsaravis* have a ventral ridge along most of their ischium. However, alvarezsaurid ischia are measured at their narrowest point. This is because e.g. *Linhenykus'* ischium has a flat ventral cross section, suggesting homology with a shaft, which is also elongate in proposed relatives such as *Haplocheirus* and ornithomimosaurs. Scansoriopterygids are coded unknown since no specimen reveals the ischial cross section and relationships are controversial. This quantifies Senter's states of "slenderer", "subequal" and "thicker." Brusatte et al. (2014) add a state to their character 163 distinguishing ischial shafts 30-50% of pubic diameter from those 60-100% of pubic diameter. The sole maniraptoromorph scored as being 30-50% is Xixianykus, which actually has a deeper ischium than pubis (Xu et al., 2010: Fig. 6A-B). Thus the extra state is not added here although it may be justified with the inclusion of tyrannosauroids.

383. Tibia - length compared to femur - shorter or equal (0); longer (1) (309 in Senter, 2007; 319 in Zanno et al., 2009; 717 in Brusatte et al., 2014). Note Brusatte et al.'s states are discontinuous, with no state covering tibiofemoral ratios of 1.00-1.04.

384. Tibia - anterior constriction at base of lateral condyle in proximal view - lateral condyle does not laterally overlap incisura tibialis (0); overlap present (1) (311 in Senter, 2007; 718 in Brusatte et al., 2014).

385. Metatarsus - length compared to femur - >49% (0); 37-49% (1); <37% (2) (ordered) (312 in Senter, 2007; states 1 and 2 separated after 334 in Zanno et al., 2009 and 823 in Brusatte et al., 2014). Note Zanno et al. use a slightly different ratio of >45% for their equivalent to our state 0, and also had a discontinuous character with no states for 41-45% or 36-37%. Brusatte et al. narrowed these discontinuous states to 45% and 36-37%.

386. Metatarsal II - depth at midshaft - less or equal to transverse width at midshaft (0); greater than transverse thickness (1) (313 in Senter, 2007). As noted above, Senter's character is a composite that scores for all metatarsals. Metatarsal IV is scored by our character 220.

387. Metatarsus - distal extent of metatarsal II-IV contacts - shafts separated (0); shafts appressed (1) (314 in Senter, 2007; 335 in Zanno et al., 2009; 287 in Li et al., 2010; 725 and 824 in Brusatte et al., 2014). Senter (2011) modifies this character (his 356) to separate taxa with straight non-appressed metatarsals from those with distally diverging non-appressed metatarsals, though no taxon was actually scores as having the former condition. As this a priori assumes the nonhomology of each kind of non-appressed metatarsus condition, and both conditions can occur in the same foot (e.g. metatarsal II is straight and non-appressed in *Dilophosaurus*' holotype, while metatarsal IV diverges to be non-appressed, though it was coded as having appressed metatarsals by Senter), metatarsal curvature should be coded as independent characters. Note Brusatte et al. used two characters for this, so weighted its importance.

388. Metatarsus - minimum transverse width distally compared to proximal width - >59% (0); <60% (1) (316 in Senter, 2007). This measures the minimum width proximal to the distal condylar expansion compared to the maximum width at the proximal end of the (tarso-)metatarsus, and only includes metatarsals II-IV. It quantifies Senter's "marked decrease in transverse width."

389. Metatarsal I - torsion - absent (0); present, so that hallux faces medially or posteriorly (1) (317 in Senter, 2007; 432 in Turner et al., 2012). Note this cannot be determined by the orientation of the hallucial digit in a flattened specimen, but depends on twisting present in the metatarsal. Turner et al.'s character is a composite, with this as their state 2. Their state 1 is character 672 below.

390. Pedal ungual I - length on straight line compared to ungual III - <90% (0); >89% (1) (318 in Senter, 2007). This quantifies Senter's qualifier "not reduced in size relative to other pedal unguals."

391. Manual ungual III - length on straight line compared to ungual II - <96% (0); >95% (1) (326 in Senter, 2007). This quantifies Senter's qualifier of unguals II and III "approximately the same size."

392. Metatarsus - transverse slenderness at midshaft - >50% of length (0); 30-50% (1); 13-29% (2); <13% (3) (ordered) (335 in Senter, 2007; state 0 separated after 333 in Zanno et al., 2009 and 822 in Brusatte et al., 2014). This only includes metatarsals II-IV.

393. Ilium - anteroposterior length of pubic peduncle, measured halfway down - over twice as long as ischial peduncle measured halfway down (0); less than twice, but still longer than ischial peduncle (1); equal or shorter (2) (ordered) (698 in Brusatte et al., 2014; 337 in Senter, 2007; 361 in Zhang et al., 2008; states 0 and 1 separated after 292 in Li et al., 2010). Peduncle length is perpendicular to its long axis.

394. Pedal phalanx III-1 - slenderness - length >139% transverse width (0); <140% (1) (338 in Senter, 2007; 729 in Brusatte et al., 2014). In the absence of III-1, other phalanges may be used to determine if the taxon has shortened phalanges as in derived therizinosaurs. Note Senter's quantification is slightly different with the cut-off point at 200%. Brusatte et al.'s is as well, using a ratio of 300% of midshaft width so that *Tarbosaurus* and *Tyrannosaurus* (which has a ratio of 166% under our parameters) fall under their state 1 as well.

395. Humerus - transverse width of distal end - <200% minimum shaft width (0); 200-269% (1); >269% (2) (ordered) (339 in Senter, 2007; 293 in Zanno et al., 2009; 796 in Brusatte et al., 2014). We use the quantification of Zanno et al., which Brusatte et al. lost data from by merging their states 1 and 2. Senter quantifies this compared to humeral length, which could correlate with humeral robusticity (our character 366).

396. Humerus - transverse extent of ectepicondyle - <20% of distal humeral width (0); >19% (1) (340 in Senter, 2007). We quantify Senter's state of an ectepicondyle "expanded laterally."

397. Metatarsal I - transverse width of distal condyle - <81% of transverse width of distal condyle of metatarsal III (0); >80% (1) (341 in Senter, 2007). We quantify Senter's state of metatarsal I being "comparable in size to distal ends of other metatarsals." Comparing only metatarsal III is important as other trochlea can be much wider or narrower (our characters 674 and 675).

398. Metacarpal II - length compared to humerus - >32% (0); <33% (1) (344 in Senter, 2007).

399. Jugal - dorsoventral expansion of anterior process - expansion dorsal to ventral orbit margin <6% of orbit+jugal height (0); >5% (1) (347 in Senter, 2007; 262 in Turner et al., 2012; 529 and 543 in Brusatte et al., 2014). While Senter's character specifies no expansion, a minor amount is present in several taxa so that the quantification was changed to match his character scores. Turner et al.'s state is part of a composite character also scoring for anterior process bifurcation (here scored as character 539), so is separated here.

400. Chevrons - anteroventral process on distal chevrons - absent (0); present (1) (349 in Senter, 2007).

401. Metacarpal III - depth of distal articular groove - absent (0); present (1) (350 in Senter, 2007; 296 in Zanno et al., 2009; 856 in Cau et al., 2015).

402. Scapula - dorsoventral depth of acromion - >20% of total scapular depth (0); <21% (1) (351 in Senter, 2007; 242 and 244 in Xu et al., 2009; 282 in Zanno et al., 2009). Acromion depth is measured from a line continuing the dorsal scapular shaft edge proximally. This quantifies Senter's qualifier of "shallow", Xu et al.'s "minor" and Zanno et al.'s "poorly developed, does not rise significantly dorsal to longitudinal axis of scapular blade." While Xu et al.'s (2009) character 242 separated acromion depth from acromion cross section, the latter depends on the former.

403. Metatarsus - medial extent of metatarsal IV posterior to III - extensive, often leading to L-shaped metatarsal III (0); slight to absent (1) (352 in Senter, 2007; 336 in Zanno et al., 2009).

404. Nasals - transverse convexity of dorsal surface - concave to slightly convex (0); highly convex, forming median crest (1) (353 in Senter, 2007; 264 in Li et al., 2010; 506 in Brusatte et al., 2014). Senter also specified premaxillary and frontal involvement, but those are not necessary here. Li et al. used a composite character also scoring for parasagittal crests, here separated as character 500.

405. Ectopterygoid - anteroposterior position - posterior to palatine (0); lateral to palatine (1) (354 in Senter, 2007; 258 in Zanno et al., 2009; 781 in Brusatte et al., 2014). We use Zanno et al.'s comparison to palatine position instead of Senter's comparison to pterygoid position.

406. Pterygoid - orientation of palatal process - within 20 degrees of ventral jugal edge, palatal process completely dorsal to ventral margin of jugal (0); projects strongly downward (>20 degrees), palatal process mostly ventral to jugal (1) (355 in Senter, 2007). This is a quantified version of Senter's character scoring for a palate arching below the external skull surface, as the lateral pterygoid and ectopterygoid commonly do to a small degree.

407. Articular - contact with surangular - sutured (0); fused (1) (unknown in juveniles) (356 in Senter, 2007). Note Senter (2007) lists this character as describing angular-surangular fusion, but references Osmolska et al. (2004) which instead had a character involving the articular.

408. Cervical ribs - contact with vertebrae - mobile or sutured (0); fused (1) (unknown in juveniles) (357 in Senter, 2007).

409. Caudal vertebrae - pneumaticity of caudal centra 1-5 - absent (0); pleurocoels present (1) (359 in Senter, 2007; 279 in Zanno et al., 2009; 791 in Brusatte et al., 2014).

410. External mandibular fenestra - anteroposterior placement - length of mandible posterior to fenestra <140% orbit+jugal height (0); >139% (1) (360 in Senter, 2007). This quantifies Senter's character describing a "rostrally displaced" fenestra.

411. Manual ungual II - presence - present (0); absent (1) (151 in Turner et al., 2007b; 474 in Brusatte et al., 2014). Turner et al. added the new state "manual unguals absent" to Norell et al.'s already composite character scoring for manual ungual curvature, flexor tubercle size and tubercle position. As "absent" is not a correct variable for any of these, we have separated it as a new character and specified ungual II. Manual ungual III is covered by character 159, and manual ungual I is usually present even in basal Aves (Fisher, 1940).

412. Ilium - convexity of anterior edge - convex or flat (0); with concavity (1) (156 in Turner et al., 2007b; 262 in Li et al., 2010; 156 in Senter, 2010; 235 in Dal Sasso and Maganuco, 2011). Turner et al. and Senter added this state to Norell et al.'s character describing anterior preacetabular edge angle, making it a composite. We follow Li et al. and Dal Sasso and Maganuco in separating it.

413. Basioccipital - basal tubera size - present (0); absent (1) (225 in Turner et al., 2007b; 254 in Zanno et al., 2009). Turner et al. added this to Hwang et al.'s already composite character describing basal tuber morphology, but as "absent" is not a proper variable for these it is separated here. Zanno et al. created a new character scoring tubera as "well developed", "reduced" or "absent", but no quantifiable difference is obvious between maniraptoromorphs they scored as state 0 (*Dromaeosaurus* and *Velociraptor*) and other taxa such as troodontids or ornithomimids.

414. Postorbital - length of anterior process - shorter than ventral process (0); equal or longer than ventral process but without thin extension along orbit (1); longer than ventral process, with thin extension along orbit (2) (ordered) (236 in Novas et al., 2008; 551 in Brusatte et al., 2014; state 2 separated after 252 in Li et al., 2010). Both processes measured from opposite edge of element. Note that while Choiniere et al. (2012) found a frontal groove along the orbital margin did not always correspond to state 2 (e.g. *Gallimimus*, *Shuvuuia*), the state is retained here as ordering leaves it an autapomorphic extension of state 1 and both the *Compsognathus longipes* and *C. corallestris* possess it in case these are analyzed as separate OTUs in the future if their synonymy is rejected (e.g. Rauhut and Foth, 2014). Brusatte et al. (2014) used a ratio of 60% to divide the two states of his character 551, but of those taxa scored as >60%, *Dilong* and *Compsognathus* are already scored 1 here, *Nqwebasaurus* doesn't preserve the element, and the anterior process length in *Guanlong* is unclear due to preservation. This leaves only *Juravenator* correctly scored with an elongate process shorter than the ventral process, so a new state is not added here.

415. Postorbital - angle between ventral and posterior processes (measured with vertex equidistant between orbit, supratemporal and laterotemporal fenestrae, through tips of processes) - <120 degrees (0); >119 degrees (1) (237 in Novas et al., 2008). This quantifies Novas et al.'s states "sharply flexed" and "gently concave."

416. Scapula - medial curvature - present, anterior end placed medially to blade (0); absent (1) (239 in Xu et al., 2009).

417. Scapula - sectional shape of posterior half - thin blade (0); ventrally thick, drop-shaped (1) (247 in Xu et al., 2009).

418. Coracoid - convexity of medial (posterior in Aves) surface - flat (0); with fossa (1) (248 in Xu et al., 2009; 342 in Turner et al., 2012). This eliminates the "distinctly oval-shaped" qualifier of fossa shape from Xu et al.'s character.

419. Ulna - height of coronoid process - prominent (0); low or absent (1) (254 in Xu et al., 2009).

420. Ulna - height of proximomedial process - low or absent (0); prominent (1) (256 in Xu et al., 2009).

421. Ulna - anterior surface of proximal third - smooth (0); with longitudinal ridge (1) (257 in Xu et al., 2009). This eliminates the "thick" qualifier for ridge morphology from Xu et al.'s character.

422. Ulna - proximal extent of distal articular surface up posterolateral side - slight (0); over half of ulnar depth (1) (ordered) (258 in Xu et al., 2009; 383 in Turner et al., 2012). This qualifies Xu et al.'s "significant" extent, but in a different way than Turner et al. who compare it to transverse articular surface width, generally unmeasurable in slab specimens.

423. Ulna - position of thickest portion of distal end - near medial margin (0); near middle or lateral margin (1) (260 in Xu et al., 2009). Unlike Xu et al.'s character formulation, this accounts for taxa whose thickest portion is laterally positioned.

424. Ulna - anterolateral surface distally - convex to shallowly depressed (0); well defined radial sulcus extending to distal articulation (1) (261 in Xu et al., 2009).

425. Ulna - proportions of distal end - transversely compressed to equilateral (0); transverse width greater, but less than 201% (1); transverse width greater than 200% (2) (ordered) (262 in Xu et al., 2009).

426. Radius - dorsolateral projection of distal end - absent (0); present, forming flange (1) (263 in Xu et al., 2009).

427. Radiale - size - <90% of distal ulnar depth (0); >89% (1) (264 in Xu et al., 2009). This quantifies Xu et al.'s qualifiers of "small" and "enlarged."

428. Ulnare - convexity of distal edge - convex to straight (0); slightly concave (1-15% of ulnare depth measured perpendicular to line across tips of rami), forming metacarpal incisure to make ulnare heart-shaped (1); deeply concave (>15%), making ulnare V-shaped (2) (ordered) (265 in Xu et al., 2009; state 2 separated after 388 in Turner et al., 2012). This quantifies Turner et al.'s qualifiers of heart-shaped and V-shaped.

429. Ilium - ventral extent of postacetabular process - completely dorsal to ischial peduncle tip (0); extends ventral to ischial peduncle (1) (270 in Xu et al., 2009; 294 in Senter, 2011). Senter distinguishes between three states of ventral extent- far dorsal, about at level and far ventral. However, he also codes this relative to both pubic and ischial peduncles. In Senter's matrix, only alvarezsaurids have state 1 while only paravians have state 2, which is due to the later having longer pubic peduncles. As the disparity in peduncle ventral extent is already coded in this matrix (character 317), the extra state is not used here.

430. Sternum - posterior expansion of posteromedian process - absent (0); present but posteriorly convex (1); present and posteriorly concave (2) (ordered) (new to TWiG; 125 in Cau and Arduini, 2008). This is considered to be inapplicable in taxa where the posteromedian process joins the posteromedial processes.

431. Pubis - distal extent of posterior median groove - proximally restricted, posterodorsal edge of pubic boot rounded (0); distally extending between most of pubic boots, posterodorsal edge of pubic boot sharply ridged forming hypopubic cup (1) (276 in Xu et al., 2009).

432. Tibia - midshaft transverse diameter compared to femur - >80% (0); <81% (1) (278 in Xu et al., 2009). This quantifies Xu et al.'s qualifiers of "subequal to" and "more robust" and allows scoring of taxa with more gacile femora. Note that while Xu et al. scored femorotibial ratios, we have reversed it to score tibiofemoral ratios.

433. Femur - posterior surface of femoral head - transversely flat to convex (0); with vertical groove near medial margin (1) (279 in Xu et al., 2009; 324 in Zanno et al., 2009). Note our character has reversed scoring compared to Xu et al.'s or Zanno et al.'s where flat was state 1.

434. Manual phalanx I-1 - convexity of distoventral surface proximal to condyles - convex to flat (0); concave to form flexor fossa (1) (new to TWiG, originally 148 in Sereno, 1999 Tetanurae list). This is modified to only code for phalanx I-1 instead of all proximal phalanges, based on the discussion in Suzuki et al. (2002).

435. Tibia - angle of anterior edge of cnemial crest - >10 degrees from anterior shaft edge (0); <11 degrees (1) (282 in Xu et al., 2009). This quantifies Xu et al.'s qualifiers of "prominent" versus "small" crests.

436. Tibia - orientation of cnemial crest in proximal view - mostly anterior (0); mostly lateral (1) (283 in Xu et al., 2009).

437. Tibia - proximal extent of fibular crest - separate from lateral condyle (0); continuous with lateral condyle (1) (284 in Xu et al., 2009).

438. Braincase - prominence of crista prootica - slight convexity to small ridge (0); well developed ridge (1); prominent overhanging ala (2) (ordered) (448 in Turner et al., 2012; states 0 and 1 separated from 468 in Turner et al., 2012).

439. Astragalus - contact between ascending process and fibula - absent (0); present (1) (196 in Zanno et al., 2009; 820 in Brusatte et al., 2014). Zanno et al. added this to Norell et al.'s composite character on ascending process morphology, making it even more composite as taxa with both short and tall processes can have fibular contact. It is here separated as in Brusatte et al.'s analysis.

440. Basisphenoid - expansion - unexpanded (0); expanded ventrally, posteriorly and laterally due to extensive pneumatization (1) (253 in Zanno et al., 2009; 779 in Brusatte et al., 2014). This is meant to code for the inflated basicranium of therizinosaurids.

441. Supraoccipital - posterior median surface - pronounced sagittal crest (0); concave to slightly convex (1) (256 in Zanno et al., 2009; 256 in Turner et al., 2012). Note Turner et al.'s version has the opposite polarity.

442. Vomer - posterior extent - ends anterior to subtemporal fenestra (0); extends posterior to anterior edge of fenestra (1) (259 in Zanno et al., 2009). Note Zanno et al. defined state 1 as posterior to the suborbital fenestra, which is slightly different and potentially results in unscorable conditions where the vomer terminates between the fenestrae.

443. Dentary - angle of symphysis - >45 degrees from dorsal dentary margin (0); <46 degrees (1) (261 in Zanno et al., 2009; 624 in Brusatte et al., 2014). This quantifies Zanno et al.'s states of "vertical to subvertical" and "projects strongly cranially, obliquely oriented with respect to ventral margin of dentary."

444. Dentary teeth - labiolingual compression of teeth 1-2 - labiolingual width <90% of FABL (0); >89% (1) (263 in Zanno et al., 2009; 784 in Brusatte et al., 2014). This quantifies Zanno et al.'s states of "not conical" and "conical." Note Brusatte et al. only score this as state 1 if at least dentary teeth 1-4 are conical.

445. Teeth - labiolingual compression of lateral tooth crowns - labiolingual width <80% of FABL (0); >79% (1) (267 in Zanno et al., 2009; 141 in Senter, 2011; 651 in Brusatte et al., 2014; 754 in Brusatte et al., 2014). This quantifies Zanno et al.'s states of "labiolingually compressed" and "conical" and separates this variable from others (e.g. recurvature) in their character. It does the same for Senter's composite character that uses "ziphodont" and "conical." Note Brusatte et al. have two characters scoring for this, one exclusively, and one in a composite also involving recurvature and basal constriction.

446. Cervical vertebrae - transverse convexity of ventral centum surface - convex or flat (0); concave (1) (269 and 270 in Zanno et al., 2009; 303 in Makovicky et al., 2012; 787 and 788 in Brusatte et al., 2014). Zanno et al. distinguished two characters, for concavities with and without prominent ventrolateral crests, but this was not considered consistent or well defined enough to include.

447. Dorsal vertebrae - height of neural arch in mid dorsals (4-6) - distance from neurocentral suture to prezygapophysis less than height of anterior articular surface (0); greater (1) (272 in Zanno et al., 2009; 789 in Brusatte et al., 2014). Note Zanno et al. specify "cranial" dorsal vertebrae for this character and the three following (447-450; their 272-275), but Zanno's (2010a) *Falcarius* osteology notes these follow the "cervicodorsal" vertebrae so equate to the middle of the series.

448. Dorsal vertebrae - diameter of parapophysis on mid dorsals (4-6) - <66% height of anterior articular surface (0); >65% (1) (273 in Zanno et al., 2009; 790 in Brusatte et al., 2014). Note Zanno et al.'s character was originally discontinuous, lacking any state for taxa measuring 50-66%.

449. Dorsal vertebrae - division of infrazygapophyseal fossae in mid dorsals (4-6) - undivided (0); accessory centrodiapophyseal laminae present (1) (274 in Zanno et al., 2009).

450. Dorsal vertebrae - inclination of diapophysis in mid dorsals (4-6) - inclined or horizontal (0); declined (1) (275 in Zanno et al., 2009).

451. Dorsal vertebrae - anterior bifurcation of neural spine in dorsal view - absent (0); present (1) (276 in Zanno et al., 2009). We separate anterior and posterior bifurcation since unlike Zanno et al.'s character formulation, anterior bifurcation can happen without posterior bifurcation (scored for in 452 below). We also eliminate the necessity of bifurcation to transversely expand the neural spine, leading to Zanno et al.'s descriptors of "subtriangular" and "medially pinched" spine shapes in dorsal view.

452. Dorsal vertebrae - posterior bifurcation of neural spine in dorsal view - absent (0); present (1) (276 in Zanno et al., 2009).

453. Sacral vertebrae - fusion of neural spines - all separate (0); at least some fused into a continuous lamina (1) (unknown in juveniles) (278 in Zanno et al., 2009; 670 in Brusatte et al., 2014).

454. Furcula - curvature of arms - straight or curved laterally to form V-shape (0); curved medially to form U-shape (1) (281 in Zanno et al., 2009; 470 in Turner et al., 2012). Note Zanno et al.'s formulation has no way to score taxa with laterally curved arms.

455. Scapula - dorsal edge of scapular blade - straight or evenly curved (0); with flange (1) (284 in Zanno et al., 2009; 792 in Brusatte et al., 2014).

456. Humerus - size of bicipital crest - low, projected <45% of minimum humeral width from humeral head (0); moderate, projected 45-100% of minimum humeral width (1); prominent, projected >100% of minimum humeral width (2) (ordered) (285 in Zanno et al., 2009; 763 in Brusatte et al., 2014). This is often called the internal or medial tuberosity in non-birds, but is homologous with the bicipital crest of birds, not the ventral tubercle of birds which is smaller and restricted to the posterior side of the bicipital crest. It quantifies Zanno et al.'s qualifiers "poorly developed", "well developed" and "hypertrophied" from their composite character also describing bicipital crest orientation, shape and separation from the humeral head.

457. Humerus - angle of bicipital crest in proximal view - <45 degrees from long axis of humeral head (0); angled laterally/posteriorly 45 degrees or more from long axis of humeral head (1) (285 in Zanno et al., 2009). This quantifies Zanno et al.'s states for medial or caudal orientation, and separates them from other variables in their character.

458. Humerus - separation of bicipital crest from humeral head - confluent (0); separated laterally (posteriorly in Aves) by capital groove (1) (285 in Zanno et al., 2009; 359 in Turner et al., 2012; 763 in Brusatte et al., 2014). This separates the state from Zanno et al.'s composite character which paired a capital groove with hypertrophied bicipital crest size. Turner et al. used Clarke's character scoring for the groove.

459. Humerus - shape of bicipital crest - with convex outer edge (0); with straight to concave outer edge (1) (285 in Zanno et al., 2009; 259 in Li et al., 2010; 763 in Brusatte et al., 2014). This separates the qualifier "rectangular" from Zanno et al.'s composite character, as in Li et al.'s character scoring for a pointed versus straight edge.

460. Humerus - posteromedial surface of midshaft - flat (0); with crest (1) (287 in Zanno et al., 2009; 793 in Brusatte et al., 2014).

461. Humerus - anterior surface proximal to ectepicondyle - flat (0); with tuberosity (1) (288 in Zanno et al., 2009; 378 in Turner et al., 2012; 794 in Brusatte et al., 2014). Though stated to be proximal to the entepicondyle by Zanno et al. (2009), it is actually the ectepicondyle as originally noted for *Erliansaurus* in Xu et al. (2002b). It is often called the dorsal supracondylar tubercle in birds.

462. Humerus - angle of torsion between long axes of proximal and distal ends - >32 degrees (0); <33 degrees (1) (289 in Zanno et al., 2009).

463. Humerus - anterior flexure of distal end - significant (0); absent or slight (1) (290 in Zanno et al., 2009; 267 in Turner et al., 2012).

464. Humerus - shape of entepicondyle - hemispherical (0); proximodistally elongate, crest-like (1) (291 in Zanno et al., 2009). Zanno et al. included a composite state for a "poorly developed, absent" entepicondyle, scored as character 297 here.

465. Humerus - anteromedial surface proximal to entepicondyle - flat (0); with longitudinal groove (1) (292 in Zanno et al., 2009).

466. Metacarpal I - ventrolateral surface - unprojected (0); with flange underlying metacarpal II (295 in Zanno et al., 2009; 798 in Brusatte et al., 2014). This eliminates Zanno et al.'s additional qualifier the flange be rectangular.

467. Metacarpal III - convexity of lateral margin - straight or concave (0); convex (1) (299 in Zanno et al., 2009; 445 in Turner et al., 2012).

468. Manual ungual II - length compared to metacarpal II - shorter or equal (0); longer (1) (303 in Zanno et al., 2009; 801 in Brusatte et al., 2014). Zanno et al. compared manual ungual length to penultimate phalanx length for the manus in general, but penultimate phalanx lengths vary independently for each digit and are scored as separate characters here (I-1- 257; II-2- 374; III-3- 380). Instead, we compare ungual II length to metacarpal II and don't include Zanno et al.'s state 2 which is autapomorphic for *Therizinosaurus'* extremely long manual unguals.

469. Ilium - distance between dorsal edge of mid ilium and sacral neural spines - marginal, ilia almost contact neural spines (0); significant (1) (306 in Zanno et al., 2009; 265 in Li et al., 2010; 695 in Brusatte et al., 2014). Zanno et al. distinguish the spacing of therizinosauroids as being greater than other taxa, but this is only true posteriorly and so would also be true of specimens with narrowly separated ilia which have laterally angled postacetabular processes like IGM 100/42.

470. Ilium - lateral surface of posterodorsal corner of postacetabular process - flat (0); with small rugosity (1); with large tuber (2) (ordered) (308 in Zanno et al., 2009; 805 in Brusatte et al., 2014).

471. Ilium - transverse compression of pubic peduncle distal surface - more than twice as long as wide (0); length 50-200% of width (1); less than half as long as wide (2) (ordered) (309 in Zanno et al., 2009). We eliminate the "roughly triangular" qualifier for state 1 found in Zanno et al.'s character.

472. Ilium - angle pubic peduncle distal articular surface projects - >17 degrees anteriorly (0); 17 degrees anteriorly to 9 degrees posteriorly (1); >9 degrees posteriorly (2) (ordered) (301 in Senter et al., 2012a; 310 in Zanno et al., 2009; 806 in Brusatte et al., 2014). We quantify Senter et al.'s states "slanted so that it faces somewhat cranially", "subhorizontal" and "slanted so that it faces somewhat caudally." Zanno et al. have a character involving distal surface orientation, but this is a composite involving peduncle curvature as well.

473. Ilium - ventral extent of antitrochanter - ends proximal to articular surface of ischial peduncle (0); extends to ventral edge of ischial peduncle (1) (312 in Zanno et al., 2009; 807 in Brusatte et al., 2014). This separates Zanno et al.'s composite character scoring for antitrochanter shape (scored as 474 below) and separation from the ischial peduncle.

474. Ilium - shape of antitrochanter - with flattened ventral side (0); hemispherical (1) (312 in Zanno et al., 2009; 807 in Brusatte et al., 2014).

475. Ischium - proximal enclosure of obturator notch - absent (0); present, process on ventral edge of pubic peduncle curves distally (1) (314 in Zanno et al., 2009).

476. Ischium - proximodorsal position of mid dorsal process' apex - <67% down ischium (0); >66% (1) (315 in Zanno et al., 2009; 808 in Brusatte et al., 2014). Zanno et al. formulated this character's states in reference to the position the obturator process, but as that varies independently (character 183 here), we have quantified it compared to ischial length instead.

477. Pubis and ischium - shape of subacetabular contact - straight or simply curved (0); sinuous (1) (316 in Zanno et al., 2009; 810 in Brusatte et al., 2014).

478. Pubis - anteroposterior expansion of distal shaft - less than twice minimum shaft depth (0); at least twice (1) (318 in Zanno et al., 2009; 812 in Brusatte et al., 2014). The shaft's greatest distal width is measured just proximal to where either the anterior or posterior side angles to form the pubic boot.

479. Femur - angle of femoral head to shaft - declined to perpendicular (0); inclined (1) (320 in Zanno et al., 2009; 711 in Brusatte et al., 2014; 855 in Yu et al., 2018). Shaft axis is the average of the entire element, and only the medial portion of the head is used for scoring. Note Zanno et al.'s character formulation doesn't allow scoring taxa with more than a "slight ventral decline." Yu et al. add this character again to Brusatte's matrix, weighting it.

480. Femur - anteroposterior constriction of femoral head in proximal view - at most constricted to 80% of medial head width (0); constricted to <80% of medial head width (1) (321 in Zanno et al., 2009; 813 in Brusatte et al., 2014). The point of constriction is medial to the anterior trochanter, so is inapplicable for taxa without proximally projected anterior trochanters. This quantifies Zanno et al.'s character scoring for constriction.

481. Femur - anteroposterior width of greater trochanter - narrower than or equal to width of femoral head (0); wider (1) (322 in Zanno et al., 2009; 857 in Yu et al., 2018).

482. Femur - ventral constriction of femoral head - present (0); absent (1) (323 in Zanno et al., 2009; 814 in Brusatte et al., 2014).

483. Tibia - depth of incisura tibialis compared to proximal width of tibia - >15% (0); <16% (1) (326 in Zanno et al., 2009; 288 in Li et al., 2010; 815 in Brusatte et al., 2014). This quantifies Zanno et al.'s and Li et al.'s state 1 "wide and shallow, nearly absent."

484. Tibia - transverse compression of proximal end - longer than wide (0); equal or wider than long (1) (327 in Zanno et al., 2009; 816 in Brusatte et al., 2014).

485. Tibia - distal extent of fibular crest - ends at <45% of tibial length (0); >44% (1) (328 and 341 in Zanno et al., 2009; 817 in Brusatte et al., 2014). This measures to the distal edge of the crest. It quantifies the state "long extends to midshaft" from Zanno et al.. Those authors also scored fibular crest length as a separate character, but proximal extent is covered by character 437, so that long crests are also distally extensive crests. The only taxon scored differently for the two characters by Zanno et al. is *Beipiaosaurus*, which was miscoded by them as having a short crest. Thus we follow Brusatte et al. in only using one character.

486. Fibula - angle of proximal edge - <16 degrees from perpendicular to long axis of shaft (0); >15 degrees anteroproximally (1) (329 in Zanno et al., 2009; 774 in Brusatte et al., 2014). Shaft axis is only the proximal portion, to account for curved fibulae. This quantifies Zanno et al.'s state "cranial portion dorsally elevated."

487. Fibula - proximodistal position of iliofibularis tubercle apex - placed <41% down length of fibula (0); >40% (1) (330 in Zanno et al., 2009; 819 in Brusatte et al., 2014). In taxa with reduced fibulae, this is measured compared to the length of the tibia instead. This quantifies Zanno et al.'s state "positioned at midshaft" and covers taxa with more distally placed tubercles.

488. Fibula - transverse width of posterior half in proximal view - equal to or wider than anterior half (0); narrower, forming posteriorly tapered surface (1) (331 in Zanno et al., 2009; 775 in Brusatte et al., 2014). Unlike Zanno et al.'s character formulation, this covers taxa with narrower anterior fibular margins.

489. Tibia - distal exposure of lateral condyle - absent, covered by astragalocalcaneum (0); present due to reduction in astragalus (1) (332 in Zanno et al., 2009; 821 in Brusatte et al., 2014).

490. Femur - convexity of proximal surface between head and greater trochanter - convex to slightly concave (0); strongly concave (1) (339 in Zanno et al., 2009; 711 and 771 in Brusatte et al., 2014). While not quantified here, scores comparable to Zanno's require the concavity be at least as deep as in *Tyrannosaurus* (e.g. Fig. 95 in Brochu, 2003). Note Brusatte et al. score this twice, once in a composite character also scoring head inclination.

491. Maxillary teeth - position of largest teeth - in anterior third (0); in middle third (1); in posterior third (2) (ordered) (340 in Zanno et al., 2009; separation of states 0 and 1 new).

492. Tibia - anterior exposure of mediodistal corner of tibia - covered by astragalus (0); exposed (1) (342 in Zanno et al., 2009; 818 in Brusatte et al., 2014). Zanno et al. included a third state in this character to describe a "cranial tuberosity" of the mediodistal tibia in derived therizinosaurs, but the condition in e.g. *Nothronychus* spp. and *Segnosaurus* does not appear different from most theropods where the mediodistal corner is anteriorly projected.

493. Caudal vertebrae - separation of tip of distal caudal prezygapophyses - unseparated (0); bifurcated (1) (195 in Senter et al., 2012a).

494. Braincase - confluence of otic recess and lateral depression - separate (0); within common depression (1) (344 in Zanno et al., 2009; 459 in Turner et al., 2012).

495. Postorbital and jugal - minimum anteroposterior length of postorbital bar - less than or equal to minimum anteroposterior length of lacrimal (0); greater (1) (347 in Zanno et al., 2009; 268 in Li et al., 2010; 45 in Senter, 2011; 552 in Brusatte et al., 2014). Note Zanno et al.'s state 1 scores for a postorbital bar over twice the length of the lacrimal, but we quantify Li et al.'s "about twice as wide" and Senter's "much broader" qualifiers.

496. Lacrimal - separation from postorbital dorsal to orbit - wide (0); narrow (1); absent (2) (ordered) (348 in Zanno et al., 2009; 579 in Brusatte et al., 2014; states 0 and 1 separated after 271 in Li et al., 2010 and 67 in Senter, 2011).

497. Premaxillary teeth - labiolingual compression - longest axis mesiodistal (0); longest axis labiolingual (1) (91 in Li et al., 2010). Li et al. added this to Norell et al.'s character scoring for lingual convexity in premaxillary teeth, making it a composite and pairing it with the presence of a lingual ridge (here character 498).

498. Premaxillary teeth - lingual surface - flat (0); with central basoapical ridge (1) (91 in Li et al., 2010; 648 in Brusatte et al., 2014). The commentary for character 497 above applies here.

499. Infratemporal fenestra - posterior constriction caused by squamosal and quadratojugal - greatest anteroposterior width of posterior intrusion into fenestra / greatest anteroposterior width of fenestra if protrusion were absent <10% (0); >9% (1) (254 in Li et al., 2010; 252 in Brusatte et al., 2014). This quantifies Li et al.'s state "kinked into laterotemporal fenestra" and eliminates the qualifier the kink is "at midheight." It is equivalent to states 0 vs. 1/2/3 in Brusatte et al.'s (2014) character 252.

500. Nasals - convexity of dorsolateral surface - concave to right-angled (0); sharply convex, forming paired ridges or crests (1) (264 in Li et al., 2010; 512 in Brusatte et al., 2014). Li et al. had this state in a composite character also scoring for median crests (character 404 here), which we separate.

501. Ilium - lateral surface dorsal to acetabulum - flat or slightly rounded (0); with well defined vertical ridge (1) (266 in Li et al., 2010; 301 in Senter, 2011; 259 in Turner et al., 2012).

502. Nasal - lateral surface along or inside antorbital fossa - solid (0); pierced by at least one pneumatic foramen (1) (267 in Li et al., 2010; 30 in Brusatte et al., 2014). This does not code for foramina away from the antorbital fossa, as seen in some oviraptorids. Brusatte et al. added this as a state of their character scoring for the oviraptorid condition, but as they are independent variables that could coexist they are scored as separate characters here. It is coded inapplicable for taxa which lack contact between the nasal and antorbital fenestra.

503. Articular - transverse width of retroarticular process - equal to narrower than mandible anterior to glenoid (0); wider than mandible anterior to glenoid (1) (269 in Li et al., 2010). This eliminates Li et al.'s composite qualifiers of "low" for state 0 and "short" (here character 84) and "with distinct caudally facing concavity" for state 1.

504. Basioccipital and basisphenoid - transverse width across basipterygoid processes - equal to less than anteroposterior length between anterior edge of basipterygoid processes and posterior edge of basal tubera (0); greater (1) (272 in Li et al., 2010; 616 in Brusatte et al., 2014).

505. Ischium - transverse convexity of acetabular surface - convex or flat (0); concave due to sagittal groove (1) (274 in Li et al., 2010). Note Li et al.'s character formulation has no state scoring a flat surface.

506. Paraquadrate foramen - dorsoventral size - <18% of quadrate height (0); >17% (1) (275 in Li et al., 2010; 566 in Brusatte et al., 2014). This quantifies Li et al.'s qualifiers "small" and "very large" and separates lateral quadrate participation (character 507 here) and foramen lateromedial compression. Brusatte et al. used discontinuous quantifiers of "approximately 10%" and "greater than 20%" of quadrate height.

507. Paraquadrate foramen - composition of lateral edge - formed at least partially by quadrate (0); formed completely by quadratojugal (1) (275 in Li et al., 2010; 569 in Brusatte et al., 2014). This separates lateral quadrate participation from Li et al.'s composite variables.

508. Axis - dorsal transverse expansion of neural spine - tapered to equal to base (0); expanded dorsally, resulting in epineurapophyseal processes (1) (277 in Li et al., 2010).

509. Ilium - extent of supracetabular crest along pubic peduncle - present on most of peduncle (0); present on minority of peduncle if at all (1) (278 in Li et al., 2010; 487 in Gianechini et al., 2017; 697 in Brusatte et al., 2014). Even in taxa which lack a convex supracetabular crest, there is often a lip on the acetabulum that this codes for.

510. Scapula - convexity of dorsal acromial edge - convex to straight (0); concave (1) (279 in Li et al., 2010; 749 in Brusatte et al., 2014). As initially worded, this character was a composite of acromial depth and shape. Here, depth is covered by character 402, and the noted difference in shape is the dorsal concavity of the acromial process, even when this process tapers distally (e.g. *Gallimimus*, *Struthiomimus*).

511. Basisphenoid - lateral walls of basisphenoid recess - unmarked (0); have muscle scars (1) (280 in Li et al., 2010; 750 in Brusatte et al., 2014).

512. Quadratojugal - convexity of external surface - flat (0); flexed at posterior border, so that distinct posterior face is present (1) (281 in Li et al., 2010; 564 in Brusatte et al., 2014).

513. Nasal - convexity of lateral edge - convex or straight (0); concave, so that paired nasals are hourglass-shaped (1) (285 in Li et al., 2010; 507 in Brusatte et al., 2014).

514. Metatarsal III - shape of medial edge when articulated, taking into account curvature of shaft - distally concave to slightly convex (0); with deep convexity localized distally (1) (289 in Li et al., 2010; 723 in Brusatte et al., 2014). While not quantified, this doesn't count shallow convexities such as in *Harpymimus* (e.g. compare it to the apomorphic *Garudimimus* and *Gallimimus?* "mongoliensis" in Kobayashi and Barsbold's [2005b] figure 20), or those caused by the entire metatarsal III being curved laterally as in some enantiornithines.

515. Ilium - convexity of pubic peduncle articular surface - convex with angled anterior and posterior components (0); convex and rounded (1); flat or concave (2) (ordered) (291 in Li et al., 2010; 751 in Brusatte et al., 2014). Brusatte et al. removed information from the character by combining states 0 and 1.

516. Ilium - distal anteroposterior expansion of pubic peduncle - unexpanded (0); expanded (1) (293 in Li et al., 2010).

517. Axis - lateral surface of centrum - solid (0); pneumatized by pleurocoel (1) (294 in Li et al., 2010).

518. Maxilla - medial wall of maxillary antrum - solid (0); pneumatized by medial antral fenestra (1) (295 in Li et al., 2010).

519. Surangular - dorsoventral depth at intersection of dentary and external mandibular fenestra - over 50% mandibular depth at that level (0); <51% (1) (71 in Senter, 2010). When no external mandibular fenestra is present, this can be measured at the point of intersection of the dentary, surangular and angular. It is a slightly different measurement than Senter's, which compared surangular depth to that of the "dentary ramus."

520. Pedal ungual II - convexity of posteroventral edge between articular facet and flexor tubercle - convex or straight (0); concave due to notch at base of tubercle (1) (361 in Senter, 2010).

521. Sternum - ossification - unossified in adult (0); ossified (1) (unknown in juveniles) (362 in Senter, 2010; 457 in Turner et al., 2012; 123 in Foth et al., 2014).

522. Pedal digit II - length compared to pedal digit IV, excluding metatarsals and unguals - >79% (0); <80% (1) (364 in Senter, 2010; 254 in Nesbitt et al., 2011). This quantifies Senter's and Nesbitt et al.'s character states.

523. Metatarsal III - transverse medial and lateral bulges on shaft just proximal to articular condyle - absent (0); present (1) (252 in Nesbitt et al., 2011; 476 in Turner et al., 2012). To count as state 1, the bulges must at least partially overlap proximodistally with each other. This separates Norell et al.'s and Turner et al.'s composite character scoring for transverse expansion and dorsal concavity (here character 693). Gianechini et al. (2017) added "partially covering the anterior surface of the metatarsals II and IV" to state 1 of their character 468 "in order to propose a state reflecting more specifically the morphology observed in the taxa used in this analysis", but in the absence of included taxa whose expansions don't cover adjacent metatarsals, this is considered unnecessary here.

524. Metatarsal II - length compared to metatarsal IV - much shorter, <98% of length of metatarsal IV (0); subequal, lengths within 2% of each other (1); much longer, >102% the length of metatarsal IV (2) (ordered) (253 in Nesbitt et al., 2011; 438 in Turner et al., 2012; separation of 1 and 2 new). This quantifies "longer" and "subequal" in Nesbitt et al.'s character, and adds an additional state for metatarsal II longer than IV. Unlike Turner et al.'s character, metatarsal II's state is not dependent on the proximal extent of metatarsal IV's trochlea, which is only scorable in well preserved taxa exposed in ventral view.

525. Metatarsal IV - projection of lateral surface dorsal to lateral flexor ridge on distal end - unprojected (0); with longitudinal ridge (1) (256 in Nesbitt et al., 2011; 477 in Turner et al., 2012). This is only determinable in distal or ventral views.

526. Maxilla - posterior extent of postantral wall - no greater than interfenestral wall (0); extends posterior to interfenestral wall (1) (365 in Agnolin and Novas, 2011; 471 in Gianechini et al., 2017). This is coded inapplicable in taxa which lack a maxillary fenestra, on the assumption those taxa lack a maxillary antrum and thus lack the posteromedial maxillary fenestra which creates separate postantral and interfenestral walls.

527. Teeth - texture of labial and lingual surfaces - smooth (0); fluted basoapically (1) (366 in Agnolin and Novas, 2011; 472 in Gianechini et al., 2017).

528. Dorsal vertebrae - anterior projection of interspinal ligament scars on neural spine - evenly projected along spine (0); more projected dorsally, forming beak-like process (109 in Dal Sasso and Maganuco, 2011).

529. Maxilla - lateral exposure of promaxillary fenestra - covered by lateral lamina of dorsal process (0); at least partially visible laterally (1) (363 in Xu et al., 2011a; 252 in Turner et al., 2012). This scoring is slightly different from Xu et al.'s, which would score "minimal"exposure as state 0.

530. Antorbital fossa - proportions - longer than tall (0); equal to taller than long (1) (364 in Xu et al., 2011a).

531. Jugal - length of posterior process - >15% of total jugal length (0); <16% (1) (366 in Xu et al., 2011a). This quantifies Xu et al.'s state 1 "nearly at the posterior end."

532. Furcula - cross section of omal ends of arms - posteriorly convex to flat (0); posteriorly concave (1) (369 in Xu et al., 2011a; 336 in Turner et al., 2012). Unlike Xu et al.'s formulation, this eliminates terms like "elliptical" and "L-shaped" that also involve convexity of the anterior surface.

533. Cervical vertebrae - post-axial central pneumatization - pleurocoels present (0); pleurocoels absent (1) (106 in Turner et al., 2012).

534. Caudal vertebrae - relationship of zygapophyses - prezygapophyses articulate ventral to postzygapophyses (0); prezygapophyses articulate dorsal to postzygapophyses (1) (120 in Turner et al., 2012). Turner et al. added this state to Norell et al.'s character scoring for distal prezygapophyseal length, which is not only now a composite but also inaccurate since this variable describes proximal caudals.

535. Uncinate processes - articulation to dorsal ribs - unfused (0); fused (1) (unknown in juveniles) (125 in Turner et al., 2012).

536. Ulna - posterior projection of dorsal condyle - unprojected or minimally projected (0); distinctly projected as a semilunate ridge (1) (143 in Turner et al., 2012). Note Turner et al. incorrectly conflate this with the transversely semilunate surface in alvarezsaurid ulnae and thus wrongly merge those characters.

537. Ischium - distal extent of pubic contact - limited to acetabular area and sometimes obturator process (0); extensive, along most of ischial shaft (1) (315 in Senter, 2011; 304 in Makovicky et al., 2012; 177 in Turner et al., 2012). This eliminates the composite specifier of a flat or posteriorly grooved pubis from Makovicky et al.'s formulation. Turner et al. added it to Norell et al.'s character scoring for pubic orientation, but we separate it here as they are different variables.

538. Nasals - transverse convexity of combined dorsal surface - relatively flat to concave (0); strongly convex (1) (253 in Turner et al., 2012). Taxa which have premaxillary-frontal contact are coded inapplicable.

539. Jugal - convexity of anterior tip - convex (0); concave due to bifurcation (1) (262 in Turner et al., 2012). Turner et al. added this state to a characters describing anterior jugal expansion, but we separate it as the two are not independent.

540. Radius - length compared to humerus - <51% (0); 50-90% (1); 91-110% (2); >110% (3) (ordered) (state 0 separated from 268 in Turner et al., 2012; states 1-3 separated from 356 in Turner et al., 2012; 383 in Foth et al., 2014). Turner et al. used two characters describing this concept, radiohumeral ratio more or less than 50% and ulnohumeral ratio <100%, "approximately the same length" or significantly high. We've used the taxon scores to convert ulnohumeral ratios into radiohumeral ratios and combine and quantify the character. Foth et al. used ulnohumeral ratios but lost information by combining our states 2 and 3.

541. Premaxillae - form of contact at median - unfused (0); fused anteriorly but not posteriorly (1); completely fused (2) (ordered) (unknown in juveniles) (269 in Turner et al., 2012).

542. Dentaries - form of contact at median - unfused (0); fused (1) (unknown in juveniles) (270 in Turner et al., 2012).

543. Dentary - anteroventral surface of tip - smooth (0); with groove extending anterolaterally from ventral corner of symphysis (1) (271 in Turner et al., 2012).

544. Premaxilla - length of ventral margin - <91% of orbit+jugal height (0); >90% (1) (272 in Turner et al., 2012). Turner et al. based their character off character 8 of Norell and Clarke (2001), which codes for the portion of snout taken up by premaxilla vs. maxilla. Here it is rephrased as premaxillary length compared to skull size, to match the character coding for maxillary length compared to skull size. When coded this way, Aves is no longer distinct from *Hesperornis*, so Turner's states 1 and 2 are combined into a single state.

545. Premaxilla - length of dorsal process - from tip of snout, extends <61% of preorbital length (0); >60% (1) (273 in Turner et al., 2012). This quantifies Turner et al.'s states "short" and "long, closely approaching frontal."

546. Vomer - extent of contact with pterygoid - broad (0); narrow (1); absent (2) (ordered) (276 in Turner et al., 2012). This eliminates Turner et al.'s extra qualifier for state 1 "pterygoid passes dorsally over palatine."

547. Palatine - extent of contact with pterygoid - long, overlapping (0); short, vertical (1) (277 in Turner et al., 2012).

548. Palatine - contact with premaxilla - absent (0); present (1) (278 in Turner et al., 2012).

549. Vomer - contact with premaxilla - present (0); absent (1) (279 in Turner et al., 2012).

550. Basisphenoid - anteroposterior position of basipterygoid processes - located basally on basisphenoid body (0); located anteriorly on parasphenoid rostrum (1) (282 in Turner et al., 2012). This eliminates Turner et al.'s extra qualifier the rostrum be narrow.

551. Basisphenoid - orientation of articulation with pterygoid - anteroventral (0); mediolateral (1); dorsoventral (2) (unordered) (283 in Turner et al., 2012).

552. Pterygoid - convexity of articulation with basisphenoid - concave (0); flat to low and convex (1); highly convex, forming stalk (2) (ordered) (284 in Turner et al., 2012).

553. Pterygoid - angle between palatal process and posterior end in lateral view - present, posterior end kinked dorsally (0); absent, element straight (1) (285 in Turner et al., 2012).

554. Interorbital septum - ossification - unossified (0); ossified (1) (286 in Turner et al., 2012). Note following Ali et al. (2008), this is a separate, more posterior ossification than the mesethmoid with its median septum.

555. Mesethmoid - anterior extent - barely surpasses frontals if at all (0); extends far anterior to frontals (1) (287 in Turner et al., 2012). This eliminates comparison to posterior extent of the external nares, as narial position and elongation varies between taxa.

556. Basisphenoid - lateromedial position of ossified eustachian tubes - lateral (0); medial but widely separated (1); medial and adjacent or median and confluent (2) (ordered) (288 in Turner et al., 2012).

557. Basisphenoid - ventral surface anterior to basisphenoid recess - solid (0); perforated by paired foramina or median foramen for ossified eustachian tubes (1) (289 in Turner et al., 2012).

558. Squamosal - length of ventral process - projects noticeably ventral to quadrate notch (0); absent (1) (290 in Turner et al., 2012). This eliminates the variable of lateral quadrate head exposure from Turner et al.'s character.

559. Quadrate - form of articulation with pterygoid - pterygoid broadly overlaps medial surface of pterygoid process (0); restricted to anteromedial edge of process or body of quadrate (1) (291 in Turner et al., 2012).

560. Quadrate - position of articulation with pterygoid - extends to tip of pterygoid process (0); extends to pterygoid process, but ends before tip (1); restricted from pterygoid process (2) (ordered) (292 in Turner et al., 2012). Contra Brusatte et al. (2014), this is not considered inapplicable in taxa with a broadly overlapping articulation, as it is hypothetically possible to have a dorsoventrally restricted but mediolaterally overlapping articulation.

561. Quadrate - convexity of pterygoid articulation - flat or slightly convex (0); highly convex (1) (293 in Turner et al., 2012). This is considered inapplicable in taxa with a broadly overlapping articulation.

562. Quadrate - anterolateral surface on dorsal portion of pterygoid process - flat (0); with tubercle (1) (294 in Turner et al., 2012).

563. Quadrate - form of articulation with quadratojugal - planar (0); condylar (1); trochlear (2) (unordered) (395 in Turner et al., 2012; state 2 added for *Heyuannia*).

564. Quadrate - proximal articulation with prootic and/or opisthotic - absent (0); present (1) (296 in Turner et al., 2012).

565. Quadrate - number of distal condyles exposed on posterior edge - two or less (0); at least three, with posterior condyle between lateral and medial condyles (1) (298 in Turner et al., 2012).

566. Articular - pneumaticity - solid (0); pneumatized by diverticulum of the tympanic airsac (1) (302 in Turner et al., 2012).

567. Splenial - anterior extent - does not extend close to mandibular symphysis (0); extends close to, but does not participate in mandibular symphysis (1); participates in mandibular symphysis (2) (ordered) (304 in Turner et al., 2012).

568. Dentaries - transverse convexity of posterior symphysis - concave (0); flat or convex (1) (305 in Turner et al., 2012).

569. Dentaries - dorsal surface of fused symphysis - solid (0); with one or more foramina (1) (306 in Turner et al., 2012). Clarke (2002) notes that contra Turner et al., only taxa with fused symphyses should be scored for this character.

570. Dentaries - arrangement of symphyseal foramina - median (0); paired (1) (307 in Turner et al., 2012).

571. Dentaries - position of symphyseal foramina - on posterior edge of symphysis (0); on dorsal surface of symphysis (1) (308 in Turner et al., 2012).

572. Splenial - medial overlap of Meckelian groove - absent, Meckelian groove exposed (0); present, Meckelian groove hidden (1) (309 in Turner et al., 2012). Contra Turner et al., only taxa whose splenial extend far anteriorly should be coded, as those with restricted splenials cannot fulfill the character.

573. Jugal - contact with postorbital - present (0); absent (1) (44 in Senter, 2011; 311 in Turner et al., 2012).

574. Frontal - contact with parietal - unfused (0); fused (1) (unknown in juveniles) (312 in Turner et al., 2012).

575. Cervical vertebrae - convexity of posterior articular surface - flat or concave (0); convex (1); concave dorsoventrally, convex horizontally (2) (unordered) (101 in Turner et al., 2012). Turner et al. excluded Clarke's character 52 for cervical coely, which distinguished between the partially heterocoelous cervicals of some taxa and the fully heterocoelous cervicals of Aves and others, unlike the TWiG's cervical coely character. Turner (2008) noted it would be changed to only distinguish degrees of heterocoely and coded it this way, but that masks the intermediate character of semi-heterocoelous centra. Here this is resolved by coding anterior and posterior faces separately, which makes this character equivalent to Turner's, but with a convex state added to cover all morphologies.

576. Dorsal vertebrae - size of hypapophyses on dorsals posterior to third - absent or small (0); large on at least some (1) (313 in Turner et al., 2012). Note this can only be coded for taxa whose fourth dorsal is preserved. Gianechini et al. (2017) deleted this believing it to be "very similar" to character 111, but that scores for the anteriormost dorsals while Clarke (2002:403-404) designed this character to score for more posterior dorsals as in many birds.

577. Dorsal vertebrae - number - more than 12 (0); 12 (1); 11 (2); 10 or less (3) (ordered) (314 in Turner et al., 2012; state 0 new to TWiG, separated after 15 in Chiappe, 1995a).

578. Dorsal vertebrae - anteroposterior position of parapophyses - anterior to diapophyses (0); at same level as diapophyses (1) (316 in Turner et al., 2012).

579. Dorsal vertebrae - transverse compression of centra - length less than midlength width (0); length equal to or greater than midlength width (1) (317 in Turner et al., 2012). This quantifies Turner et al.'s states "approximately equal in length" and "length markedly greater" and allows scoring of taxa with much wider centra.

580. Dorsal vertebrae - convexity of lateral centrum surface - convex to slightly depressed (0); highly concave due to deep fossa, which may be dorsoventrally compressed to form a groove (1) (318 in Turner et al., 2012). Turner et al. use Norell and Clarke's (2001) character 58, which has an additional state coding for foramina, but as this is covered by character 114 and the two can coexist (e.g. *Rahonavis*), that state is not used here. Gianechini et al. (2017) deleted this character due to the overlap in characters scoring for foramina, but this leaves no way to score taxa with fossae or grooves unless homology with pleurocoels is presumed.

581. Dorsal vertebrae - connection of adjacent transverse processes - absent, processes separate (0); processes connected by ossified ligaments (1) (319 in Turner et al., 2012). As ossified ligaments are easily lost or broken away, this is only coded for taxa with articulated and undamaged dorsal columns.

582. Dorsal vertebrae - fusion - no dorsals fused together (0); at least two anterior dorsals fused to form notarium (1) (unknown in juveniles) (320 in Turner et al., 2012). All anterior dorsals must be preserved to code this.

583. Sacrum - length of ancestral avepod thirteenth dorsal and first two sacral centra - at least one longer or equal to more anterior and posterior centra (0); all shorter than surrounding centra (1); at least one adjacent centrum also shorter than surrounding centra (2) (ordered) (321 in Turner et al., 2012). Turner et al. based this character on Clarke's (2002) character 62, so also included transverse process length. As Clarke notes though, *Hesperornis* has short transverse processes on these (and all sacral vertebrae) but was still coded plesiomorphic due to centrum length. Since centrum length is thus the deciding factor, only it is coded for here.

584. Caudal vertebrae - length of transverse processes in caudals 1-5 - equal to or longer than centrum width (0); shorter than centrum width (1) (322 in Turner et al., 2012). This quantifies Turner et al.'s states "subequal" and "significantly shorter" and allows scoring taxa with much longer processes. Turner et al. coded almost all non-pygostylians as polymorphic, presumedly because Norell and Clarke's (2001) character 65 it is based on only specified free caudals, and transverse processes decrease in length distally in long-tailed theropods. Yet as Clarke's intention was to compare their length in the proximal caudals of pygostylians, only proximal caudals are coded for here.

585. Caudal vertebrae - number fused into pygostyle - more than five (0); three to five (1); two (2) (ordered) (324 in Turner et al., 2012). While embryology shows numerous caudals form the pygostyle of Aves, this character only codes for how many are obvious in the adult structure, which can also be approximated by its length.

586. Gastralia - ossification - ossified (0); unossified (1) (325 in Turner et al., 2012).

587. Sternum - extent of ventral median keel - restricted to the posterior part (0); extends from close to anterior edge to posterior part (1); restricted to the anterior part (2) (ordered) (327 in Turner et al., 2012).

588. Sternum - pneumatization of dorsal surface - solid (0); invaded by one or more diverticula of the clavicular airsac (1) (328 in Turner et al., 2012).

589. Sternum - pneumatization of lateral edge between sternal rib articulations - solid (0); invaded by diverticula of the clavicular airsac (1) (329 in Turner et al., 2012).

590. Sternum - distance between coracoid sulci - wide (0); narrow (1); sulci overlap (2) (ordered) (330 in Turner et al., 2012).

591. Sternum - number of sternal ribs that contact sternum - two or less (0); three (1); four (2); five (3); six (4); seven or more (5) (ordered) (331 in Turner et al., 2012). States 0 and 1 are newly separated here to code for additional variation. This can be coded in taxa with unossified sterna because it is equal or less than the number of sternal ribs that contact the dorsal ribs, which can be determined by the expanded ends of the latter.

592. Sternum - topology of ventral surface lateral to midline - smooth (0); with longitudinal ridge, an intermuscular line between the m. supracoracoideus and m. pectoralis (1) (332 in Turner et al., 2012).

593. Sternum - shape of posterior edge near midline - unprojected without fenestra (0); with free posteromedial processes (1); with posteromedial processes fused distally with posteromedian process, forming paired fenestrae (ordered) (unknown in juveniles) (333 in Turner et al., 2012). This is ontogenetically variable in that older enantiornithines have posteromedial processes, while younger specimens do not. Young individuals of taxa with posteromedian fenestrae have yet to be described however, so neither state 0 or 1 is assumed to be a potential juvenile morphology for state 2.

594. Clavicles - median fusion - fused to form furcula (0); unfused (1) (unknown in juveniles) (334 in Turner et al., 2012).

595. Furcula - shape of omal tips in dorsal/ventral view - flat to rounded (0); pointed (1) (337 in Turner et al., 2012).

596. Furcula - shape of ventral edge - convex (0); flat to concave (1) (338 in Turner et al., 2012).

597. Scapulocoracoid - convexity of mobile articulation - concave on scapula, convex on coracoid (0); flat on both bones (1); convex on scapula, concave on coracoid (2) (ordered) (339 in Turner et al., 2012).

598. Coracoid - topology of anterior (medial in Aves) edge near level of scapular articulation - unprojected (0); with procoracoid process (1) (340 in Turner et al., 2012).

599. Coracoid - convexity of posterior (lateral in Aves) margin of distal portion - concave or straight (0); convex (1) (341 in Turner et al., 2012). Note Turner et al.'s states do not allow scoring taxa with extremely concave edges.

600. Coracoid - pneumatization of proximal area, near scapular articulation or coracoid tuber - solid (0); invaded by diverticula of the clavicular airsac (1) (343 and 344 in Turner et al., 2012). Clarke (2002) noted some taxa like *Nothura* have both proximal and distal pneumatic foramina in their coracoids, making them non-homologous. They are thus separated here, which also enables coding of taxa with incompletely preserved or exposed coracoids. Clarke also expressed reservations about the homology of foramina in *Lithornis* (in the acrocoracoid process) and *Crypturellus* (below the scapular cotyla), but a single character for proximal pneumatization is provisionally retained here.

601. Coracoid - pneumatization of mediodistal (posterodistal in Aves) area, near sternal articulation - solid (0); invaded by diverticula of the clavicular airsac (1) (343 and 344 in Turner et al., 2012).

602. Coracoid - shape of posterior (lateral in Aves) edge just proximal to sternal articulation - confluent with more proximal part of edge (0); projected into lateral process (1) (345 in Turner et al., 2012).

603. Coracoid - topology of posterior area of lateral surface (lateral area of anterior surface in Aves) - smooth (0); with ridge extending distally from coracoid tuber to sternal articulation, an intermuscular line between origins of the m. supracoracoideus and m. coracobrachialis posterior (1) (346 in Turner et al., 2012). Note this is not homologous to the elongate biceps ridge in e.g. megaraptorids and *Patagonykus* (character 696), though they are topologically similar.

604. Coracoid - shape of acrocoracoid process - straight or curved laterally (0); curved medially (1) (348 in Turner et al., 2012). Note Turner et al.'s states do not allow scoring taxa with laterally curved acrocoracoid processes.

605. Coracoid - position of supracoracoid nerve - passes through coracoid, resulting in coracoid foramen (0); does not penetrate coracoid, coracoid foramen absent (1) (349 in Turner et al., 2012; 683 in Brusatte et al., 2014).

606. Coracoid - convexity of external surface surrounding coracoid foramen - concave (0); flat or convex (1) (350 in Turner et al., 2012).

607. Coracoid - size of portion proximal to biceps tuber - present, forming distinct protrusion or area of the bone (0); absent, biceps tuber at same level as scapular articulation (1) (351 in Turner et al., 2012). This character is usually phrased as ornithothoracines having an acute angle between the scapula and coracoid, but this is not strictly the difference in question. Maniraptorans have a coracoid which is bent at the level of the biceps tuber, and the angle between it and the scapula is generally measured using the portion proximal to the tuber. Ornithothoracines appear to have a lower angle between their scapula and coracoid, but this is only because they have reduced the area proximal to the coracoid tuber, so their entire coracoid is homologous to the distal coracoid of more basal theropods. For example, the angle between the scapula and proximal coracoid in *Archaeopteryx* is 128 degrees, though the bend causes the angle between the scapula and coracoid distal to the tuber to be 54 degrees. The latter is comparable to ornithothoracines.

608. Scapula - curvature of shaft in lateral view - straight or dorsally curved (0); ventrally curved (1) (353 in Turner et al., 2012).

609. Scapula - curvature of acromion - lateromedially straight or medially curved (0); anterior portion curved laterally (1) (355 in Turner et al., 2012).

610. Humerus - convexity of proximal edge of humeral head when viewed perpendicular to long axis - concave to flat (0); convex (1) (357 in Turner et al., 2012). Note Turner et al.'s states have no way to score taxa with concave proximal edges, while Brusatte et al.'s version (their character 352) allows "weakly convex" edges to be scored state 0.

611. Humerus - closure of capital groove - groove open (0); groove closed (1) (360 in Turner et al., 2012).

612. Humerus - convexity of anterior edge of head in proximal view - straight to convex (0); concave (1) (361 in Turner et al., 2012).

613. Humerus - separation of head from more distal humerus on anterior surface - confluent (0); separated by transverse groove for acrocoracohumeral ligament (1) (362 in Turner et al., 2012).

614. Humerus - orientation of deltopectoral crest in proximal view - in line with long axis of humeral head (0); angled from long axis of humeral head (1) (363 in Turner et al., 2012).

615. Humerus - convexity of proximolateral/posterior surface of deltopectoral crest - convex or flat (0); concave (1) (365 in Turner et al., 2012).

616. Humerus - surface of deltopectoral crest - solid (0); perforated by fenestra (1) (unknown in juveniles) (366 in Turner et al., 2012). We eliminate the qualifier "large" from Turner et al.'s state 1.

617. Humerus - surface of bicipital crest - smooth (0); scar or fossa for m. scapulohumeralis posterior (1) (367 in Turner et al., 2012).

618. Humerus - position of m. scapulohumeralis posterior insertion - medial (anterior in Aves) surface of bicipital crest (0); distal surface of bicipital crest (1); lateral (posterior in Aves) surface of bicipital crest (2) (ordered) (368 in Turner et al., 2012).

619. Humerus - medial (anterior in Aves) projection of bicipital crest - absent (0); moderate (1); highly convex (2) (ordered) (369 in Turner et al., 2012).

620. Humerus - proximal pneumaticity - solid (0); invaded by diverticulum of the clavicular airsac near base of lateral (posterior in Aves) surface of bicipital crest (1) (370 in Turner et al., 2012). We specify the pneumotricipital area since this is what is most often meant when a pneumatic foramen on the humerus is noted (Baumel and Witmer, 1993:99).

621. Humerus - orientation of ectocondyle - mostly proximodistal (0); mostly transverse (1) (4372 in Turner et al., 2012).

622. Humerus - shape of entocondyle - rounded (0); ridge-like (1) (373 in Turner et al., 2012). Note Brusatte et al.'s (2014) version of this character (their 368) scores for both condyles.

623. Humerus - angle of distal edge - perpendicular to shaft or angled so that ectocondyle is further distal (0); angled so that entocondyle is further distal (1) (374 in Turner et al., 2012). This quantifies Turner et al.'s states of "approximately perpendicular" and "projected significantly distal" and allows scoring of taxa whose ectocondyles are further distal.

624. Humerus - anterior surface just proximal to condyles - flat (0); concave for origin of M. brachialis inferior (1) (376 in Turner et al., 2012). Note that unlike Turner et al.'s version, a flat scar would not be scored as state 1.

625. Humerus - size of entocondyle - long axis shorter than long axis of ectocondyle (0); equal or longer (1) (377 in Turner et al., 2012).

626. Humerus - lateral side of posterior surface on distal end - flat (0); with longitudinal groove for passage of m. scapulotriceps (1) (379 in Turner et al., 2012).

627. Humerus - medial side of posterior surface on distal end - flat (0); with longitudinal groove for passage of m. humerotricipitalis, confluent with olecranon fossa (1) (380 in Turner et al., 2012).

628. Ulna - separation of lateral and medial cotylae - confluent or only separated by ridge (0); separated by groove (1) (381 in Turner et al., 2012).

629. Ulna - convexity of lateral cotyla - concave or flat (0); convex (1) (382 in Turner et al., 2012).

630. Ulna - anteroproximal surface - flat (0); scar for the insertion of the M. biceps brachii (1); tubercle for the insertion of the M. biceps brachii (2) (ordered) (384 in Turner et al., 2012).

631. Ulna - anteromedial surface of proximal end - flat (0); scar for insertion of m. brachialis (1) (385 in Turner et al., 2012).

632. Radius - posterolateral surface - flat (0); with long scar for m. pronator teres (1); with long groove (2) (unordered) (386 in Turner et al., 2012).

633. Ulnare - length of medial (ventral) ramus - shorter than lateral ramus (0); subequal to lateral ramus (1); longer than lateral ramus (2) (ordered) (388 in Turner et al., 2012).

634. Metacarpal III - distal contact with metacarpal II - absent, contacting or sutured (0); fused (1) (unknown in juveniles) (390 in Turner et al., 2012).

635. Metacarpal III - minimum transverse width compared to minimum transverse width of metacarpal II - >49% (0); <50% (1) (391 in Turner et al., 2012).

636. Metacarpal I - development of medial process for insertion of m. extensor metacarpi ulnaris - unprojected or slightly convex (0); greatest length of extensor process (measured medially from proximal metacarpal articular surface and lateralmost part of medial edge of metacarpal I) 8-20% of transverse width between medial edge of distal articular surface of metacarpal I and lateral edge of proximal carpometacarpus (1); 21-30% (2); >30% (3) (ordered) (392 in Turner et al., 2012). Turner et al. based their character on 142 of Norell and Clarke (2001), but their measurement uses strictly medial projection compared to the distal articular surface of metacarpal I. As the extensor process is often angled proximally and the distal articular surface difficult to measure in derived birds, the current measurement system is seen as more useful, though it has one less state than Turner et al.'s, as *Limenavis* and *Lithornis* are not intermediate between *Yixianornis* and *Apsaravis* on one hand, and *Iaceornis* and remaining Aves on the other.

637. Metacarpal I - proximodistal convexity of medial edge - concave or straight (0); convex (1) (393 in Turner et al., 2012).

638. Metacarpal II - proximal part of ventral surface - flat (0); pisiform process for retinaculum flexorum (1) (394 in Turner et al., 2012).

639. Distal carpal I - ventral surface - flat (0); with infratrochlear fossa for ventral radiocarpometacarpal ligament (1) (395 in Turner et al., 2012).

640. Metacarpal III - distal extent - proximal to or subequal to metacarpal II (0); distal to metacarpal II (1) (397 in Turner et al., 2012).

641. Metacarpal II - dorsolateral surface near middle of shaft - smooth (0); scar for insertion of m. extensor carpi ulnaris longus (1); intermetacarpal tubercle or process for insertion of m. extensor carpi ulnaris longus (2) (ordered) (398 in Turner et al., 2012).

642. Manual phalanx II-1 - projection of laterodistal margin - does not extend distally past articular surface (0); extends past surface as internal index process (1) (401 in Turner et al., 2012).

643. Pubis - contact with ilium - articulated (0); fused (1) (unknown in juveniles) (402 in Turner et al., 2012). While Turner et al. based their character off 153 of Norell and Clarke (2001) which had a single character for pelvic fusion (with three states, the intermediate coding for only ilioischial fusion), each articular surface is coded separately here to better express the variation and amount of uncertainty in incomplete taxa.

644. Ischium - contact with ilium - articulated (0); fused (1) (unknown in juveniles) (402 in Turner et al., 2012).

645. Pubis - contact with ischium - articulated (0); fused (1) (unknown in juveniles) (402 in Turner et al., 2012).

646. Ischium - contact between mid dorsal process and ilium - absent (0); present (1) (403 in Turner et al., 2012).

647. Ischium - contact between proximodorsal process and ilium - absent (0); present (1) (404 in Turner et al., 2012).

648. Ilium - position of antitrochanter - mostly posterior to acetabulum (0); mostly dorsal to acetabulum (1) (405 in Turner et al., 2012). This can be measured by making horizontal and vertical lines from the dorsal and posterior acetabular edges respectively.

649. Pelvis - development of pubic tubercle on anterolateral surface of proximal pubis or pubic peduncle of ilium - undeveloped or a scar (0); process for attachment of inguinal ligament, M. obliquus abdominus and/or M. ambiens (1) (406 in Turner et al., 2012; 307 in Senter et al., 2012a; 702 in Brusatte et al., 2014; 364 in van der Reest and Currie, 2017). As this only shifts to the ilium in neognaths, non-neognath taxa are scored based on their pubis. Turner et al. based this character on 157 of Norell and Clarke (2001), which distinguished between 'small' and 'well projected' flanges. Here these states are joined, as taxa with supposedly small flanges (e.g. *Fumicollis* [their *Baptornis*] with a process 11% of preacetabular length; *Hesperornis* at 9%) show no difference from taxa with supposedly well-developed flanges (e.g. *Lithornis* at 5-8%; *Gallus* at 11%).

650. Ilia - contact between dorsal edges of preacetabular processes or between dorsal edges and sacral neural spines - absent or freely contacting (0); fused (1) (unknown in juveniles) (407 in Turner et al., 2012). This can sometimes be determined by ilial height and sacral width, if regardless of ilial angle median contact was impossible.

651. Ilium - overlap with last dorsal rib - absent (0); present (1) (408 in Turner et al., 2012).

652. Ilium - orientation of postacetabular process in section - mostly vertical (0); mostly horizontal (1) (409 in Turner et al., 2012).

653. Ilium - internal surface of postacetabular process - flat (0); with renal fossa for posterior portion of kidney (1) (410 in Turner et al., 2012).

654. Pubis - transverse compression of midshaft - anteroposteriorly compressed or equal (0); transversely compressed (317 in Zanno et al., 2009; 412 in Turner et al., 2012; 811 in Brusatte et al., 2014).

655. Femur - convexity of anterior edge of distal end - convex to slightly concave (0); markedly concave, forming deep extensor or patellar groove (1) (415 in Turner et al., 2012; 712 in Brusatte et al., 2014). Brusatte et al. do not believe extensor and patellar grooves to be homologous, so code them separately, but distinguishing them requires either a preserved patella, a very well preserved specimen, or assumptions of phylogeny. They are thus not distinguished here.

656. Femur - confluence between lateral condyle and ectocondylar tuber - separated by notch (0); joined into tibiofibular crest (1) (416 in Turner et al., 2012).

657. Femur - posterior projection of posterior surface just proximal to lateral condyle - in line with medial surface (0); distinctly more posteriorly projected than medial surface (1) (417 in Turner et al., 2012).

658. Femur - transverse position of lateral edge of ectocondylar tuber or tibiofibular crest - far medial to lateral edge of lateral condyle, <90% of distance across condyles, measured from medial edge (0); roughly in line with lateral edge of lateral condyle, 90-100% of distance across condyles (1); lateral to lateral edge of lateral condyle, >100% of distance across condyles (2) (ordered) (418 in Turner et al., 2012). This quantifies the "laterally projected fibular trochlea" of Turner et al.'s character.

659. Astragalocalcaneum - anterior projection of medial condyle - much greater than lateral condyle (0); slightly greater to less than lateral condyle (measuring from a line across the posterior tip of each condyle, medial condyle extends anteriorly <110% as much as lateral condyle) (1) (419 in Turner et al., 2012). This quantifies the "medial condyle projecting further anteriorly" in Turner et al.'s character, and allows scoring taxa whose lateral condyles project further.

660. Tibiotarsus - lateral side of anterior surface just proximal to distal condyles - flat (0); with open longitudinal extensor canal for ligament of m. extensor digitorum longus (1); ossified ligament crossing extensor canal to form supratendinal bridge (2) (ordered) (420 in Turner et al., 2012).

661. Astragalus - anterior surface of ascending process - concave or flat (0); with tubercle for attachment of retinaculum bridging m. tibialis cranialis and m. digitorum longus (1) (421 in Turner et al., 2012).

662. Astragalocalcaneum - transverse width of medial condyle - >110% transverse width of lateral condyle (measured from point of greatest constriction between condyles) (0); 110-90% (1); <90% (2) (ordered) (422 in Turner et al., 2012). This quantifies the "wider" and "approximately equal" states of Turner et al.'s character.

663. Astragalocalcaneum - convexity of anterior edge of medial condyle - highly convex (0); straight or almost straight (1) (423 and 424 in Turner et al., 2012). Turner et al. based these characters off 183 and 184 in Norell and Clarke (2001). Character 423 coded for condyles which did not slope inward in distal view, while character 424 coded for a narrow intercondylar groove. In actuality, *Vorona* and some enantiornithines like *Nanantius* have a tibiotarsus where only the wide medial condyle is minimally sloped until its lateralmost portion. The lateral condyle is still highly sloped (contra Norell and Clarke's character wording), but is so narrow that when combined with the lateralmost highly sloping portion of the medial condyle, it forms a narrow intercondylar groove. As narrow lateral condyles are coded by another character, only the condition of the medial condyle is coded for in this character. Notably, *Apsaravis* has been coded the same way by Norell and Clarke, but its situation is the opposite- its medial condyle is narrow and highly sloped, while the wide lateral condyle lacks an inward slope until its medialmost portion. Thus its morphology is not homologous.

664. Tibia - proximal extent of trochlea for the tibial cartilage on the posterior surface of the distal end - proximal extent less than 51% of distal tibiotarsal transverse width (0); >50% (1) (425 in Turner et al., 2012). This quantifies Turner et al.'s "well-developed posterior extension" for state 1.

665. Tibia - definition of lateral and medial edges of trochlea for the tibial cartilage on the posterior surface of the distal end - undifferentiated from adjacent bone or low rounded ridges (0); defined by sharp crests continuous with distal condyles (1) (425 in Turner et al., 2012).

666. Tibia - distal transverse expansion - transverse width just proximal to distal condyles >150% of midshaft transverse width (0); <151% (1) (426 in Turner et al., 2012). This quantifies Turner et al.'s "approximately equal to shaft width" qualifier for state 1.

667. Metatarsal V - presence - present (0); absent (1) (427 in Turner et al., 2012; 300 in Foth et al., 2014).

668. Tarsometatarsus - proximal surface - flat (0); with central raised intercotylar eminence (1) (429 in Turner et al., 2012). Note this cannot be evaluated in taxa with unfused metatarsals unless distal tarsals are known. This eliminates Turner et al.'s extra qualifier of "globose" for the eminence.

669. Tarsometatarsus - proximal part of posterior surface - flat (0); raised hypotarsus with flat posterior surface (0); raised hypotarsus with grooved posterior surface for flexor tendons (2); raised hypotarsus with perforated posterior surface for flexor tendons (3) (ordered) (430 in Turner et al., 2012). While usually assumed to be absent in non-bird taxa with unfused metatarsi, the condition in e.g. *Gallimimus bullatus* (Osmolska et al., 1972- Fig. 16A2) would seem to qualify as state 1.

670. Metatarsus - dorsal and ventral surface near proximal end between metatarsals III and IV - solid (0); with vascular foramen (1) (431 in Turner et al., 2012). Due to the ambiguity of suture width between metatarsals, this and the following character are only coded for taxa with proximally fused metatarsi.

671. Metatarsus - dorsal and ventral surface near proximal end between metatarsals II and III - solid (0); with vascular foramen (1) (431 in Turner et al., 2012). This is separated from character 670 unlike in Turner et al.'s list where they formed an ordered sequence, as the condition of only having a foramen between II and III is logically possible.

672. Metatarsal I - posterior curvature - absent, shaft straight in medial view (0); present, shaft J-shaped (1) (432 in Turner et al., 2012). This is separated from Turner et al.'s composite character also scoring for metatarsal torsion (character 389 above).

673. Metatarsal II - surface for articulation of metatarsal I - flat or convex (0); slightly concave (1); well developed fossa (2) (ordered) (433 in Turner et al., 2012).

674. Metatarsal II - transverse width of trochlea - <80% of metatarsal III trochlea (0); 80-130% of metatarsal III trochlea (1); >130% of metatarsal III trochlea (2) (ordered) (435 in Turner et al., 2012; 836 in Brusatte et al., 2014). Turner et al.'s version of this character is an extreme composite, with three states scoring for trochlea II width compared to trochlea "III and/or IV", which would not guarantee homology if one OTU qualifies compared to III and another qualifies compared to IV. Their new fourth state is "IV narrowest" which could also be true for their state 1. Instead, we compare II and IV separately to III here and quantify the states. Brusatte et al. also have a composite state of the widest of metatarsal II or IV <77% of metatarsal III in trochlear width, which does not guarantee homologous conditions if one taxon has mtII wider than IV and another taxon has the opposite.

675. Metatarsal IV - transverse width of trochlea - <60% of metatarsal III trochlea (0); 60-200% of metatarsal III trochlea (1); >200% of metatarsal III trochlea (2) (ordered) (435 in Turner et al., 2012; 836 in Brusatte et al., 2014). The commentary for character 674 applies here.

676. Metatarsus - distal surface between fused metatarsals III and IV - solid (0); with vascular foramen (1) (436 in Turner et al., 2012). Note this is inapplicable in taxa that lack a distally fused vascular foramen between metatarsals III and IV (state 3 for character 216).

677. Metatarsal III - proximal extent of lateral trochlear edge on posterior surface - less than or equal to medial trochlear edge (0); greater than medial trochlear edge (1) (437 in Turner et al., 2012). This is scored inapplicable in taxa which have a single, central proximally extended trochlear surface instead of a bifurcated one.

678. Chevrons - length of posterodistal process on distal caudals - shorter than centrum (0); longer than centrum (1) (442 in Turner et al., 2012).

679. Exoccipital - length of metotic strut, measured from base of paroccipital process to tip of basal tuber - <35% of height from tip of basal tuber to skull roof (0); >34% (1) (449 in Turner et al., 2012). This quantifies Turner et al.s' states of "short and robust" and "long and narrow."

680. Basisphenoid - lateral surface ventral to trigeminal (V) nerve foramen and anteroventral to otic recess - flat (0); excavated by main portion of anterior tympanic recess (1) (451 in Turner et al., 2012; 852 in Brusatte et al., 2014).

681. Basisphenoid - position of anterior tympanic recess - extends posterior to basipterygoid processes (0); restricted to above or anterior to basipterygoid processes (1) (452 in Turner et al., 2012).

682. Pedal phalanx II-2 - height of distal end - greater than 79% height of proximal end (0); less than 80% (1) (456 in Turner et al., 2012). This more accurately quantifies Turner et al.'s state "distal surface less than half the size of proximal surface" to correspond to their taxon scores.

683. Ulna - size of lateral cotyla - smaller than medial cotyla (0); equal or larger than medial cotyla (1) (458 in Turner et al., 2012). Note Turner et al.'s states do not allow scoring taxa with larger lateral cotylae.

684. Quadratojugal - size - length of ventral margin >29% of jugal+orbit height (0); <30% (1) (462 in Turner et al., 2012). This quantifies Turner et al.'s "greatly reduced" qualifier for state 1.

685. Frontal - surface of articulation for postorbital - smooth or slightly notched (0); deeply notched (1) (463 in Turner et al., 2012).

686. Furcula - cross section of center - compressed dorsoventrally to slightly compressed anteroposteriorly (0); highly compressed anteroposteriorly (1) (469 in Turner et al., 2012). Turner et al.'s states lack a way to score taxa with dorsoventrally compressed furculae.

687. Furcula - width of epicleidial ends - equal or narrower than arms (0); broader than arms (1) (471 in Turner et al., 2012).

688. Furcula - width of arms - equal or narrower than center and epicleidia (0); broader than center and epicleidia (2) (472 in Turner et al., 2012).

689. Furcula - anterior surface of hypocleidium - flat to rounded (0); keeled (1) (473 in Turner et al., 2012).

690. Furcula - symmetry - asymmetrical (0); nearly symmetrical (1) (474 in Turner et al., 2012).

691. Furcula - robusticity - width halfway down arms <20% of arm length (0); >19% (1) (475 in Turner et al., 2012). This quantifies Turner et al.'s "thin" and "thick" states.

692. Distal caudal vertebrae - separation of postzygapophyses - separated posterior to centrum (0); joined by lamina past centrum (1) (new).

693. Metatarsal III - dorsal surface proximal to articular area and extensor fossa - flat to convex (0); concave forming fossa (1) (252 in Nesbitt et al., 2011; 476 in Turner et al., 2012; 366 in van der Reest and Currie, 2017). As noted above, this separates transverse expansion from dorsal concavity (character 523 here) in the original character.

694. Astragalocalcaneum - proximodistal height of lateral condyle - <115% height of medial condyle (0); >114% height of medial condyle (1) (297 in Makovicky et al., 2012). This quantifies "markedly taller" in Makovicky et al.'s character.

695. Astragalus - anterior surface at base of ascending process - without pitting (0); one or more pits present (1) (299 in Makovicky et al., 2012; 776 in Brusatte et al., 2014).

696. Coracoid - lateral (anterior in Aves) surface for origin of M. biceps brachii - convex tuber (0); convex ridge (1); flat (2) (unordered) (301 in Makovicky et al., 2012; 760 in Brusatte et al., 2014).

697. Manual ungual I - enclosure of proximoventral vascular grooves - open (0); enclosed forming proximoventral foramina (1) (770 in Brusatte et al., 2014).

698. Maxilla - lateral surface ventral to antorbital fossa - flat (0); horizontal ridge present (1) (501 in Brusatte et al., 2014).

699. Pubis - abruptness of pubic boot anteroposterior expansion - abrupt (0); gradual over distal portion of pubis (1) (313 in Senter, 2011).

700. Astragalus - anterior surface of center of body - proximodistally convex to flat (0); with transverse groove (1) (300 in Makovicky et al., 2012; 777 in Brusatte et al., 2014).

**Excluded characters**

Skull, anteroposterior length: 0: less than 40% trunk length 1: greater than 40% trunk length (478 in Brusatte et al., 2014). This is correlated with craniofemoral ratios (character 337) and dorsofemoral ratios (character 243).

Length of preorbital region of cranium < 3x height of orbit (0) or > 3x height of orbit (3 in Senter, 2011). This is correlated with maxillary and premaxillary length, each of which are already represented in the matrix (characters 346 and 544).

Antorbital fenestra, size relative to external naris: larger (0) or smaller (1) (365 in Xu et al., 2011a). This is correlated to naris size and maxillary length, both of which are coded by other characters (characters 233 and 346).

Antorbital fenestra large (0) or small (1) relative to orbit (5 in Xu, 2002; 5 in Xu and Zhang, 2005; 236 in Senter, 2007). This is correlated with maxillary length, orbit shape and the amount of the antorbital fossa covered by the antorbital fenestra, all of which are coded by other characters (characters 346, 8 and 339).

Maxilla, maxillary fenestra, anteroposterior length compared to the distance between the anterior margins of the antorbital fossa and fenestra: (ORDERED) 0: less than half 1: greater than half 2: greater than half and also greater than half of the length of the eyeball-bearing portion of the orbit (488 in Brusatte et al., 2014). This is correlated with maxillary fenestra size (character 4), antorbital fossa wall length (character 339) and orbital length (character 8).

Maxilla, interfenestral strut, anteroposterior length: 0: greater than 50% of long axis of maxillary fenestra 1: less than 50% of long axis of maxillary fenestra (491 in Brusatte et al., 2014). This is correlated with maxillary fenestra size (character 4), antorbital fossa wall length (character 339) and maxillary fenestra anteroposterior position (character 6).

Lacrimal without dorsal wing (0), or dorsal wing wrapping onto side of nasal crest (1) (290 in Li et al., 2010). This character was added by Li et al., though only present in their outgroup *Dilophosaurus* and *Monolophosaurus* in the matrix. As this is topologically the same as a combination of lacrimal horn (character 41) and nasal crests (characters 404 and 500 for median and paired crests respectively), the character is excluded.

Lacrimal posterodorsal process absent (inverted ‘L’ shaped) (0) or lacrimal ‘T’ shaped in lateral view (1) or anterodorsal process much longer than posterior process (2) or posterodorsal process subvertical (3) (40 in Senter, 2007). Lacrimal, posterodorsal process, orientation: subvertical (0) or posteriorly inclined (1) (372 in Xu et al., 2011a). Senter coded state 3 as present in *Caudipteryx* and oviraptorids, while Xu et al. also coded scansoriopterygids and *Sapeornis* this way. However, most examined taxa have dorsally angled posterior processes when compared to the ventral process, with dromaeosaurids averaging 110 degrees for example. The character might be defined more strictly to reflect the higher values of some oviraptorosaurs (*Similicaudipteryx*- 124 degrees; *Caudipteryx*- 132 degrees) and *Epidexipteryx* (148 degrees), but this is also matched by less closely related taxa such as *Haplocheirus* (147 degrees) and *Zuolong* (131 degrees), and is not found in *Incisivosaurus* (102 degrees) or *Scansoriopteryx* (93 degrees). *Sapeornis* can have both states. Given this data, if the character is reinstated, it will not have the power it does in Xu et al.'s matrix, where all taxa except oviraptorosaurs and paravians were also inexplicably coded as inapplicable.

Suborbital process of jugal dorsoventrally stout, and not as in state 2 (0) or elongate and dorsoventrally narrow (1) or dorsoventrally stout, with caudal end dorsoventrally taller than cranial end, so that orbital margin slopes downward cranially (2) (50 in Senter, 2011). States 0/2 vs. 1 combine orbital length (character 8) and jugal height ventral to orbit (character 343), while states 0 vs. 2 would score for anterior jugal dorsoventral expansion (character 399) although most taxa scored as 2 by Senter actually have state 0.

Position of frontoparietal suture relative to postorbital processes of frontal 0: well posterior to the postorbital processes 1: at the level of the postorbital processes 2: anterior to postorbital processes (464 in Turner et al., 2012). Originally highly homoplasic in Turner's matrix with the exception of only oviraptorids having state 2. To be informative, this would need to specify which lateromedial portion of the suture was being measured, and how much anteroposterior variance is necessary to not fall into state 1. Until this is done it it rejected as too subjective.

Supratemporal fossa with limited extension onto dorsal surfaces of frontal and postorbital (0) or covers most of frontal process of the postorbital and extends anteriorly onto dorsal surface of frontal (1) (245 in Turner et al., 2007). This is difficult to code objectively due to the widely varying lengths and widths of theropod frontals, as well as the irregular width of the fossa along a frontal's posterolateral margin. Furthermore, most small taxa have indistinct fossa margins which might suggest state 0, but when well preserved are often extensive (e.g. *Archaeopteryx* neotype NHMUK 37001 in Domínguez Alonso et al. [2004]). Until more objective coding is possible, this character is rejected.

Supratemporal fenestra bounded laterally and posteriorly by the squamosal (0) or supratemporal fenestra extended as a fossa on to the dorsal surface of the squamosal (1) (216 in Hwang et al., 2004). Most taxa with well preserved squamosals visible in dorsal view have some lateral extension of the supratemporal fossa, contra codings in most matrices.

Quadrate, cluster of pneumatic foramina on posterior surface of the tip of dorsal process 0: absent 1: present (300 in Turner et al., 2012). This is a subset of character 60, and seemingly a synapomorphy of crown palaeognaths, represented by one taxon each in our and Turner et al.'s matrices.

Maxillary tooth height highly variable with gaps evident for replacement (0) or almost isodont with no replacement gaps (1) (249 in Turner et al., 2007b). Replacement, shed and missing teeth make this difficult to code, and most taxa coded as apomorphic by Turner et al. actually do have at least one adjacent pair of teeth differing in height by >30%.

Middle of maxillary tooth row, spacing between teeth: 0: narrow, teeth separated by less than one crown width. 1: wide, adjacent teeth separated by a gap corresponding to one crown width or more (492 in Gianechini et al., 2017). State 1 is scored in *Buitreraptor*, *Archaeopteryx* and *Anchiornis*. The first only preserves four maxillary teeth, with two of the three gaps clearly being longer than the FABL. *Archaeopteryx* is variable, with no interdental spaces qualifying in the twelfth specimen and at least some in the tenth specimen for instance. Similarly, no spaces of *Anchiornis* specimen LPM-B00169 count whereas PKUP V1068 has some that do and others that don't. Given the variability between specimens and within the same tooth row, the character is not used here.

Maxillary teeth almost perpendicular to jaw margin (0) or inclined strongly posteroventrally(1) (248 in Turner et al., 2007b; 259 in Senter, 2007). Senter (2010) showed this is a taphonomic artifact in *Bambiraptor* and *Deinonychus*.

Maxillary and dentary teeth labiolingually flattened and recurved, with crowns in middle of tooth row more than twice as high as the basal mesiolateral width (0) or lanceolate and subsymmetrical (1) or conical (2) or labiolingually flattened and recurved, with crowns in middle of tooth row less than twice as high as the basal mesiolateral width (fore–aft basal length) (3) (230 in Senter, 2007). State 3 refers to crown height compared to FABL, but no examined taxon has all teeth over twice as tall as mesiodistally long, with basically every taxon being polymorphic. While some taxa such as therizinosaurs and derived troodontids may have all teeth apicobasally short, a character needing basically all teeth present to code would be coded unknown in the vast majority of taxa.

Basipterygoid processes well developed and ... (0) anteroposteriorly short and finger-like (approximately as long as wide) (1) longer than wide (2) significantly elongated and tapering (new to TWG, 99 in Choiniere et al., 2010). State 2 was proposed to unite *Haplocheirus* and *Shuvuuia*, and could potentially be used as a new state for our character 22 scoring basipterygoid process length. However, this is hindered by the process in *Haplocheirus* only being visible extending into the matrix in posterior view (Choiniere et al., 2014: Fig. 7B, D) and as a tip emerging below the quadrate in lateral view (Fig. 4). In *Shuvuuia*, while Chiappe et al. (1998:277) state "the basipterygoid processes are unusually long" and Chiappe et al. (2002:97) say "the proportional length of the basipterygoid processes in *Shuvuuia* is unparalleled by any other bird or nonavian theropod", Dufeau (2003: Fig. 24) shows a process in lateral view no longer thane.g. *Tsaagan*'s (Norell et al., 2006: Fig. 3C). Similarly, *Haplocheirus*' vertical distance from ventral cultriform process edge to basipterygoid tip is almost identical to *Tsaagan*'s when scaled to orbit+jugal height. *Ceratonykus* does have a very long basipterygoid process (Alifanov and Barsbold, 2009: Fig. 1f) exposed in posteroventral view. Given the lack of published verification for particularly long processes in *Haplocheirus* and *Shuvuuia* and the difficulty in measuring partially exposed obliquely oriented structures, this potential alvarezsauroid character is rejected pending further data.

V-shaped opening between basal tubera remnants 0: absent 1: present (454 in Turner et al., 2012). Turner et al.'s character is coded as present in IGM 100/1128, but is merely a combination of notch shape and tuber size, both of which are already coded by other characters (239 and 413).

Small tubera (not basal tubera) medial to basal tubera (or basal tubera remnants) and ventral to occipital condyle 0: absent 1: present (455 in Turner et al., 2012). This is coded as present in IGM 100/1128, *Sinovenator*, *Byronosaurus* and *Chauna*, but none of these taxa seem to exhibit the condition (pers. obs.; Xu, 2002; Makovicky et al., 2003; DigiMorph Staff, 2001).

Prootic recess ORDERED 0: absent 1: present and shallow 2: present and deep (450 in Turner et al., 2012). Turner et al. only code this as present in ornithomimids and eudromaeosaurs, but prootic recess is just another term for the main portion of the anterior tympanic recess, which most theropods have. Oddly, Turner et al. also include a character coding for the anterior tympanic recess, which they give a different distribution for.

Ectopterygoid 0: present 1: absent (275 in Turner et al., 2012). Elzanowski (1999) provided good evidence the avian uncinate/lacrimopalatine is homologous with the ectopterygoid.

Symphyseal region of dentary rostrocaudally narrow, no broader than transverse width

of post-symphyseal portion (0) or broader than transverse width of post-symphyseal

portion (1) (260 in Zanno et al., 2009; 782 in Brusatte et al., 2014). This was coded in oviraptorosaurs, but only appears true due to the anterior angling of the symphysis, which in section is just as narrow as the rest of the dentary (e.g. IGM 100/42 Fig. 8.2 in Osmolska et al., 2004).

Dentary, morphology of dorsal border in transverse cross section: 0: rounded and lacks “cutting edge”. 1: sharp with a “cutting edge” (733 in Brusatte et al., 2014). This sharp edge is due to being toothless, which is already a character (290). Oddly, Brusatte et al. only scored toothless ornithomimosaurs as state 1 even though the same is true for toothless birds and oviraptorosaurs (e.g. Lu et al., 2004: Fig. 4).

Ventral surface of dentary straight or nearly straight (0) or descends strongly posteriorly (1) (224 in Kirkland et al. 2005). The authors code this as only present in therizinosaurs, but the feature (which is absent in *Falcarius* and *Segnosaurus* among their included taxa) is an illusion caused by two variables which are already coded for. First, the strongly triangular dentary (character 73) causes the posteroventral margin to be highly angled to the dorsal margin. Second, the decurved ramus (character 74) means the mandible is usually illustrated with the posterior dentary facing more ventrally, since the convention is to have the glenoid and dentary tip placed at approximately the same level. The character is thus rejected.

Anterior external mandibular fenestra 0: absent 1: present (310 in Turner et al., 2012). This was only coded as present in *Confuciusornis*, but it is evident from Clarke's (2002) discussion that it is based on a misunderstanding of that taxon's anatomy. While Clarke claimed the anterior fenestra of *Confuciusornis sanctus* was a neomorph and not homologous to the posterior fenestra of some neognaths and dromaeosaurids, *Confuciusornis dui* shows that *C. sanctus* merely has a surangular process dividing its ancestral mandibular fenestra. A surangular prong is already coded for here (character 78), so this character is ignored.

Surangular, anteroventral extension divides external mandibular fenestra by contacting angular anteriorly 0: absent 1: present Currie et al., 2003: char. 72 (257 in Turner et al., 2012). This was only coded present in *Allosaurus*, but is based on the incorrect composite of Madsen (1976). Articulated material such as DINO 11541 (Chure, 2000) or MOR 693 show a typical theropod mandible where the external mandibular fenestra contacts the dentary.

Longitudinal groove on labial surface of maxillary and dentary tooth crowns (venom groove of Gong et al. 2010) absent (0) or present (1) (143 in Senter, 2011). While only coded in *Sinornithosaurus*, *Graciliraptor* and *Cryptovolans* by Senter, Gianechini et al. (2010) showed these grooves are widespread in theropods. Indeed, they are probably present in some teeth of most species (e.g. Currie et al. [1990] illustrate *Dromaeosaurus*, *Saurornitholestes*, *Troodon* and *Richardoestesia* as being polymorphic), so are not coded for here.

Neck, length compared to that of skull: 0: less than twice skull length. 1: greater than twice skull length (735 in Brusatte et al., 2014). This is correlated with skull length, cervical number and cervical elongation, each of which is separately scored for here (characters 337, 100 and 356 respectively).

Orientation of articular surfaces between cervical vertebrae 0: surfaces vertical to subvertical 1: strongly slanted anteroventrally (465 in Turner et al., 2012). This was scored by Turner et al. as present in *Ornitholestes* and non-pygostylian pennaraptorans. However, most taxa exhibit a range of angles, with anterior centra generally having more strongly slanted surfaces than posterior centra. If a position could be specified (as in Maryanska et al.'s [2002] version which uses "anterior postaxial cervicals") and angle quantified, this character may be informative. Until then it is rejected.

Cervical prezygapophyses unflexed (0) or flexed (1) (263 in Senter, 2007; 264 in Turner et al., 2012). Originally coded as present in *Ornitholestes*, *Garudimimus* plus ornithomimids, and most maniraptorans excepting oviraptorosaurs. However, every sampled complete cervical column has some vertebrae which possess flexed prezygapophyses. No consistent positional variation was noted, though there may be useful variation if the degree of curvature is measured. Pending analysis of the latter, the character is rejected here.

Scars for interspinous ligaments terminate at apex of neural spine in dorsal vertebrae (0) or terminate below apex of neural spine (1) (110 in Norell et al., 2001). Coded as present in paravians and *Conchoraptor*, even the outgroups of that analysis (*Sinraptor* and *Allosaurus*) have some dorsals where the ligament scars only extend up ~80-85% of the neural arch. Additionally, the extent can vary between anterior and posterior edges of the neural spine. Thus the character is excluded here pending further study of its variation.

Caudal vertebrae with distinct transition point, from shorter centra with long transverse processes proximally to longer centra with small or no transverse processes distally (0), or vertebrae homogeneous in shape, without transition point (1) (116 in Norell et al., 2001; 191 in Senter, 2011). This character is problematic, as every theropod without a shortened tail has distal caudals which are more elongate than the proximal caudals and have smaller or absent transverse processes, even oviraptorids. While it may be possible to form a character which quantifies how abrupt the change in each feature is, this has not been attempted here. Senter reworded this character and separated taxa with distinct transition points into two states (his type 1 and type 2 transition points), but the differences between them (central elongation, prezygapophyseal elongation, and neural spine reduction) are all covered by separate characters here (characters 248, 127 and 126).

Cervical ribs, shaft: ... 2: extremely thin and slender, hair-like (122 in Brusatte et al., 2014). This was scored only for "compsognathid-grade" taxa, but is also present in other small taxa with good preservation and long cervical ribs such as *Hesperornithoides* (this paper), *Anchiornis* (Pei et al., 2017: Fig. 12) and *Archaeopteryx* (Wellnhofer, 1974: Fig. 10B). It is thus a function of size and preservation.

Wide distal expansion of scapula absent (0) or present such that scapular blade is an elongate triangle (1) or present such that scapular blade is hatchet-shaped (sudden widening about midway up the anterior edge, but widened only distally along posterior edge) (2) or present such that scapular blade is distally paddle-shaped, with rounded rather than pointed corners at the distal expansion (3) (212 in Senter, 2011). While scapular distal expansion is covered here by character 364, Senter's state 2 is an autapomorphy of *Dilophosaurus* in his matrix so is ignored here for now, and state 3 is only scored in *Anserimimus* and *Dromiceiomimus* (his *Ornithomimus*). Yet the distal scapula of *Anserimimus* is broken so that its corners are unknown (Barsbold, 1988: fig. 1b), and that of *Dromiceiomimus* (e.g. Parks, 1928b: plate 1) is no more rounded or paddle-shaped than e.g. *Caudipteryx* (Zhou et al., 2000: fig. 4) or *Haplocheirus* (Choiniere, 2010: fig. 6.19B). Pending further data, roundness (which independently varies for each corner so would be two separate characters, and would be difficult to quantify) is not scored here.

Scapula, a supra-glenoid-fossa-crest absent (0) or present (1) (241 in Xu et al., 2009). This is only coded as present in avialans, but no feature corresponding to it can be located on their scapulae.

Coracoids with smooth external surface (0), or ventral part of lateral face bears rugose vermiform sculpting (1) (302 in Makovicky et al., 2012). This was coded as proposed by Agnolin et al. (2012) as a synapomorphy uniting *Patagonykus* and *Bonapartenykus*, but as the latter authors admit *Patagonykus* "shows only isolated and poorly developed anastomosed grooves." Indeed, the figure (10B in Novas, 1997) only shows two widely separated transverse grooves, not obviously homologous to the rugose sculpting in *Bonapartenykus*. The sculpting is thus considered an autapomorphy here and not included in the analysis.

Arm short (0) or very short (1) or long (2) or significantly elongated (3) relative to femur (31 in Xu and Zhang, 2005). This is a correlated with humeral length, which is coded by another character (254).

Humerus, robustness relative to tibiotarsus significantly more slender than (0) or sub-equal or more robust than (1) tibiotarsus (250 in Xu et al., 2009; 362 in Xu et al., 2011a). Humerofemoral and tibiofemoral ratios and humeral robustness are already coded by other characters (254, 383 and 366).

Humerus, proximal end, proximal projection 0: dorsal edge projected farthest 1: midline projected farthest (358 in Turner et al., 2012). This is caused by the convex humeral head, which is coded by another character (610).

Humerus - Crest-like internal tuberosity on humerus absent (0) or present (1) (32 in Xu, 2002; 285 in Zanno et al., 2009). Quantifying this character is difficult due to the gradual distal emergence of the process from the shaft. Until its proximodistal length is better defined, the character is provisionally rejected.

Distal humeral condyles on distal end (0) or on anterior surface (1) (226 in Kirkland et al., 2005; 371 in Turner et al., 2012). While this is a standard character distinguishing alvarezsaurs, therizinosaurs and birds, the reality is more complex. Most theropods have both anterior and ventral exposure of entocondyle and ectocondyle, of which the anterior exposure is often exaggerated due to anterior flexure of the humeral distal end. As an example of the basal condition, in *Ornitholestes* (YPM 56681, cast of holotype), the entocondyle has extensive anterior exposure but no defined posterior exposure while the ectocondyle has about 40% of its surface exposed posteriorly, albeit less well defined than the anterior exposure. Another traditionally plesiomorphic taxon, *Velociraptor* (IGM 100/986) has 40% of its entocondyle exposed posteriorly and at least 30% of its ectocondyle exposed posteriorly. Notably, the anterior exposure is proximodistally extensive and rounded in shape so that if posterior views were unavailable, one might assume it to have condyles restricted to the anterior surface. In Alvarezsauridae, while neither *Patagonykus* or *Mononykus* have functional ectocondyles, the former has an entocondyle which has about a third of its surface exposed posteriorly (Novas, 1997), and the latter has about a fourth exposed posteriorly (YPM 56693, cast of holotype). In Therizinosauria, about a third of *Falcarius'* entocondyle and ectocondyles are exposed posteriorly (Zanno, 2006). Birds such as *Archaeopteryx* (Wellnhofer and Roper, 2005) and *Sapeornis* (Pu et al., 2013) have about a third of their ectocondyles visible posteriorly. Given the variation noted above between ecto- and entocondyles and impossibility of coding from purely anterior views, this character is not used here pending further study.

Antebrachial bones stout (radial shaft length < 8x diameter, ulnar shaft length < 6x diameter) (0) or gracile (radial shaft length > 8x diameter, ulnar shaft length > 6x diameter (1 ) (233 in Senter, 2011). This was coded as present in tyrannosaurids (incorrect), *Compsognathus*, *Ornitholestes* and most non-alvarezsauroid maniraptoriforms. It is correlated with humeral gracility and the radiohumeral ratio, both of which are already represented in the dataset (characters 366 and 540).

Ulnar/femoral length ratio: significantly less than one (0) or equal or greater than one (1) (236 in Makovicky et al., 2005; 277 in Senter, 2007). This is a correlated with humeral length and radiohumeral ratio, which are coded by other characters (254 and 540).

Ulna, robustness relative to tibiotarsus significantly more slender than (0) or more robust than (1) tibiotarsus (253 in Xu et al., 2009). This is a correlated with humeral length, which is coded by another character (254).

Ulna, proximal end, articular surface for ulna condyle flat mediolaterally and longer anteroposteriorly than transversely (0) or a bowl-like fossa, subequal in anteroposterior and mediolateral width (1) (255 in Xu et al., 2009). While either cotyla shape or convexity may include useful characters, they are difficult to code in most taxa due to the rarity of anteriorly exposed ulnae and poorly defined cotylar edges.

Radius and ulna well separated (0) or with distinct adherence or syndesmosis distally(1) (211 in Hwang et al., 2004). This character involves the distal syndesmosis or adherence between the radius and ulna in ornithomimosaurs, as first proposed by Nicholls and Russell (1985) in *Struthiomimus*. In that taxon, the proximal articulation is deeply concave on the ulna and the distal articulations are flattened, which the authors interpreted as indicating syndesmosis, where a ligament joins bones in barely mobile articulation. Yet these articulations in Aves match morphologically, but are not joined by ligaments. Rather, they are capable of sliding motion which enables the wing to fold. Thus the articular surfaces themselves are not evidence of reduced mobility in *Struthiomimus*. There are two forms of proposed motion between the radius and ulna of theropods. Rotation of the radius causing pronation has been proposed in *Microvenator* (Makovicky and Sues, 1998; based on the concave distal ulnar facet), *Troodon* (Russell, 1969; based on the roughly circular proximal radius) and *Deinonychus* (Ostrom, 1969; based on the slightly concave proximal ulnar facet and supposedly poorly developed articular surfaces). Yet *Microvenator* and *Deinonychus* both have triangular proximal radii that did not allow rotation (Gishlick, 2002; Senter, 2006b), though the morphology of *Troodon* requires further study. Thus far, all studied saurischians lack the ability to rotate their radius. The other proposed form of motion is that present in birds where the radius slides distally during flexion of the elbow. Carpenter (2002) argued this was absent in non-avian theropods due to the lack of a well developed incisura radialis for the radius to slide in proximally, which was also the osteological correlate found by Vasquez (1993). Indeed this seems true for some taxa such as *Allosaurus* (Carpenter, 2002), *Tyrannosaurus* (Carpenter and Smith, 2001), *Ornitholestes* (Senter, 2006a) and *Deinonychus* (Senter, 2006b; though movement was proposed by Gishlick [2002], this seems to be slight). Yet some maniraptorans such as *Oviraptor* (pers. obs.), *Sinornithoides* (Currie and Dong, 2001), *Microraptor* (Xu et al., 2000), *Archaeopteryx* (Vasquez, 1993) and *Confuciusornis* (Chiappe et al., 1999) possess the sliding morphology based on articulated specimens, and this is also true in *Bambiraptor* based on manipulation (Senter, 2006b). At least the latter taxon also has a well developed incisura radialis. *Segnosaurus* (unpublished photo of IGM 100/83) and *Khaan* (Gishlick, 2002) have a reversed articulation with a concave radius, suggesting another mechanism to obtain the same function. Yet some non-maniraptorans like *Coelurus* (Gishlick, 2002), *Tanycolagreus* (Carpenter et al., 2005a) and ornithomimosaurs also have a deep incisura radialis, but lack most features of the avian wing-folding mechanism, suggesting the incisura depth may not always indicate a sliding radius. Alvarezsaurids also deserve mention, as the two proximal facets in *Patagonykus* (Novas, 1997) and sutured connection in parvicursorines (Perle et al., 1994) make them the only theropods which definitely lack any motion between the radius and ulna. Despite frequent claims of syndesmosis in ornithomimosaurs (e.g. Perez-Moreno et al., 1994; Kobayashi, 2004; Makovicky et al. 2004), no feature of their morphology suggesting a stronger radioulnar articulation than more basal coelurosaurs has been described. The character is thus rejected.

Ulnare 0: absent 1: present (387 in Turner et al., 2012). This is based on Norell and Clarke's (2001) character 136, which incorrectly assumed ulnares are a neomorph developed in birds. In reality, ulnares are present in numerous more basal theropods, making cases of their absence probably due to preservation. Turner et al. score two taxa as lacking ulnares. Of these, *Archaeopteryx* preserves ulnares in several specimens including the Eichstatt example (Wellnhofer, 1974), while it was misidentified in *Struthiomimus* by Nicholls and Russell (1985) as a pisiform.

Lateral proximal carpal (ulnare?) quadrangular (0) or triangular in proximal view (1) (146 in Norell et al., 2001). This was coded initially as quadrangular in *Allosaurus*, *Gorgosaurus*, *Harpymimus*, *Gallimimus* and *Struthiomimus* and triangular in *Alxasaurus*, IGM 100/42, *Velociraptor*, *Deinonychus*, *Archaeopteryx* and *Confuciusornis*, thus supporting Maniraptora. Yet of these taxa, *Gallimimus*, *Velociraptor* and *Deinonychus* do not preserve the element, and it is missing from the mount of IGM 100/42 and has never been described in that specimen. Of the remaining, *Harpymimus* and *Struthiomimus* actually have more triangular ulnares, those of *Archaeopteryx* and (published) *Confuciusornis* are visible in extensor view, and that of *Alxasaurus* has only a "conjectural" orientation according to its authors. Since so few ulnares are preserved and visible in proximal/distal view in Mesozoic theropods, and the shape difference is poorly defined (shortening multiple edges could make the element more triangular), the character is here rejected pending further study.

Semilunate distal carpal well developed, covering all of proximal ends of metacarpals I and II (0) or small, covers about half of base of metacarpals I and II (1) or covers bases of all metacarpals (2) or covers metacarpals II and III (3) (148 in Turner et al., 2007b; 266 in Xu et al., 2009; 145 in Cau et al., 2015). While the portion of this character involving semilunate size is coded for here (character 157), the portion involving which metacarpals are covered is more problematic. Turner et al. code their two ornithothoracine examples as lacking semilunate coverage on metacarpal I, but the medial edges of the semilunate and metacarpal II are so close in numerous cases so that objective scoring is difficult. The same is true for metacarpal III, which often underlies metacarpal II to some extent on their proximal surfaces. Perhaps a character coding for which percentage of each metacarpal is covered would be more objectively scorable.

Combined lengths of metacarpal I and phalanx I-1 no more than 1.5 times longer (0) or more than 1.9 times longer (1) or shorter (2) than the length of metacarpal II (34 in Xu and Zhang, 2005; 285 in Senter, 2007; 444 in Turner et al., 2012). Metacarpal I and phalanx I-1 length are already compared to metacarpal II in other characters (158 and 257).

With fingers extended, tip of ungual I does not extend past flexor tubercle of ungual II (0) or extends past flexor tubercle of ungual II but does not extend past tip of ungual II (1) or extends past tip of ungual II (2) (345 in Senter, 2007). Metacarpal I, phalanx I-1 and digit II length are already compared to metacarpal II in other characters (158, 257 and 376). Note Senter (2011; his character 258) states taxa are coded as inapplicable if their manual ungual I is "hugely enlarged", which underscores the character's composite nature.

Length of manual digit II (including metacarpal) less than 1.25 × femoral length (0) or ? 1.25 × femoral length (1) (280 in Senter, 2007; 267 in Xu et al., 2009). This is a correlated with humeral length, which is coded by another character (254).

Manual phalanx II-1 shorter than I-1 (0) or longer (1) (328 in Senter, 2007; 298 in Zanno et al., 2009). These phalanges are already measured compared to II-2 and metacarpal II respectively (374 and 257).

Manual phalanx II-1, length compared to that of metacarpal 1: 0: longer. 1: subequal to. (693 in Brusatte et al., 2014). These bones are already measured compared to II-2 and metacarpal II respectively (374 and 158).

Length of manual phalanx II-1 <2 × length of III-1 (0) or ?2 × length of III-1 (1) (292 in Senter, 2007). These phalanges are already measured compared to II-2 and III-2 respectively (374 and 258).

Intermetacarpal space (between metacarpals II and III) 0: reaches proximally as far as the distal end of metacarpal I 1: terminates distal to end of metacarpal I (396 in Turner et al., 2012). Turner et al. based their character on 147 of Norell and Clarke (2001), but these are dependent on metacarpal I length, which is already coded for by character 158. Norell and Clarke and Turner et al. only coded some Aves as derived (and Turner et al. additionally coded most taxa as inapplicable for no evident reason), so no information is lost here, although this could be made into an intermediate state for character 372 in future analyses.

Metacarpal III, length: 0: considerably longer than length of metacarpal I. 1: approximately same length as, or slightly longer than, metacarpal I (767 in Brusatte et al., 2014). This is a composite of metacarpal I and III lengths (characters 158 and 371 respectively).

Medial ligament pits of manual phalanges deep (0) or shallow (1) (289 in Senter, 2007; 302 in Zanno et al., 2009; 800 in Brusatte et al., 2014). Used by Senter to help diagnose therizinosauroids more derived than *Beipiaosaurus*, it is also true in some phalanges of some ornithomimids, caenagnathids, microraptorians and numerous ornithothoracines. Until the character is defined more precisely for particular phalanges, it is excluded from the analysis.

Diameter of non-ungual phalanges of manual digit III >0.5× diameter of non-ungual phalanges of digit II (0) or < 0.5× (1) (327 in Senter, 2007). Originally coded as present in tyrannosauroids, most compsognathids, *Falcarius*, caenagnathids and ornithurines sensu Gauthier. This may be developed into at least two characters, as many taxa have II-1 more robust than II-2 while digit III remains a near constant width. It is also near certainly correlated with the diameters of metacarpals III and II, covered by character 635.

With fingers extended, tip of ungual III extends no further distally than flexor tubercle of ungual II (0) or extends further (1) (325 in Senter, 2007). This involves the lengths of all the component phalanges and metacarpals, many of which are coded separately here.

Length of manual unguals distal to flexor tubercle is much greater than height of articular facet (0) or is not (1) (363 in Senter, 2010). That this type of abbreviated ungual is only known in young juvenile specimens (*Scipionyx*, *Juravenator*, *Sciurumimus*) suggests it is not useful for determining the phylogeny of adult specimens. It may prove useful in diagnosing a grade of juvenile taxa, as it is lacking from e.g. *Yulong* and *Sinornithomimus*.

Ilium, supraacetabular crest, maximum lateral projection relative to ischial peduncle: 0: significantly greater. 1: subequal (696 in Brusatte et al., 2014). This is a combination of supracetabular crest projection (172) and antitrochanter projection (177).

Pubis, ischial peduncle distinct, inset from the proximal end, groove present between the shaft and the peduncle (0) or short, flush with the lateral surface of the pubic shaft (1) (271 in Xu et al., 2009). Xu et al.'s codings do not divide taxa by ischial peduncle length (e.g. *Shuvuuia* and *yanshini* have extremely short peduncles but are not coded this way), and any difference in lateral projection, if present, is too subtle to be noticed in photographs or illustrations.

Pubis, shaft close to the proximal end, anteroposterior width less than 1.5 times of the mediolateral width (0) or more than 2 times of the mediolateral width (1) (272 in Xu et al., 2009). This character was left uncoded for almost all taxa by Xu et al., but was coded as present in *Microvenator*, *Rahonavis* and *Apsaravis*. One issue is that pubic width varies greatly proximal to the symphysis, often by a factor of two. *Microvenator* actually has a ratio of 1.43 for the narrowest part of its proximal pubis (pers. obs. AMNH 3041), while *Rahonavis* has a ratio of 1.64 (cast YPM 56587). *Apsaravis* has approximately equal values based on figure 10 of Clarke and Norell (2002). Until the character is defined more objectively and proposed for taxa which actually possess it, it is rejected.

Pubis, shaft close to the distal end, anteroposterior width thin anteroposteriorly, with a ridged lateral margin (0) relative thick, without a ridge (1) (273 in Xu et al., 2009). This is only coded as present in *Rahonavis* and *Apsaravis*, yet the former has a ridged margin like other paravians and the latter's pubis is only exposed medially.

Hyperenlargement of pubic boot to > 2/3 length of pubis: absent (0) or present (1) (312 in Senter, 2011; 704 in Brusatte et al., 2014). This was coded by Senter as present in tyrannosaurines and by Brusatte et al. as present in tyrannosaurids and some therizinosauroids, but is a consequence of enlargement of both anterior and posterior boots, here coded separately.

Semicircular scar on posterior part of the proximal end of the ischium, absent (0) or present (1) (171 in Norell et al., 2001). The proximolateral ischial scar coded for in this character has been shown by Hutchinson (2001a) to be present in most theropods when well preserved and homologous with the proximodorsal process of character 180.

Ischium, shaft minimum anteroposterior width less than (0) or more than 20% (1) of the ischial length (277 in Xu et al., 2009). Ischial length and depth compared to the pubis are already coded by other characters (187 and 382).

Ischium with rodlike shaft [i.e. part distal to acetabular portion] (0) or with wide, flat, and plate-like shaft (1) (166 in Makovicky et al., 2005; 329 in Senter, 2007). This is coded as present in therizinosaurs, oviraptorosaurs and paravians. Yet the ischial shaft proximal to the obturator process is similarly compressed in *Allosaurus* (41% of depth- Gilmore, 1920) and *Saurornithoides* (47%- AMNH 6516). Assuming a similar transverse width in theropod ischia, those of maniraptorans are deeper for more of their length primarily due to their distally placed obturator processes. Not only is there more deep area proximal to the obturator process, but the generally similar amount of distal taper leaves less of the ischium to be rod-like. Indeed, the only taxa coded by Makovicky et al. as having plate-like ischia which also have proximally placed obturator processes are *Archaeornithomimus* (described by Smith and Galton [1990] as having a rounded distal shaft, so probably miscoded) and *Achillobator* (which is described as compressed by Perle et al. [1999], and does retain its depth distally). Until an exact area of the ischium is specified as being more compressed in maniraptorans, and this is found to be independent of obturator placement, the character is rejected.

Ischium ... or twisted at midshaft and with flexure of obturator process toward midline so that distal end is horizontal (2) (167 in Norell et al., 2001). This was included as a state in a composite character otherwise scoring for shaft curvature in side and anterior view, scored only for *Velociraptor* and *Deinonychus*. While at least *Velociraptor* does exhibit a medially flexed obturator process, its degree is exaggerated by the lateral ridge (character 182), and medial flexion is present in most theropods (e.g. *Allosaurus*- Gilmore, 1920: Plate 12 Fig. 1-2; *Dromiceiomimus*- Hutchinson, 2001a: Fig. 10; *Saurornithoides*- pers. obs. AMNH 6516) with the exception of at least some therizinosauroids (*Enigmosaurus*- Zanno, 2010b: Fig. 3A, C; *Suzhousaurus*- Li et al., 2008: Fig. 7C-D) where the process flares laterally to contact the pubis (character 184). Regarding the distal ischial end, obturator process orientation would only affect it in taxa with distally placed processes (character 183) and indeed known cases have oblique distal ends like *Velociraptor* (e.g. *Saurornithoides*; *Bambiraptor*- Burnham, 2004: 3.28C-D). Whether *Velociraptor's* obturator process and/or distal ischial tip are more horizontally angled than other taxa is difficult to quantify and given the absence of an obvious extreme condition in other taxa (e.g. *Deinonychus*- Ostrom, 1969: Fig. 65A), the character is here rejected.

Femur, femoral head, posterior deflection: absent (0); present, femoral head more posteriorly positioned than greater trochanter (1) (856 in Yu et al., 2018). While stated in the text to be present in *Anomalipes* and *Gigantoraptor*, the former is correctly scored as lacking the condition in their matrix, and no oviraptorosaur is scored as possessing it. Pending its discovery in other taxa, this seems to be an autaponmorphy of *Gigantoraptor* and so is not used here.

Femur, distal end, medial condyle transverse width: sub-equal or greater (0); significantly less (1) than the lateral condyle transverse width (281 in Xu et al., 2009). This character needs more precise definition, which may not be scorable unless in distal view, as the condyles often grade into the intercondylar sulcus.

Femur, distal end, longitudinal ridge extending proximally from the medial condyle on the posterior margin: present, forming a prominent posterior intercondylar groove (0) or absent, without a distinct groove (1) (280 in Xu et al., 2009). This was only coded as state 1 in *Rahonavis*, making it phylogenetically uninformative in Xu et al.'s analysis. However, *Rahonavis* is similar to most other theropods in having this ridge (cast YPM 56587), and some taxa coded as 0 by the authors (e.g. *Velociraptor*, *Unenlagia*) actually have less distinct ridges. This and the fact no easily codable difference exists between the prominence or sharpness of the medial popliteal ridge in Mesozoic theropods results in the character being rejected pending further study.

Fibular crest of tibia with smooth margins (0) or hooked proximally (1) (361 in DePalma et al., 2015). This was stated to be an autapomorphy of *Dakotaraptor*, so until it is verified in another OTU, adds no information to the analysis.

Metatarsal III, distal articulation, medial hemicondyle, transverse width: sub-equal to transverse width of lateral hemicondyle (0); much less than transverse width of lateral hemicondyle (1) (864 in Yu et al., 2018). This was stated to be an autapomorphy of *Anomalipes* and was not scored as present in any other taxa in either the authors' TWiG or oviraptorosaur matrices, so is here excluded pending identification in other taxa.

Pedal phalanx II-1 longer (0) or shorter (1) than pedal phalanx IV-1 (342 in Senter, 2007). This is correlated with the character comparing lengths of II-1 and II-2 (267).

Pedal phalanx II-2 with distinct shaft or waist between proximal cotyles and distal condylar eminence (0) or shaft eliminated by extreme shortening of this phalanx (1) (381 in Senter, 2011). This was coded as present in *Borogovia* and *Troodon*, but only the first exhibits the condition. In *Troodon* (e.g. CMN 1650) there is actually more of a waist than in *Saurornithoides*. As an apparent autapomorphy of *Borogovia*, this is provisionally rejected, though the short phalanges of *Talos* and *Linhevenator* may warrant a similar character.

Ungual and penultimate phalanx of pedal digit II similar to those of III (0) or penultimate phalanx highly modified for extreme hyperextension, ungual more strongly curved and about 50% larger than that of III (1); penultimate phalanx of digit II modified for hyperextension but ungual not hypertrophied (2) (170 in Mayr et al., 2005). Pedal phalanx II-1 without dorsal extension of distal condyles (0) or with extension (1) (323 in Senter, 2007). Mayr et al. added a state to Norell et al.'s (2001) sickle claw character to code for the second pedal digit of *Archaeopteryx*, which is hyperextendable but lacks an enlarged ungual. Similarly, Senter codes *Archaeopteryx* and later (2010) *Anchiornis* and caenagnathids as having that state. Yet Senter (2009) finds that even taxa with no specialization for hyperextension such as *Allosaurus* and *Mononykus* are capable of hyperextending their second pedal digit within the range of deinonychosaurs. The dorsal condyles of phalanx II-2 are dorsally extended in most theropods, though perhaps a study of the angle or abruptness of the extension would show codable differences.

Pedal unguals robust, straight (0) or recurved, with thick ventral margin (1) or parallel-sided, slender, and strongly recurved (2) (51 in Xu, 2002). While most of these variables are covered in this analysis (characters 273 and 274), robusticity was not. Presumably quantified as dorsoventral depth at some point compared to proximodistal length for each ungual, this remains a potential character to incorporate.

**Taxa**

**Unnamed taxa**

Numerous OTUs were included that lack Linnaean nomenclature. MCF-PVPH-237 is a partial abelisaurid postcranium described by Coria et al. (2006). The MNNGAD postcranium (MNN GAD1 in part- three dorsals, two ribs, sacrum and pelves) was initially described as part of *Kryptops* (Sereno and Brusatte, 2008), but was suggested to be carcharodontosaurid by Carrano et al. (2012). The Zuni taxon is a coelurosaur mentioned by Denton et al. (2004). The Nanchao embryos are the therizinosaur specimens described by Kundrat et al. (2007). AMNH 6368 is a therizinosaur forelimb initially assigned to *Alectrosaurus* (Mader and Bradley, 1989). NGMC 2124 is a skeleton originally referred to *Sinosauropteryx* (Ji and Ji, 1997). The Angeac taxon is a supposed ornithomimosaur known from a bonebed (Allain et al., 2014). GIN960910KD is an ornithomimosaur sometimes referred to *Harpymimus* (Kobayashi and Barsbold, 2002). *Gallimimus*? "mongoliensis" is a nomen nudum based on IGM 100/14 (Kobayashi and Barsbold, 2006) and IGM 950818 (Kobayashi, 2004). MNA Pl.1762A is a partial skeleton referred to *Ornithomimus velox* by DeCourten and Russell (1985). The Cerro del Pueblo ornithomimid is a Mexican taxon described by Aguillon Martinez (2010). The 'Naze dromaeosaur' is a specimen from Antarctica (Case et al., 2007) more recently (Ely and Case, 2016) recovered as a basal deinonychosaur. The Tugrik parvicursorine is based on two specimens (IGM 100/99 and 100/120) originally referred to *Shuvuuia*, but separated by Longrich and Currie (2009). IVPP V9608 is a nesting oviraptorid described as *Oviraptor* by Dong and Currie (1996). NXMV is an oviraptorid partial skeleton from the Pingling Formation described by Lu (2004). IGM 100/42, the Zamyn Khondt oviraptorid, is a complete specimen initially referred to *Oviraptor* (Barsbold, 1981) but more recently compared to *Citipati*. IVPP V11119 was referred to *Sinornithoides* sp. by Dong (1997), while ISMD-VP09 is a partial skeleton from Oosh in Mongolia described by Prieto-Marquez et al. (2012) as a deinonychosaur. Originally called "Koreanosaurus", DGBU-78 is a Korean femur described by Kim et al. (2005) as a possible dromaeosaurid. IGM 100/1128 has been called the Zos Canyon troodontid and was recently recovered as a jinfengopterygine by Turner (2008 and derivatives) and described by Pei (2015). IGM 100/140 is a partial troodontid skeleton described by Tsuihiji et al. (2015), and IGM 100/44 (Barsbold et al., 1987) has long been included in TWiG analyses as the unnamed Early Cretaceous troodontid. IGM 100/792 and 100/974 are two perinate skulls first identified as *Velociraptor*, then described in depth as *Byronosaurus* (Bever and Norell, 2009). BYU 2023 is a proximal femur from the Morrison Formation described as *Archaeopteryx*-like by Jensen (1981). NGMC 91 is a young microraptorian (Ji et al., 2001) referred to *Sinornithosaurus* by Turner et al. (2012). IGM 100/22 and 100/23 are dromaeosaurid specimens from the Bayanshiree Formation initially referred to *Adasaurus*, but later found to be distinct (Kubota and Barsbold, 2007). IGM 100/980 is a dromaeosaurid postcranium (Norell and Makovicky, 1999) described in depth by Turner (2008). "Proornis" is a nomen nudum for a Korean confuciusornithiform most recently featured in Gao et al. (2009: Fig. 2A-B). PMO 228.582 is an Albian femur from Norway described as a possible avialan (Hurum et al., 2016). LP-4450-IEI is a partial juvenile enantiornithine notable for its rather complete skull (Sanz et al., 1997). CAGS-IG-02-0901 (You et al., 2005), CAGS-IG-04-CM-007 (Lamanna et al., 2006) and CAGS-IG-04-CM-023 (Harris et al., 2006) are three enantiornithine specimens from the Xiagou Formation of Gansu, China.

**Excluded taxa**

All named Mesozoic maniraptoromorphs described through 2018 and known from more than teeth or single elements were included with few exceptions. Testing indicated *Valdoraptor*, *Unquillosaurus*, *Canadaga* and *Gallornis* each had spurious positions due to their fragmentary remains and the current character sample, although the addition of new characters could change this in future iterations. *"Ornithomimus" minutus* is only known from a paragraph of text due to loss of the holotype and absence of illustration, so that possible hyperarctometatarsaly (219:2/3) is the only scorable character. *Gobiraptor* and *Shangyang* were published too late to be scored in the matrix. Finally, the chimaerical *Bagaraatan*, *Beipiaognathus* and *Dalianraptor* were not included pending detailed reanalysis of their types.

A large amount of isolated coelurosaur remains are known from the Bissekty Formation, which have generally been added to phylogenetic analyses by their describers after combining all elements into a single composite taxon - Sues and Averianov (2015b) for ornithomimid material; Sues and Averianov (2015c) for therizinosauroid material; Sues and Averianov (2015a) for caenagnathoid material; Sues and Averianov (2014) for dromaeosaurid material; Averianov and Sues (2016) for troodontid material. These could each easily be chimaerical since multiple species of each group are generally found in well sampled formations, and as such, none of these composite taxa are used here.

**Restricted material**

The following is excluded to specify controversial material excluded from the hypodigm when scoring OTUs. *Alectrosaurus* was scored based on the lectotype only. *Therizinosaurus* excludes the hindlimb IGM 100/45 since it cannot be compared to the holotype or referred specimens IGM 100/15-100/17. *Sinosauropteryx* excludes NGMC 2124, noted as morphologically distinct by Longrich (2002). *Ornithomimus* only includes *O. velox* material from the Denver Formation. *Ornithomimus? sedens*' OTU is based only on the holotype (Gilmore, 1920). *Caenagnathasia* is only scored based on the mandibular material, as the caenagnathoid postcrania described by Sues and Averianov (2015a) could belong to other taxa. Similarly, *Urbacodon* is only based on mandibular and dental material, excluding other troodontid elements described by Averianov and Sues (2016). *Itemirus* was only scored based on the holotype braincase, excluding the referred Bissekty dromaeosaurid material (Sues and Averianov, 2014). *Variraptor* includes only the holotype posterior dorsal and sacrum, plus ilium CM-645 that articulates with them and is thought to belong to the same individual. *Hesperonychus* was only scored based on the holotype pelvis, not the referred pedal elements. Among the Sinpetru taxa, *Elopteryx* is only scored based on proximal femora, while *Heptasteornis* and *Bradycneme* are scored based on distal tibiotarsi. *Vorona* was only scored based on the hindlimb material, excluding the forelimb elements tentatively referred by O'Connor and Forster (2010). The Lecho Formation enantiornithines were each scored only based on material comparable to their holotypes, so that each *Martinavis* species is only scored from humeri, *Enantiornis* from pectoral and forelimb material (but not isolated sternum PVL-4021-2), and *Elbretornis* from its holotype.

**Synonymized taxa**

In addition to near-universally accepted examples, the following synonymies and material referrals were used when scoring OTUs. *Aoniraptor* is a junior synonym of *Gualicho*. *Cristatusaurus* includes *Suchomimus*. *Spinosaurus* sensu lato uses all Baharija and Kem Kem spinosaurine material, including *Sigilmassasaurus*. *Alioramus* includes *Qianzhousaurus*. *Ornithomimus edmontonicus* is a junior synonym of *Dromiceiomimus brevitertius*. *Chirostenotes* sensu lato includes material ascribed to *Chirostenotes*, *Caenagnathus*, *Macrophalangia* and *Epichirostenotes*. These largely non-overlapping remains exhibit only minor morphological variation and have been sorted by prior authors mostly using stratigraphy and size. Similarly, *Troodon* sensu lato includes material ascribed to *Troodon*, *Stenonychosaurus*, *Latenivenatrix* and the Two Medicine Formation troodontid. In this case the material is also separated largely for stratigraphic reasons, with most *Stenonychosaurus* elements briefly described over a century ago and adult Two Medicine remains so far lacking any comprehensive description. *Microraptor* includes not only *M. zhaoianus,* *M. gui* and *M. hanqingi* specimens, but also *Cryptovolans*, NGMC 00-12-A and IVPP V13476. *Unenlagia* sensu lato includes both species of *Unenlagia* plus *Neuquenraptor*, as is standard in TWiG analyses. *Archaeopteryx* includes all Solnhofen paravians except the recently diagnosed *Ostromia* based on the Haarlem/Teyler specimen. *Jeholornis* includes *J. palmapenis* and *Shenzhouraptor*, while *Jixiangornis* includes *Jeholornis curvipes*, so that the former is Jiufotang and the latter Yixian. *Sapeornis* includes all proposed omnivoropterygid taxa. *Pengornis* includes *Parapengornis*, *Chiappeavis* and IVPP V18632. *Gansus zheni* is a considered synonymous with *Iteravis*.

In a related issue, to generate basically complete entries for lithornithids and galliforms without having to include apomorphies of those clades, *Calciavis* supplemented *Lithornis* with scores for some characters (26, 27, 50, 122 (1/2 > 1), 123, 124, 129, 134, 161 (1/2 > 1), 181 (0/1 > 1), 187, 189, 191, 192, 205, 222, 223, 224, 225, 247, 264, 266, 267, 268, 272, 273, 276, 277, 278, 306, 359, 360, 377, 378, 386, 390, 394, 409, 432, 463, 464, 473 (1/2 > 2), 478, 515, 520, 522, 539, 562, 585 (1/2 > 1), 586, 596, 662 (0/1 >1), 672, 682, 687, 688, 695, 697, 699), *Pseudocrypturus* for 255 and 457, and indeterminate Green River Formation lithornithids for others (1, 119, 220, 397, 449 and 595), while *Gallus* supplemented *Meleagris* with scores for characters 38, 98, 113, 123, 151, 206, 351, 357, 358, 448, 450, 452, 609, 659, 663, 672 and 676.

**Taxa coded conservatively for ontogeny-related characters (signified by state N in the NEXUS file) due to evidence of immaturity**

*Eustreptospondylus, Sciurumimus, Eotyrannus, Bistahieversor* (in part)*, Raptorex, Alioramus* (in part)*, Coelurus, Tanycolagreus, Aorun*, *Scipionyx*, *Juravenator*, *Huaxiagnathus*, *Sinosauropteryx*, *Archaeornithoides*, *Santanaraptor*, *Pelecanimimus*, GIN960910KD, *Haplocheirus*, *Alvarezsaurus*, *Jianchangosaurus*, *Beipiaosaurus*, Nanchao embryos, *Similicaudipteryx* (in part), *Caudipteryx*, *Microvenator*, *Beibeilong*, *Yulong*, *Banji*, *Jiangxisaurus*, *Scansoriopteryx*, *Epidexipteryx*, *Almas*, IGM 100/972 and 100/974, *Hulsanpes*, ISMD-VP09, NGMC 91, *Bambiraptor*, *Eosinopteryx*, *Archaeopteryx*, *Zhongornis*, *Eoconfuciusornis*, "Proornis", *Protopteryx*, *Paraprotopteryx*, *Eopengornis*, *Pengornis*, LP-4450-IEI, *Bohaiornis*, *Parabohaiornis*, *Longusunguis*, *Shenqiornis*, *Sulcavis*, *Yuanjiawaornis*, *Jibeinia*, *Hebeiornis*, *Holbotia*, *Parvavis*, *Monoenantiornis*, *Cratoavis*, *Boluochia*, *Longipteryx*, *Rapaxavis*, *Archaeorhynchus* (in part), *Jianchangornis*, *Tianyuornis*.

**Positions of maniraptoromorphs pruned a posteriori**

In order to have a more resolved tree in Maniraptoromorpha, several taxa were pruned a posteriori. Their positions are noted here.

Zuni coelurosaur- Coelurosaur excluded from Megaraptora, Coeluridae+Proceratosauridae and *Huaxiagnathus*+Maniraptoriformes.

IVPP V11119- Coelurosaur excluded from many clades including Compsognathidae, Ornithomimosauria, Alvarezsauroidea+Therizinosauria, Oviraptorosauria, Avialae, Archaeopterygidae, Unenlagiinae and Troodontidae+Dromaeosauridae.

*Aristosuchus*- Coelurosaur closer to Aves than Megaraptora, excluded from Maniraptoriformes.

Naze dromaeosaur- Maniraptoriform excluded from Therizinosauria, Alvarezsauroidea, Deinocheiridae, *Archaeornithomimus*+*Garudimimus*, *Harpymimus*+*Struthiomimus* and *Protarchaeopteryx*+Pennaraptora.

Cerro del Pueblo ornithomimid- Ornithomimosaur closer to *Ornithomimus* than *Shenzhousaurus*, excluded from *Arkansaurus*+*Garudimimus* and *Tototlmimus*+*Struthiomimus*.

*Bradycneme*-Maniraptoran excluded from *Ceratonykus*+*Mononykus*, Therizinosauria, Oviraptorosauria and Deinonychosauria.

*Martharaptor*- Therizinosaurian excluded from *Alxasaurus*+*Segnosaurus*.

*Nanshiungosaurus? bohlini*- Therizinosaurid in the *Erlikosaurus*+*Segnosaurus* clade.

*Caenagnathasia-* Caenagnathid in the Caenagnathinae+Elmisaurinae clade.

*Leptorhynchos gaddisi*- Elmisaurine.

*Wulatelong*- Oviraptorine in the *Huanansaurus*+*Conchoraptor* clade excluded from *Nomingia*+ other oviraptorids.

IVPP V9608- Oviraptorine in the *Conchoraptor*+*Citipati* clade excluded from *Khaan*+*Conchoraptor.*

*Pneumatoraptor*- Paravian either a dromaeosaurine sister to *Achillobator* or a palaeognath sister to *Palaeotis*+*Struthio*.

BYU 2023- Deinonychosaur in the Unenlagiidae+Dromaeosauridae clade excluded from Halszkaraptorinae, Sinovenatorinae, *Gobivenator*+ other troodontids and *Deinonychus*+*Dromaeosaurus*.

*Ornithodesmus-* Unenlagiine in the *Pyroraptor*+*Unenlagia* clade.

*Jinfengopteryx*- Troodontid excluded from *Jianianhualong*+IGM 100/140, *Liaoningvenator*+IGM 100/1128 and *Sinusonasus*+*Troodon*.

*Philovenator*- Troodontid in the *Liaoningvenator*+*Troodon* clade excluded from *Sinusonasus*+*Troodon*.

DGBU-78- Eudromaeosaur excluded from Dromaeosaurinae.

*Hulsanpes*- Dromaeosaurine.

*Atrociraptor*- Dromaeosaurine in the *Itemirus*+*Dromaeosaurus* clade.

*Hesperonychus*- Avialan in the *Balaur*+ clade excluded from *jianchangensis*+*Zhongjianornis* and Pygostylia.

*Confuciusornis? chuonzhous*- Avialan in the *Balaur*+ clade excluded from several enantiornithine clades and *Protopteryx*+Ornithuromorpha.

*Avisaurus*- Pygostylian excluded from *Mirarce*+*Yungavolucris*, Longipterygidae, *Feitianius*+*Iberomesornis*, many small enantiornithine subgroups and *Protopteryx*+Ornithuromorpha.

*Soroavisaurus*- Confuciusornithiform excluded from “Proornis”+*Eoconfuciusornis*.

*Wyleyia*- Pygostylian in the *Yandangornis*+ clade excluded from *Yuanjiawaornis*+*Enantiornis* and *Fortunguavis*+Ornithuromorpha.

*Huoshanornis*- Ornithothoracine excluded from many enantiornithine groups and *Paraprotopteryx*+Ornithuromorpha.

*Potamornis*- Ornithothoracine excluded from Carinatae and many small enantiornithine and ornithuromorph clades.

*“Cathayornis” aberransis*- Enantiornithine excluded from CAGS-IG-04-CM-023+Longipterygidae, *Feitianius*+*Iberomesornis* and several small subgroups.

*“Cathayornis” chabuensis*- Enantiornithine excluded from *Sinornis*+*Cathayornis*, Longipterygidae, *Alexornis*+*Iberomesornis* and several small subgroups.

*Martinavis cruzyensis*- Enantiornithine in the *Concornis+Enantiornis* clade excluded from *Enantiornis*+Longipterygidae, *Feitianius*+*Iberomesornis* and several small subgroups.

*Noguerornis-* Enantiornithine in the *Concornis*+*Enantiornis* clade excluded from many small subgroups.

*Enantiophoenix*- Enantiornithine in a polytomy with *Sinornis*+*Cathayornis*, *Gobipteryx*+*Jibeinia* and Longipterygidae.

CAGS-IG-04-CM-007- Longipterygid excluded from *Holbotia*+*Longirostravis*.

*Boluochia*- Longipterygid in the *Holbotia*+*Longirostravis* clade.

*Halimornis*- Longipterygid in the *Holbotia*+*Longirostravis* clade.

*Martinavis? vincei-* Enantiornithine in the *Pterygornis*+*Vorona* clade excluded from *Qiliania*+*Lectavis* and *Mirarce*+*Vorona*.

*Nanantius*- Enantiornithine in the *Piscivorenantiornis*+*Vorona* clade excluded from *Yungavolucris*+*Vorona*.

*Martinavis? minor*- Enantiornithine in the *Elbretornis*+*Gurilynia* clade excluded from GMV-2158+GMV-2159.

*Horezmavis*- Ornithuromorph in the Songlingornithidae+ clade excluded from *Fumicollis*+*baileyi, Iteravis+Gansus, Tianyuornis+Longicrusavis, Chaoyangia+*Songlingornithidae and Carinatae.

*Maaqwi*- Ornithuromorph in the Songlingornithidae+ clade excluded from *Mystiornis*+Hesperornithes, Palaeognathae, Neognathae and several small groups.

*Gargantuavis*- Hesperornithine excluded from Hesperornithoidea.

*Jiuquanornis*- Carinate in the *Eogranivora*+ clade excluded from *Iaceornis*+*Palintropus*, Palaeognathae, Vegaviidae and Galliformes.

*Tingmiatornis*- Neognath excluded from some clades.

*Teviornis*- Anseriform in the *Vegavis*+*Anas* clade.

**Phylogenetic taxonomy**

In this section, a simple reference (X, year) means the exact definition was used in that reference. A modified reference (modified from X, year) means we have redefined at least one specifier to its eponymous type species (as per Phylocode Article 11.7). When both are noted, the simple reference is the publication which refined the definition published by the modified reference. For example, Clark et al. (2004) defined Therizinosauroidea as "the least inclusive clade containing *Therizinosaurus* and *Beipiaosaurus*", which Zanno (2010b) refined to "the least inclusive clade containing *Beipiaosaurus inexpectus* and *Therizinosaurus cheloniformis*." The NEW definitions are largely modifications of existing definitions which account for the poorly supported relationships among basal maniraptoran clades and basal paravian clades, and to ensure Sinovenatorinae and Jinfengopteryginae are exclusive.

Avetheropoda - (*Allosaurus fragilis* + *Passer domesticus*) (Holtz et al., 2004; modified from Padian et al., 1999; modified from Currie and Padian, 1997a)

Tyrannosauroidea - (*Tyrannosaurus rex* <- *Passer domesticus*) (modified from Sereno, 1998)

Tyrannosauridae - (*Gorgosaurus libratus* + *Albertosaurus sarcophagus* + *Daspletosaurus torosus* + *Tarbosaurus bataar* + *Tyrannosaurus rex*) (Holtz, 2004)

Maniraptoromorpha - (*Vultur gryphus* <- *Tyrannosaurus rex*) (Cau, 2018)

Proceratosauridae - (*Proceratosaurus bradleyi* <- *Allosaurus fragilis, Tyrannosaurus rex, Coelurus fragilis, Compsognathus longipes, Ornithomimus velox, Deinonychus antirrhopus*) (modified from Rauhut et al., 2010)

Coeluridae - (*Coelurus fragilis* <- *Proceratosaurus bradleyi, Tyrannosaurus rex, Allosaurus fragilis, Compsognathus longipes, Ornithomimus edmontonicus, Deinonychus antirrhopus*) (Hendrickx et al., 2015)

Compsognathidae - (*Compsognathus longipes* <- *Passer domesticus*) (Holtz et al., 2004)

Maniraptoriformes - (*Ornithomimus velox* + *Passer domesticus*) (Maryanska et al., 2002; modified from Holtz, 1996)

Ornithomimosauria - (*Ornithomimus velox* <- *Tyrannosaurus rex, Shuvuuia deserti, Therizinosaurus cheloniformis, Oviraptor philoceratops, Troodon formosus, Passer domesticus*) **NEW**

Deinocheiridae - (*Deinocheirus mirificus* <- *Ornithomimus velox*) (Lee et al., 2014)

Ornithomimidae - (*Ornithomimus velox* <- *Garudimimus brevipes*) (modified from Kobayashi, 2008)

Maniraptora - (*Passer domesticus* <- *Ornithomimus velox*) (Maryanska et al., 2002; modified from Padian et al., 1997; modified from Gauthier, 1986)

Therizinosauria - (*Therizinosaurus cheloniformis* <- *Tyrannosaurus rex, Ornithomimus edmontonicus, Mononykus olecranus, Oviraptor philoceratops, Troodon formosus*) (Zanno, 2010b)

Therizinosauroidea - (*Beipiaosaurus inexpectus* + *Therizinosaurus cheloniformis*) (Zanno, 2010b; modified from Clark et al., 2004)

Therizinosauridae - (*Therizinosaurus cheloniformis* + *Segnosaurus galbinensis* + *Erlikosaurus andrewsi* + *Nanshiungosaurus brevispinus*) (modified from Zhang et al., 2001)

Alvarezsauroidea - (*Alvarezsaurus calvoi* <- *Ornithomimus velox*, *Therizinosaurus cheloniformis, Passer domesticus*) **NEW**

Alvarezsauridae - (*Alvarezsaurus calvoi* + *Mononykus olecranus*) (Hendrickx et al., 2015; modified from Choiniere et al., 2010a)

Parvicursorinae - (*Mononykus olecranus* + *Parvicursor remotus*) (modified from Choiniere et al., 2010a)

Pennaraptora - (*Oviraptor philoceratops* + *Deinonychus antirrhopus* + *Passer domesticus*) (Foth et al., 2014)

Oviraptorosauria - (*Oviraptor philoceratops* <- *Therizinosaurus cheloniformis*, *Passer domesticus*) (modified from Hu et al., 2009)

Caenagnathoidea - (*Caenagnathus collinsi* + *Oviraptor philoceratops*) (Maryanska et al., 2002; modified from Sereno, 1999)

Caenagnathidae - (*Caenagnathus collinsi* <- *Oviraptor philoceratops*) (Maryanska et al., 2002; modified from Sereno, 1998)

Caenagnathinae - (*Caenagnathus collinsi* <- *Elmisaurus rarus*) (Hendrickx et al., 2015)

Elmisaurinae - (*Elmisaurus rarus* <- *Caenagnathus collinsi*) (Hendrickx et al., 2015)

Oviraptoridae - (*Oviraptor philoceratops* <- *Caenagnathus collinsi*) (Maryanska et al., 2002; modified from Sereno, 1998)

Oviraptorinae - (*Oviraptor philoceratops* + *Citipati osmolskae*) (Osmolska et al., 2004)

Paraves - (*Passer domesticus* <- *Oviraptor philoceratops*) (Holtz and Osmolska, 2004; modified from Sereno, 1998)

Eumaniraptora - (*Deinonychus antirrhopus* + *Passer domesticus*) (Maryanska et al., 2002; modified from Padian et al., 1997)

Deinonychosauria - (*Deinonychus antirrhopus* <- *Passer domesticus*) (Holtz and Osmolska, 2004; modified from Currie and Padian, 1997b)

Archaeopterygidae - (*Archaeopteryx lithographica* <- *Unenlagia comahuensis, Dromaeosaurus albertensis*, *Troodon formosus, Passer domesticus*) **NEW**

Anchiornithinae- (*Anchiornis huxleyi* *<- Archaeopteryx lithographica*, *Unenlagia comahuensis*, *Epidexipteryx hui*, *Gallus gallus*, *Dromaeosaurus albertensis*, *Troodon formosus*) (modified from Xu et al., 2016)

Unenlagiidae- (*Unenlagia comahuensis <- Archaeopteryx lithographica, Troodon formosus, Dromaeosaurus albertensis, Passer domesticus*) **NEW**

Halszkaraptorinae - (*Halszkaraptor escuilliei* <- *Saurornithoides mongoliensis, Unenlagia comahuensis, Dromaeosaurus albertensis, Vultur gryphus*) (Cau et al., 2017)

Unenlagiinae - (*Unenlagia comahuensis <- Halszkaraptor escuilliei, Microraptor zhaoianus, Velociraptor mongoliensis, Dromaeosaurus albertensis, Passer domesticus*) **NEW**

Troodontidae - (*Troodon formosus* <- *Ornithomimus velox, Mononykus olecranus, Therizinosaurus cheloniformes, Oviraptor philoceratops, Archaeopteryx lithographica, Unenlagia comahuensis, Velociraptor mongoliensis, Passer domesticus*) **NEW**

Sinovenatorinae - (*Sinovenator changii* <- *Jinfengopteryx elegans*, *Troodon formosus*, *Passer domesticus*) **NEW**

Jinfengopteryginae - (*Jinfengopteryx elegans* <- *Sinovenator changii, Troodon formosus, Passer domesticus*) (Turner et al., 2012)

Troodontinae - (*Troodon formosus* + *Saurornithoides mongoliensis*) (Martyniuk, 2012)

Dromaeosauridae - (*Dromaeosaurus albertensis* <- *Unenlagia comahuensis, Troodon formosus*, *Archaeopteryx lithographica*, *Passer domesticus*) **NEW**

Microraptoria - (*Microraptor zhaoianus* <- *Velociraptor mongoliensis, Dromaeosaurus albertensis*) (modified from Senter et al., 2004)

Eudromaeosauria - (*Saurornitholestes langstoni* + *Velociraptor mongoliensis* + *Deinonychus antirrhopus* + *Dromaeosaurus albertensis*) (Longrich and Currie, 2009)

Velociraptorinae - (*Velociraptor mongoliensis* <- *Dromaeosaurus albertensis*) (modified from Sereno, 1998)

Dromaeosaurinae - (*Dromaeosaurus albertensis* <- *Velociraptor mongoliensis*) (modified from Sereno, 1998)

Avialae - (*Passer domesticus* <- *Deinonychus antirrhopus*) (modified from Padian, 2004; modified from Gauthier, 1986)

Scansoriopterygidae - (*Epidexipteryx hui* + *Epidendrosaurus ningchengensis*) (Godefroit et al., 2013; modified from Zhang et al., 2008)

Jeholornithidae - (*Jeholornis prima* + *Jeholornis palmapenis* + *Jeholornis curvipes*) (Lefevre et al., 2014)

Pygostylia - (*Confuciusornis sanctus* + *Passer domesticus*) (Turner et al., 2012; modified from Chiappe, 2001)

Confuciusornithiformes - (*Confuciusornis sanctus* <- *Enantiornis leali*, *Passer domesticus*) (Martyniuk, 2012)

Ornithothoraces - (*Iberomesornis romerali* + *Passer domesticus*) (Turner et al., 2012; modified from Chiappe, 1995b)

Enantiornithes - (*Enantiornis leali* <- *Passer domesticus*) (modified from Longrich, 2009)

Longipterygidae - (*Longipteryx chaoyangensis* + *Longirostravis hani*) (O'Connor et al., 2009)

Pengornithidae - (*Pengornis houi* + *Eopengornis martini*) (Wang et al., 2014)

Ornithuromorpha - (*Patagopteryx deferrariisi* + *Passer domesticus*) (modified from Chiappe, 2002)

Songlingornithidae - (*Songlingornis linghensis* <- *Chaoyangia beishanensis, Passer domesticus*) (Martyniuk, 2012)

Hesperornithes - (*Hesperornis regalis* <- *Passer domesticus*) (modified from Clarke, 2004)

Hesperornithoidea - (*Baptornis advenus* + *Hesperornis regalis*) (Martyniuk, 2012)

Carinatae - (*Passer domesticus* <- *Hesperornis regalis*) (modified from Cracraft, 1986)

Aves - (*Struthio camelus* + *Tinamus major* + *Vultur gryphus*) (Gauthier and de Queiroz, 2001; modified from Gauthier, 1986)

Palaeognathae - crown (*Struthio camelus* + *Tinamus major*) (Gauthier and de Queiroz, 2001)

Neognathae - crown (*Pluvialis apricaria* <- *Struthio camelus*, *Tinamus major*) (Gauthier and de Queiroz, 2001)

**Unpublished resources used by the author (M.M.)**

*Eotyrannus lengi*

Manuscript of osteology courtesy of Naish and Cau.

NGMC 2124

Personal examination and photos of specimen.

*Sinosauropteryx prima*

Photos of NIGP 127587 courtesy of Senter.

*Aniksosaurus darwini*

Photos of MTD-PV 1/6 and 1/41 courtesy of Jansma.

*Ornitholestes hermanni*

Personal examination of AMNH 619, photos of cast YPM 56681 courtesy of Senter.

*Santanaraptor placidus*

Photos of MN 4802-V courtesy of Bruno Campos.

*Pelecanimimus polyodon*

Photos of LH 7777.

*Archaeornithomimus asiaticus*

Personal examination of AMNH 6558, 6565-6570, 6576, 21786-21803, 21626-21627, 21884-21892 and 30240A-G.

*Archaeornithomimus? bissektensis*

Photos of CCMGE 613/12457 and 726/12457 courtesy of Averianov.

Tugrik parvicursorine

Personal examination of IGM 100/99.

*Shuvuuia deserti*

Personal examination of IGM 100/975 and 100/1276.

*Mononykus olecranus*

Photos of cast YPM 56693 courtesy of Senter.

*Beipiaosaurus inexpectus*

Photos of IVPP V11559 courtesy of Zanno.

*Alxasaurus elesitaiensis*

Photos of IVPP 88402a courtesy of Zanno.

*Enigmosaurus mongoliensis*

Photos of IGM 100/84 courtesy of Zanno.

*Segnosaurus galbinensis*

Photos of IGM 100/80-83 courtesy of Zanno.

*Protarchaeopteryx robusta*

Personal examination and photos of NGMC 2125.

*Caudipteryx zoui*

Personal examination and photos of NGMC 97-4-A and NGMC 97-9-A.

*Microvenator celer*

Personal examination of AMNH 3041.

*Chirostenotes pergracilis*

Photos of CMN 2367 courtesy of Senter.

*Anzu wyliei*

Photos of CM 78000 and 78001 courtesy of Headden.

*Kuszholia mengi*

Photos of ZIN PO 4602 courtesy of Averianov.

*Oviraptor philoceratops*

Personal examination and photos of AMNH 7517 courtesy of Senter.

*Citipati osmolskae*

Photos of IGM 100/978 courtesy of Mallison.

IGM 100/42

Photos of specimen.

*Rinchenia mongoliensis*

Photos of IGM 100/32a.

IGM 100/1128

Personal examination of specimen.

*Almas ukhaa*

Personal examination of IGM 100/1323.

*Hesperornithoides miessleri*

Scored by S.H via personal examination of WDC DML-001

*Mei long*

Photos of IVPP V12733.

*Sinovenator changii*

Photos of IVPP V12615.

*Saurornithoides mongoliensis*

Personal examination of AMNH 6516.

*Hulsanpes perlei*

Photos of ZPAL MgD-I/173 courtesy of Cau.

*Changyuraptor yangi*

Photos of HG B016 courtesy of Habib.

*Bambiraptor feinbergi*

Personal examination of AMNH 30556.

*Adasaurus mongoliensis*

Photos of IGM 100/20.

*Velociraptor mongoliensis*

Personal examination of AMNH 6515 and IGM 100/986.

*Dromaeosaurus albertensis*

Photos of AMNH 5356 courtesy of Senter.

*Unenlagia comahuensis*

Photos of cast YPM VP 56517 courtesy of Senter.

*Buitreraptor gonzalezorum*

Photos of MPCA 238, 245 and 471-C courtesy of Gianechini.

*Rahonavis ostromi*

Photos of cast YPM VP 56587 courtesy of Senter.

*Serikornis sungei*

Photos of PMOL-AB00200 courtesy of Cau.

*Aurornis xui*

Photos of YFGP-T5198 courtesy of Cau.

*Eosinopteryx brevipenna*

Photos of YFGP-T5197 courtesy of Cau.

*Anchiornis huxleyi*

Photo of undescribed skull.

*Jixiangornis orientalis*

Photos of YFGP-yb2 courtesy of Dyke.

*Bohaiornis guoi*

Photos of IVPP V17963 courtesy of Li.

**Institutional abbreviations**

AMNH American Museum of Natural History, New York, USA

BYU Brigham Young University Museum of Paleontology, Utah, USA

CAGS Chinese Academy of Geological Sciences, Beijing, China

CCMGE Chernyshev's Central Museum of Geological Exploration, Saint Petersburg, Russia

CM Collection Méchin, Vitrolles, France (*Variraptor*)

CM Carnegie Museum of Natural History, Pittsburgh, USA (*Anzu*)

CMN Canadian Museum of Nature, Ottawa, Canada

DINO Dinosaur National Monument, Jensen, USA

DGBU Department of Geology of Pusan National University, Pusan, South Korea

HG Paleontological Center, Bohai University, Jinzhou City, China

IGM Mongolian Institute of Geology, Ulaanbaatar, Mongolia

ISMD Institute for the Study of Mongolian Dinosaurs, Ulaanbaatar, Mongolia

IVPP Institute of Vertebrate Paleontology and Paleoanthropology, Beijing, China

LH Museo de Cuenca, Cuenca, Spain

LP Institut d'Estudis Illerdencs, Lleida, Spain

MCF PVPH Museo Municipal Carmen Funes, Plaza Huincul, Neuquén, Argentina

MDT-PV Museo Desiderio Torres -Paleovertebrados, Sarmiento, Argentina

MN Museu Nacional, Universidad Federal do Rio de Janeiro, Rio de Janeiro, Brazil

MNA Museum of Northern Arizona, Flagstaff, USA

MNN Musée National du Niger, Niamey, Niger

MOR Museum of the Rockies, Bozeman, USA

MPCA Museo Provincial Carlos Ameghino, Cipolletti, Argentina

MUCPv Museo de Geología y Paleontología de la Universidad Nacional del Comahue, Neuquén, Argentina

NGMC Geological Museum of China, Beijing, China

NHMUK Natural History Museum, London, United Kingdom

NIGP Nanjing Institute of Geology and Palaeontology, Nanjing, China

PMO Natural History Museum of the University of Oslo, Oslo, Norway

PMOL Palaeontological Museum of Liaoning, Shenyang, China

PVL Fundación-Instituto Miguel Lillo, Tucumán, Argentina

WDC Wyoming Dinosaur Center, Thermopolis, USA

YFGP Yizhou Fossil and Geology Park, Yizhou, China

YPM Yale-Peabody Museum of Natural History, New Haven, USA

ZIN Zoological Institute of the Russian Academy of Sciences, Saint Petersburg, Russia

ZPAL Institute of Paleobiology, Polish Academy of Sciences, Warsaw, Poland

**Supplementary references**

References that include TWiG character lists which were systematically analyzed here are in **bold**.

**Agnolin and Novas, 2011. Unenlagiid theropods: Are they members of the Dromaeosauridae (Theropoda, Maniraptora)? Anais da Academia Brasileira de Ciências. 83(1), 117-162. DOI: 10.1590/S0001-37652011000100008**

Agnolin and Novas, 2013. Avian ancestors: A review of the phylogenetic relationships of the theropods Unenlagiidae, Microraptoria, *Anchiornis* and Scansoriopterygidae. Springer Netherlands. 96 pp. DOI: 10.1007/978-94-007-5637-3_1

Agnolin, Powell, Novas and Kundrat, 2012. New alvarezsaurid (Dinosauria, Theropoda) from uppermost Cretaceous of north-western Patagonia with associated eggs. Cretaceous Research. 35, 33-56. DOI: 10.1016/j.cretres.2011.11.014

Aguillon Martinez, 2010. Fossil vertebrates from the Cerro del Pueblo Formation, Coahuila, Mexico, and the distribution of Late Campanian (Cretaceous) terrestrial vertebrate faunas. MS thesis, Dedman College Southern Methodist University. 135 pp.

Alifanov and Barsbold, 2009. *Ceratonykus oculatus* gen. et sp. nov., a new dinosaur (?Theropoda, Alvarezsauria) from the Late Cretaceous of Mongolia. Paleontological Journal. 43(1), 94-106. DOI: 10.1134/S0031030109010109

Allain, Vullo, Le Loeuff and Tournepiche, 2014. European ornithomimosaurs (Dinosauria, Theropoda): An undetected record. Geologica Acta. 12(2), 127-135. DOI : 10.1344/105.000002083

**Averianov and Sues, 2016. Troodontidae (Dinosauria: Theropoda) from the Upper Cretaceous of Uzbekistan. Cretaceous Research. 59, 98-110. DOI: 10.1016/j.cretres.2015.11.005**

**Azuma, Xu, Shibata, Kawabe, Miyata and Imai, 2016. A bizarre theropod from the Early Cretaceous of Japan highlighting mosaic evolution among coelurosaurians. Scientific Reports. 6, 20478. DOI: 10.1038/srep20478**

**Balanoff, Smaers and Turner, 2015. Brain modularity across the theropod-bird transition: Testing the influence of flight on neuroanatomical variation. Journal of Anatomy. 229(2), 204-214. DOI: 10.1111/joa.12403**

Barsbold, 1981. Toothless dinosaurs of Mongolia. Joint Soviet-Mongolian Paleontological Expedition Transactions. 15, 28-39.

Barsbold, 1988. A new Late Cretaceous ornithomimid from the Mongolia People’s Republic. Journal of Paleontology. 1988(1), 122-125.

Barsbold, Osmolska and Kurzanov, 1987. On a new troodontid (Dinosauria, Theropoda) from the Early Cretaceous of Mongolia. Acta Palaeontologica Polonica. 32(1-2), 121-132.

Baumel and Witmer, 1993. Osteologia. In Baumel, King, Breazile, Evans and Berge (eds.). Handbook of Avian Anatomy: Nomina Anatomica Avium. Second Edition. Publications of the Nuttall Ornithological Club, No. 23. 45-132.

Bever and Norell, 2009. The perinate skull of *Byronosaurus* (Troodontidae) with observations on the cranial ontogeny of paravian theropods. American Museum Novitates. 3657, 51 pp. DOI: 10.1206/650.1

Brochu, 2003. Osteology of *Tyrannosaurus rex*: Insights from a nearly complete skeleton and high-resolution computed tomographic analysis of the skull. Society of Vertebrate Paleontology Memoir. 7, 138 pp. DOI: 10.1080/02724634.2003.10010947

Brusatte, 2013. The phylogeny of basal coelurosaurian theropods (Archosauria: Dinosauria) and patterns of morphological evolution during the dinosaur-bird transition. PhD thesis, Columbia University. 944 pp.

Brusatte, Lloyd, Wang and Norell, 2014. Gradual assembly of avian body plan culminated in rapid rates of evolution across the dinosaur-bird transition. Current Biology. 24(20), 2386-2392. DOI: 10.1016/j.cub.2014.08.034

Brusatte, Averianov, Sues, Muir and Butler, 2016. New tyrannosaur from the mid-Cretaceous of Uzbekistan clarifies evolution of giant body sizes and advanced senses in tyrant dinosaurs. Proceedings of the National Academy of Sciences. 113(13), 3447-3452. DOI: 10.1073/pnas.1600140113

Burnham, 2004. New information on *Bambiraptor feinbergi* (Theropoda: Dromaeosauridae) from the Late Cretaceous of Montana. In Currie, Koppelhus, Shugar and Wright (eds.). Feathered Dragons. Studies on the transition from dinosaurs to birds. Indiana University Press. 67-111.

**Calvo, Porfiri and Kellner, 2004. On a new maniraptoran dinosaur (Theropoda) from the Upper Cretaceous of Neuquén, Patagonia, Argentina. Arquivos do Museo Nacional. 62, 549-566.**

Carpenter, 2002. Forelimb biomechanics of nonavian theropod dinosaurs in predation. Senckenbergiana lethaea. 82(1), 59-76. DOI: 10.1007/BF03043773

Carpenter and Smith, 2001. Forelimb osteology and biomechanics of *Tyrannosaurus rex*. In Tanke and Carpenter (eds.). Mesozoic Vertebrate Life. Indiana University Press. 90-116.

Carpenter, Miles and Cloward, 2005a. New small theropod from the Upper Jurassic Morrison Formation of Wyoming. In Carpenter (ed.). The Carnivorous Dinosaurs. Indiana University Press. 23-48.

Carr, 1999. Craniofacial ontogeny in Tyrannosauridae (Dinosauria, Coelurosauria). Journal of Vertebrate Paleontology. 19(3), 497-520. DOI: 10.1080/02724634.1999.10011161

Carr, 2005. Phylogeny of Tyrannosauroidea (Dinosauria: Coelurosauria) with special reference to North American forms. PhD thesis. University of Toronto. 1170 pp.

Carrano, Benson and Sampson, 2012. The phylogeny of Tetanurae (Dinosauria: Theropoda). Journal of Systematic Palaeontology. 10(2), 211-300. DOI: 10.1080/14772019.2011.630927

Case, Martin and Reguero, 2007. A dromaeosaur from the Maastrichtian of James Ross Island and the Late Cretaceous Antarctic dinosaur fauna. in Cooper and Raymond (eds). Antarctica: A Keystone in a Changing World – Online Proceedings of the 10th ISAES X. USGS Open-File Report 2007-1047, Short Research Paper 083, 4 pp. DOI: 10.3133/ofr20071047SRP083

Cau, 2018. The assembly of the avian body plan: A 160-million-year long process. Bollettino della Società Paleontologica Italiana. 57(1), 1-25. DOI: 10.4435/BSPI.2018.01

Cau and Arduini, 2008. *Enantiophoenix electrophyla* gen. et sp. nov. (Aves, Enantiornithes) from the Upper Cretaceous (Cenomanian) of Lebanon and its phylogenetic relationships. Atti della Societa Italiana di Scienze Naturali e del Museo ivico di Storia Naturale in Milano. 149(II), 293-324.

Cau, Brougham and Naish, 2015. The phylogenetic affinities of the bizarre Late Cretaceous Romanian theropod *Balaur bondoc* (Dinosauria, Maniraptora): Dromaeosaurid or flightless bird? PeerJ. 3, e1032. 10.7717/peerj.1032

Cau, Beyrand, Voeten, Fernandez, Tafforeau, Stein, Barsbold, Tsogtbaatar, Currie and Godefroit, 2017. Synchrotron scanning reveals amphibious ecomorphology in a new clade of bird-like dinosaurs. Nature. 552, 395-399. DOI: 10.1038/nature24679

Chiappe, 1995a. The phylogenetic position of the Cretaceous birds of Argentina: Enantiornithes and *Patagopteryx deferrariisi*. Courier Forschungsinstitut-Senckenberg. 181, 55-63.

Chiappe, 2001. Phylogenetic relationships among basal birds. In Gauthier and Gall (eds.). New Perspectives on the Origin and Early Evolution of Birds: Proceedings of the International Symposium in Honor of John H. Ostrom. 125-139.

Chiappe, 2002. Basal bird phylogeny: Problems and solutions. In Chiappe and Witmer (eds.). Mesozoic birds: Above the heads of dinosaurs. Berkeley: University of California Press. 448-472.

Chiappe, Norell and Clark, 1998. The skull of a relative of the stem-group bird *Mononykus*. Nature. 392, 275-278. DOI: 10.1038/32642

Chiappe, Norell and Clark, 2002. The Cretaceous, short-armed Alvarezsauridae, *Mononykus* and its kin. In Chiappe and Witmer (eds.). Mesozoic Birds: Above the Heads of Dinosaurs. Berkeley: University of California Press. 87-120.

Chiappe, Ji, Ji and Norell, 1999. Anatomy and systematics of the Confuciusornithidae (Theropoda: Aves) from the Late Mesozoic of Northeastern China. Bulletin of American Museum of Natural History. 242, 1-89. DOI: 10.1642/0004-8038(2000)117[0836:AASOTC]2.0.CO;2

Choiniere, 2010. Anatomy and systematics of coelurosaurian theropods from the Late Jurassic of Xinjiang, China, with comments on forelimb evolution in Theropoda. PhD thesis. George Washington University. 994 pp.

Choiniere, Forster and de Klerk, 2012. New information on *Nqwebasaurus thwazi*, a coelurosaurian theropod from the Early Cretaceous (Hauteriverian?) Kirkwood Formation in South Africa. Journal of African Earth Sciences. 71-72, 1-17. DOI: 10.1016/j.jafrearsci.2012.05.005

Choiniere, Clark, Norell and Xu, 2014. Cranial osteology of *Haplocheirus sollers* Choiniere et al., 2010 (Theropoda: Alvarezsauroidea). American Museum Novitates. 3816, 44 pp. DOI: 10.1206/3816.1

Choiniere, Xu, Clark, Forster, Guo and Han, 2010. A basal alvarezsauroid theropod from the early Late Jurassic of Xinjiang, China. Science. 327, 571-574. DOI: 10.1126/science.1182143

Chure, 2000. A new species of *Allosaurus* from the Morrison Formation of Dinosaur National Monument (Utah-Colorado) and a revision of the theropod family Allosauridae. PhD thesis. Columbia University. 964 pp.

Clark, Maryanska and Barsbold, 2004. Therizinosauroidea. In Weishampel, Dodson and Osmolska (eds). The Dinosauria Second Edition. University of California Press. 151-164.

**Clark, Norell and Makovicky, 2002. Cladistic approaches to the relationships of birds to other theropod dinosaurs. In Chiappe and Witmer (eds.). Mesozoic Birds: Above the Heads of Dinosaurs. University of California Press. 31-64.**

Clarke, 2002. The morphology and systematic position of *Ichthyornis* Marsh and the phylogenetic relationships of basal Ornithurae. PhD thesis, Yale University. 532 pp.

Clarke, 2004. Morphology, phylogenetic taxonomy, and systematics of *Ichthyornis* and *Apatornis* (Avialae: Ornithurae). Bulletin of the American Museum of Natural History. 286, 1-179. DOI: 10.1206/0003-0090(2004)286<0001:MPTASO>2.0.CO;2

Clarke and Norell, 2002. The morphology and phylogenetic position of *Apsaravis ukhaana* from the Late Cretaceous of Mongolia. American Museum Novitates. 3387, 46 pp. DOI: 10.1206/0003-0082(2002)387<0001:TMAPPO>2.0.CO;2

Coria, Currie and Carabajal, 2006. A new abelisauroid theropod from northwestern Patagonia. Canadian Journal of Earth Sciences. 43(9), 1283-1289. DOI: 10.1139/e06-025

Cracraft, 1986. The origin and early diversification of birds. Paleobiology. 12, 383-399. DOI: 10.1017/S0094837300003122

**Csiki, Vremir, Brusatte and Norell, 2010. An aberrant island-dwelling theropod dinosaur from the Late Cretaceous of Romania. Proceedings of the National Academy of Sciences. 107(35), 15357-15361. DOI: 10.1073/pnas.1006970107**

Currie, 1987. Bird-like characteristics of the jaws and teeth of troodontid theropods (Dinosauria, Saurischia). Journal of Vertebrate Paleontology. 7, 72-81. DOI: 10.1080/02724634.1987.10011638

Currie, 1995. New information on the anatomy and relationships of *Dromaeosaurus albertensis* (Dinosauria: Theropoda). Journal of Vertebrate Paleontology. 15(3), 576-591. DOI: 10.1080/02724634.1995.10011250

Currie, 2003. Cranial anatomy of tyrannosaurid dinosaurs from the Late Cretaceous of Alberta, Canada. Acta Palaeontologica Polonica. 48(2), 191-226.

Currie and Dong, 2001. New information on Cretaceous troodontids (Dinosauria, Theropoda) from the People's Republic of China. Canadian Journal of Earth Sciences. 38(12), 1753-1766. DOI: 10.1139/e01-065

Currie and Padian, 1997a. Avetheropoda. In Currie and Padian (eds.). Encyclopedia of Dinosaurs. Academic Press, New York. 39.

Currie and Padian, 1997b. Deinonychosauria. In Currie and Padian (eds.). Encyclopedia of Dinosaurs. Academic Press, New York. 166-167.

Currie and Zhao, 1994. A new carnosaur (Dinosauria, Theropoda) from the Jurassic of Xinjiang, People's Republic of China. Canadian Journal of Earth Sciences. 30(10), 2037-2081. DOI: 10.1139/e93-179

Currie, Rigby and Sloan, 1990. Theropod teeth from the Judith River Formation of southern Alberta, Canada. in Carpenter and Currie (eds.). Dinosaur Systematics: Perspectives and Approaches. Cambridge University Press. 107-125.

**Dal Sasso and Maganuco, 2011. *Scipionyx samniticus* (Theropoda: Compsognathidae) from the Lower Cretaceous of Italy: Osteology, ontogenetic assessment, phylogeny, soft tissue anatomy, taphonomy, and palaeobiology. Memorie della Società Italiana di Scienze Naturali e del Museo Civico di Storia Naturale di Milano. 281 pp.**

**Dececchi, Larsson and Hone, 2012. *Yixianosaurus longimanus* (Theropoda: Dinosauria) and its bearing on the evolution of Maniraptora and ecology of the Jehol fauna. Vertebrata PalAsiatica. 59(2) 111-139.**

DeCourten and Russell, 1985. A specimen of *Ornithomimus velox* (Theropoda, Ornithomimidae) from the terminal Cretaceous Kaiparowits Formation of southern Utah. Journal of Paleontology. 59(5), 1091-1099.

Denton, Nesbitt, Wolfe and Holtz, 2004. A new small theropod dinosaur from the Moreno Hill Formation (Turonian, Upper Cretaceous) of New Mexico. Journal of Vertebrate Paleontology. 24(3), 302A.

**DePalma, Burnham, Martin, Larson and Bakker, 2015. The first giant raptor (Theropoda: Dromaeosauridae) from the Hell Creek Formation. Paleontological Contributions. 14, 16 pp. DOI: 10.17161/paleo.1808.18764**

DigiMorph Staff, 2001. "*Chauna chavaria*" (On-line), Digital Morphology. Accessed January 14, 2018 at http://digimorph.org/specimens/Chauna_chavaria/.

Domínguez Alonso, Milner, Ketcham, Cookson and Rowe, 2004. The avian nature of the brain and inner ear of *Archaeopteryx*. Nature. 430, 666-669. DOI: 10.1038/nature02706

Dong, 1997. On small theropods from Mazongshan Area, Gansu Province, China. In Dong (ed). Sino-Japanese Silk Road Dinosaur Expedition. China Ocean Press, Beijing. 13-18.

Dong and Currie, 1996. On the discovery of an oviraptorid skeleton on a nest of eggs at Bayan Mandahu, Inner Mongolia, People's Republic of China. Canadian Journal of Earth Sciences. 33(4), 631-636. DOI: 10.1139/e96-046

Dufeau, 2003. The cranial anatomy of the theropod dinosaur *Shuvuuia deserti* (Coelurosauria: Alvarezsauridae), and its bearing upon coelurosaurian phylogeny. Masters thesis. The University of Texas at Austin. 275 pp.

Ely and Case, 2016. A basal deinonychosaur from the Early Maastrichtian, Antarctic peninsula and the biostratigraphy of the latest Cretaceous dinosaur fauna of Antarctica. Journal of Vertebrate Paleontology. Program and Abstracts, 130.

Elzanowski, 1999. A comparison of the jaw skeleton in theropods and birds, with a description of the palate in the Oviraptoridae. Smithsonian Contributions to Paleobiology. 89, 311-323.

**Evans, Larson and Currie, 2013. A new dromaeosaurid (Dinosauria: Theropoda) with Asian affinities from the latest Cretaceous of North America. Naturwissenschaften. 100(11), 1041-1049. DOI: 10.1007/s00114-013-1107-5**

Farlow, Brinkman and Abler, 1991. Size, shape, and serration density of theropod dinosaur lateral teeth. Modern Geology. 16, 161-198.

Fisher, 1940. The occurrence of vestigial claws on the wings of birds. American Midland Naturalist. 23(1), 234-243. DOI: 10.2307/2485270

Foth and Rauhut, 2017. Re-evaluation of the Haarlem *Archaeopteryx* and the radiation of maniraptoran theropod dinosaurs. BMC Evolutionary Biology. 17:236. DOI: 10.1186/s12862-017-1076-y

Foth, Tischlinger and Rauhut, 2014. New specimen of *Archaeopteryx* provides insights into the evolution of pennaceous feathers. Nature. 511, 79-82. DOI: 10.1038/nature13467

Gao, Li, Wei, Pak and Pak, 2009. Early Cretaceous birds and pterosaurs from the Sinuiju series, and geographic extension of the Jehol biota into the Korean peninsula. Journal of the Paleontological Society of Korea. 25(1), 57-61.

**Gao, Morschhauser, Varricchio, Liu and Zhao, 2012. A second soundly sleeping dragon: New anatomical details of the Chinese troodontid *Mei long* with implications for phylogeny and taphonomy. PLoS ONE. 7(9), e45203. DOI: 10.1371/journal.pone.0045203**

Gauthier, 1986. Saurischian monophyly and the origin of birds. Memoirs of the Californian Academy of Sciences. 8, 1-55.

Gauthier and de Queiroz, 2001. Feathered dinosaurs, flying dinosaurs, crown dinosaurs, and the name 'Aves'. In Gauthier and Gall (eds.). New Perspectives on the Origin and Early Evolution of Birds: Proceedings of the International Symposium in Honor of John H. Ostrom. 7-41.

Gianechini, Agnolín and Ezcurra, 2011. A reassessment of the purported venom delivery system of the bird-like raptor *Sinornithosaurus*. Paläontologische Zeitschrift. 85, 103-107. DOI: 10.1007/s12542-010-0074-9

Gianechini, Makovicky and Apesteguía, 2017. The cranial osteology of *Buitreraptor gonzalezorum* Makovicky, Apesteguía, and Agnolín, 2005 (Theropoda, Dromaeosauridae), from the Late Cretaceous of Patagonia, Argentina. Journal of Vertebrate Paleontology. e1255639. DOI: 10.1080/02724634.2017.1255639

Gianechini, Makovicky, Apesteguía and Cerda, 2018. Postcranial skeletal anatomy of the holotype and referred specimens of *Buitreraptor gonzalezorum* Makovicky, Apesteguía and Agnolín 2005 (Theropoda, Dromaeosauridae), from the Late Cretaceous of Patagonia. PeerJ. 6:e4558. DOI: 10.7717/peerj.4558

Gilmore, 1920. Osteology of the carnivorous Dinosauria in the United States National Museum, with special reference to the genera *Antrodemus* (*Allosaurus*) and *Ceratosaurus*. Bulletin of the United States National Museum. 110, 1-154. DOI: 10.5962/bhl.title.61883

Gishlick, 2002. The functional morphology of the forelimb of *Deinonychus antirrhopus* and its importance for the origin of avian flight. PhD thesis, Yale University, 142 pp.

Godefroit, Cau, Hu, Escuillie, Wu and Dyke, 2013. A Jurassic avialan dinosaur from China resolves the early phylogenetic history of birds. Nature. 498, 359-362. DOI: 10.1038/nature12168

**Gohlich and Chiappe, 2006. A new carnivorous dinosaur from the Late Jurassic Solnhofen archipelago. Nature. 440, 329-332. DOI: 10.1038/nature04579**

Goloboff and Catalano, 2016. TNT version 1.5, including a full implementation of phylogenetic morphometrics. Cladistics. 32(3), 221-238. DOI: 10.1111/cla.12160

**Han, Chiappe, Ji, Habib, Turner, Chinsamy, Liu and Han, 2014. A new raptorial dinosaur with exceptionally long feathering provides insights into dromaeosaurid flight performance. Nature Communications. 5, 4382. DOI: 10.1038/ncomms5382**

Harris, Lamanna, You, Ji and Ji, 2006. A second enantiornithean (Aves: Ornithothoraces) wing from the Early Cretaceous Xiagou Formation near Changma, Gansu Province, People’s Republic of China. Canadian Journal of Earth Sciences. 43, 547-554. DOI: 10.1139/e06-007

Hendrickx, Hartman and Mateus, 2015. An overview of non-avian theropod discoveries and classification. PalArch's Journal of Vertebrate Palaeontology. 12(1), 1-73.

Holtz, 1996. Phylogenetic taxonomy of the Coelurosauria (Dinosauria: Theropoda). Journal of Paleontology. 70, 536-538. DOI: 10.1017/S0022336000038506

Holtz, 2004. Tyrannosauroidea. In Weishampel, Dodson and Osmolska (eds.). The Dinosauria Second Edition. University of California Press. 111-136.

Holtz and Osmolska, 2004. Saurischia. In Weishampel, Dodson and Osmolska (eds.). The Dinosauria Second Edition. University of California Press. 21-24.

Holtz, Molnar and Currie, 2004. Basal Tetanurae. In Weishampel, Dodson and Osmolska (eds.). The Dinosauria Second Edition. University of California Press. 71-110.

**Hu, Hou, Zhang and Xu, 2009. A pre-*Archaeopteryx* troodontid theropod from China with long feathers on the metatarsus. Nature. 461, 640-643. DOI: 10.1038/nature08322**

**Hu, Clarke, Eliason, Qiu, Li, Shawkey, Zhao, D'Alba, Jiang and Xu, 2018. A bony-crested Jurassic dinosaur with evidence of iridescent plumage highlights complexity in early paravian evolution. Nature Communications. 9, 217. DOI: doi.org/10.1038/s41467-017-02515-y**

Hurum, Roberts, Dyke, Grundvåg, Nakrem, Midtkandal, Śliwińska and Olaussen, 2016. Bird or maniraptoran dinosaur? A femur from the Albian strata of Spitsbergen. Palaeontologia Polonica. 67, 137-147. DOI: 10.4202/pp.2016.67_137

Hutchinson, 2001a. The evolution of pelvic osteology and soft tissues on the line to extant birds (Neornithes). Zoological Journal of the Linnean Society. 131, 123-168. DOI: 10.1006/zjls.2000.0254

Hutchinson, 2001b. The evolution of femoral osteology and soft tissues on the line to extant birds (Neornithes). Zoological Journal of the Linnean Society. 131, 169-197. DOI: 10.1006/zjls.2000.0267

**Hwang, 2007. Phylogenetic patterns of enamel microstructure in dinosaur teeth. PhD thesis. Columbia University. 274 pp.**

**Hwang, Norell, Ji and Gao, 2004. A large compsognathid from the Early Cretaceous Yixian Formation of China. Journal of Systematic Palaeontology. 2(1), 13-30. DOI: 10.1017/S1477201903001081**

**Hwang, Norell, Qiang and Keqin, 2002. New specimens of *Microraptor zhaoianus* (Theropoda: Dromaeosauridae) from northeastern China. American Museum Novitates. 3381, 1-44. DOI: 10.1206/0003-0082(2002)381<0001:NSOMZT>2.0.CO;2**

Jensen, 1981. Another look at *Archaeopteryx* as the "oldest" bird. Encyclia, The Journal of the Utah Academy of Sciences, Arts, and Letters. 58, 109-128.

Ji and Ji, 1997. Advances in the study of the avian *Sinosauropteryx prima*. Chinese Geology. 242, 30-32.

Ji, Norell, Gao, Ji and Ren, 2001. The distribution of integumentary structures in a feathered dinosaur. Nature. 410(6832), 1084-1088. DOI: 10.1038/35074079

**Ji, Norell, Makovicky, Gao, Ji and Yuan, 2003. An early ostrich dinosaur and implications for ornithomimosaur phylogeny. American Museum Novitates. 3420, 19 pp.**

**Jiang, 2011. A new discovery of *Anchiornis* from western of Liaoning, China. Masters thesis, Chengdu University of Technology. 101 pp.**

Kim, Gishlick and Tsuihiji, 2005. The first non-avian maniraptoran skeletal remains from the Lower Cretaceous of Korea. Cretaceous Research. 26, 299-306. DOI: 10.1016/j.cretres.2005.01.001

**Kirkland, Zanno, Sampson, Clark and DeBlieux, 2005. A primitive therizinosauroid dinosaur from the Early Cretaceous of Utah. Nature. 435, 84-87. DOI: 10.1038/nature03468**

**Kobayashi, 2004. Asian ornithomimosaurs. PhD Thesis, Southern Methodist University. 340 pp.**

Kobayashi, 2008. Phylogenetic position of Ornithomimosauria in Coelurosauria with comments on the relationship of ornithomimosaurs and alvarezsaurids. Journal of Fossil Research. 41, 25-32.

Kobayashi and Barsbold, 2002. A new primitive ornithomimosaur from the Early Cretaceous of Mongolia and the early evolution of Ornithomimosauria. Journal of Vertebrate Paleontology. 22(3), 75A.

Kobayashi and Barsbold, 2005b. Reexamination of a primitive ornithomimosaur, *Garudimimus brevipes* Barsbold, 1981 (Dinosauria: Theropoda), from the Late Cretaceous of Mongolia. Canadian Journal of Earth Sciences. 42(9), 1501-1521. DOI: 10.1139/e05-044

Kobayashi and Barsbold, 2006. Ornithomimids from the Nemegt Formation of Mongolia. Journal of the Paleontological Society of Korea. 22(1), 195-207.

Kubota and Barsbold, 2007. New dromaeosaurid (Dinosauria Theropoda) from the Upper Cretaceous Bayanshiree Formation of Mongolia. Journal of Vertebrate Paleontology. 27(3), 102A.

Kundrát, Cruickshank, Manning and Nudds, 2007. Embryos of therizinosauroid theropods from the Upper Cretaceous of China: Diagnosis and analysis of ossification patterns. Acta Zoologica. 89(3), 231-251. DOI: 10.1111/j.1463-6395.2007.00311.x

Lamanna, You, Harris, Chiappe, Ji, Lü and Ji, 2006. A partial skeleton of an enantiornithine bird from the Early Cretaceous of Northwestern China. Acta Palaeontologica Polonica. 51(3), 423-434.

Lee, Barsbold, Currie, Kobayashi, Lee, Godefroit, Escuillie and Tsogtbaatar, 2014. Resolving the long-standing enigmas of a giant ornithomimosaur *Deinocheirus mirificus*. Nature. 515, 257-260. DOI: 10.1038/nature13874

Lefevre, Hu, Escuillie, Dyke and Godefroit, 2014. A new long-tailed basal bird from the Lower Cretaceous of north-eastern China. Biological Journal of the Linnean Society. 113, 790-804. DOI: 10.1111/bij.12343

Li, You and Zhang, 2008. A new specimen of *Suzhousaurus megatherioides* (Dinosauria: Therizinosauroidea) from the Early Cretaceous of northwestern China. Canadian Journal of Earth Sciences. 45(7), 769-779. DOI: 10.1139/E08-021

**Li, Norell, Gao, Smith and Makovicky, 2010. A longirostrine tyrannosauroid from the Early Cretaceous of China. Proceedings of the Royal Society B. 277(1679), 183-190. DOI: 10.1098/rspb.2009.0249**

Longrich, 2002. Systematics of *Sinosauropteryx*. Journal of Vertebrate Paleontology. 22(3), 80A.

Longrich, 2009. An ornithurine-dominated avifauna from the Belly River Group (Campanian, Upper Cretaceous) of Alberta, Canada. Cretaceous Research. 30(1), 161-177. DOI: 10.1016/j.cretres.2008.06.007

Longrich and Currie, 2009. *Albertonykus borealis*, a new alvarezsaur (Dinosauria: Theropoda) from the Early Maastrichtian of Alberta, Canada: Implications for the systematics and ecology of the Alvarezsauridae. Cretaceous Research. 30(1), 239-252. DOI: 10.1016/j.cretres.2008.07.005

**Lu, 2004. Oviraptorid dinosaurs from southern China. PhD thesis, Southern Methodist University. 249 pp.**

**Lu and Brusatte, 2015. A large, short-armed, winged dromaeosaurid (Dinosauria: Theropoda) from the Early Cretaceous of China and its implications for feather evolution. Scientific Reports. 5, 11775. DOI: doi.org/10.1038/srep11775**

Lu, Tomida, Azuma, Dong and Lee, 2004. New oviraptorid dinosaur (Dinosauria: Oviraptorosauria) from the Nemegt Formation of southwestern Mongolia. Bulletin of the National Science Museum, Tokyo, Series C. 30, 95-130.

Mader and Bradley, 1989. A redescription and revised diagnosis of the syntypes of the Mongolian tyrannosaur *Alectrosaurus olseni*. Journal of Vertebrate Paleontology. 9(1), 41-55. DOI: 10.1080/02724634.1989.10011737

Madsen, 1976. *Allosaurus fragilis*: A revised osteology. Utah Geological and Mineral Survey Bulletin. 109, 1-163.

Makovicky, 1995. Phylogenetic aspects of the vertebral morphology of Coelurosauria (Dinosauria: Theropoda). Masters thesis. University of Copenhagen. 311 pp.

**Makovicky and Norell, 2004. Troodontidae. In Weishampel, Dodson and Osmolska (eds.). The Dinosauria Second Edition. University of California Press. 184-195.**

Makovicky and Sues, 1998. Anatomy and phylogenetic relationships of the theropod dinosaur *Microvenator celer* from the Lower Cretaceous of Montana. American Museum Novitates. 3240, 1-27.

**Makovicky, Apesteguía and Agnolín, 2005. The earliest dromaeosaurid theropod from South America. Nature. 437, 1007-1011. DOI: 10.1038/nature03996**

**Makovicky, Apesteguia and Gianechini, 2012. A new coelurosaurian theropod from the La Buitrera fossil locality of Río Negro, Argentina. Fieldiana Life and Earth Sciences. 5, 90-98. DOI: 10.3158/2158-5520-5.1.90**

**Makovicky, Kobayashi and Currie, 2004. Ornithomimosauria. In Weishampel, Dodson and Osmolska (eds.). The Dinosauria Second Edition. University of California Press. 137-150.**

**Makovicky, Norell, Clark and Rowe, 2003. Osteology and relationships of *Byronosaurus jaffei* (Theropoda: Troodontidae). American Museum Novitates. 3402, 1-32.**

**Makovicky, Li, Gao, Lewin, Erickson and Norell, 2010. A giant ornithomimosaur from the Early Cretaceous of China. Proceedings of the Royal Society B. 277, 191-198. DOI: 10.1098/rspb.2009.0236**

**Martinelli and Vera, 2007. *Achillesaurus manazzonei*, a new alvarezsaurid theropod (Dinosauria) from the Late Cretaceous Bajo de la Carpa Formation, Río Negro Province, Argentina. Zootaxa. 1582, 1-17. DOI: 10.11646/zootaxa.1582.1.1**

Martyniuk, 2012. A Field Guide to Mesozoic Birds and Other Winged Dinosaurs. Vernon, New Jersey. Pan Aves. 189 pp.

Maryanska, Osmolska and Wolsan, 2002. Avialan status for Oviraptorosauria. Acta Palaeontologica Polonica. 47(1), 97-116.

**Mayr, Pohl and Peters, 2005. A well-preserved *Archaeopteryx* specimen with theropod features. Science. 310, 1483-1486. DOI: 10.1126/science.1120331**

**Nesbitt, Clarke, Turner and Norell, 2011. A small alvarezsaurid from the Eastern Gobi Desert offers insight into evolutionary patterns in the Alvarezsauroidea. Journal of Vertebrate Paleontology. 31(1), 144-153. DOI: 10.1080/02724634.2011.540053**

Nicholls and Russell, 1985. Structure and function of the pectoral girdle and forelimb of *Struthiomimus altus* (Theropoda: Ornithomimidae). Palaeontology. 28, 643-677.

Norell and Clarke, 2001. Fossil that fills a critical gap in avian evolution. Nature. 409, 181-184. DOI: 10.1038/35051563

**Norell and Makovicky, 2004. Dromaeosauridae. In Weishampel, Dodson and Osmólska (eds.). The Dinosauria Second Edition. University of California Press. 196-209.**

**Norell, Clark and Makovicky, 2001. Phylogenetic relationships among coelurosaurian theropods. In Gauthier and Gall (eds.). New Perspectives on the Origin and Early Evolution of Birds: Proceedings of the International Symposium in Honor of John H. Ostrom. 49-67.**

**Norell, Clark, Turner, Makovicky, Barsbold and Rowe, 2006. A new dromaeosaurid theropod from Ukhaa Tolgod (Omnogov, Mongolia). American Museum Novitates. 3545, 51 pp.**

Novas, 1997. Anatomy of *Patagonykus puertai* (Theropoda, Avialae, Alvarezsauridae), from the Late Cretaceous of Patagonia. Journal of Vertebrate Paleontology. 17(1), 137-166. DOI: 10.1080/02724634.1997.10010959

**Novas and Pol, 2005. New evidence on deinonychosaurian dinosaurs from the Late Cretaceous of Patagonia. Nature. 433, 858-861. DOI: 10.1038/nature03285**

Novas and Puerta, 1997. New evidence concerning avian origins from the Late Cretaceous of Patagonia. Nature. 387, 390-392. DOI: 10.1038/387390a0

**Novas, Ezcurra and Lecuona, 2008. *Orkoraptor burkei* nov. gen. et sp., a large theropod from the Maastrichtian Pari Aike Formation, southern Patagonia, Argentina. Cretaceous Research. 29, 468-480. DOI: 10.1016/j.cretres.2008.01.001**

Novas, Agnolin, Ezcurra, Porfiri and Canale, 2013. Evolution of the carnivorous dinosaurs during the Cretaceous: The evidence from Patagonia. Cretaceous Research. 45, 174-215. DOI: 10.1016/j.cretres.2013.04.001

**Novas, Ezcurra, Agnolin, Pol and Ortiz, 2012. New Patagonian Cretaceous theropod sheds light about the early radiation of Coelurosauria. Revista del Museo Argentino de Ciencias Naturales. 14(1), 57-81.**

**Novas, Pol, Canale, Porfiri and Calvo, 2009. A bizarre Cretaceous theropod dinosaur from Patagonia and the evolution of Gondwanan dromaeosaurids. Proceedings of the Royal Society B. 276(1659), 1101-1107. DOI: 10.1098/rspb.2008.1554**

O'Connor and Forster, 2010. A Late Cretaceous (Maastrichtian) avifauna from the Maevarano Formation, Madagascar. Journal of Vertebrate Paleontology. 30(4), 1178-1201. DOI: 10.1080/02724634.2010.483544

**O'Connor and Sullivan, 2014. Reinterpretation of the Early Cretaceous maniraptoran (Dinosauria: Theropoda) *Zhongornis haoae* as a scansoriopterygid-like non-avian, and morphological resemblances between scansoriopterygids and basal oviraptorosaurs. Vertebrata PalAsiatica. 52(1), 3-30.**

O'Connor, Wang, Chiappe, Gao, Meng, Cheng and Liu, 2009. Phylogenetic support for a specialized clade of Cretaceous enantiornithine birds with information from a new species. Journal of Vertebrate Paleontology. 29(1), 188-204. DOI: 10.1080/02724634.2009.10010371

O'Connor, Zheng, Sullivan, Chuong, Wang, Wang, Zhang and Zhou, 2015. Evolution and functional significance of derived sternal ossification patterns in ornithothoracine birds. Journal of Evolutionary Biology. 28(8), 1550-1567. DOI: 10.1111/jeb.12675

Osmólska, Currie and Barsbold, 2004. Oviraptorosauria. In Weishampel, Dodson and Osmolska (eds.). The Dinosauria (second edition). University of California Press, Berkeley. 165-183.

Osmólska, Roniewicz and Barsbold, 1972. A new dinosaur, *Gallimimus bullatus* n. gen., n. sp. (Ornithomimidae) from the Upper Cretaceous of Mongolia. Palaeontologica Polonica. 27, 103-143.

Ostrom, 1969. Osteology of *Deinonychus antirrhopus*, an unusual theropod from the Lower Cretaceous of Montana. Bulletin of the Peabody Museum of Natural History. 30, 165 pp.

Padian, 2004. Basal Avialae. In Weishampel, Dodson and Osmolska (eds.). The Dinosauria Second Edition. University of California Press. 210-231.

Padian, Hutchinson and Holtz, 1997. Phylogenetic definitions and nomenclature of the major taxonomic categories of the theropod dinosaurs. Journal of Vertebrate Paleontology. 17(3), 68A. DOI: 10.1080/02724634.1997.10011028

Padian, Hutchinson and Holtz, 1999. Phylogenetic definitions and nomenclature of the major taxonomic categories of the carnivorous Dinosauria (Theropoda). Journal of Vertebrate Paleontology. 19(1), 69-80. DOI: 10.1080/02724634.1999.10011123

Parks, 1928b. *Struthiomimus samueli*, a new species of Ornithomimidae from the Belly River Formation of Alberta. University of Toronto Studies, Geology Series. 26, 1-24.

Pei, 2015. New paravian fossils from the Mesozoic of east Asia and their bearing on the phylogeny of the Coelurosauria. PhD thesis, Columbia University. 545 pp.

Pei, Li, Meng, Norell and Gao, 2017. New specimens of *Anchiornis huxleyi* (Theropoda: Paraves) from the Late Jurassic of northeastern China. Bulletin of the American Museum of Natural History. 411, 66 pp. DOI: 10.1206/0003-0090-411.1.1

Perez-Moreno, Sanz, Sudre and Sige, 1993. A theropod dinosaur from the Lower Cretaceous of southern France. Revue de Paleobiologie. 7, 173-188. DOI: 10.1038/370363a0

Perez-Moreno, Sanz, Buscalioni, Moratalla, Ortega and Rasskin-Gutman, 1994. A unique multitoothed ornithomimosaur dinosaur from the Lower Cretaceous of Spain. Nature. 370, 363-367.

Perle, Norell and Clark, 1999. A new maniraptoran theropod - *Achillobator giganticus* (Dromaeosauridae) - from the Upper Cretaceous of Burkhant, Mongolia. Contribution no. 101 of the Mongolian-American Paleontological Project. 1-105.

Perle, Chiappe, Barsbold, Clark and Norell, 1994. Skeletal morphology of *Mononykus olecranus* (Theropoda: Avialae) from the Late Cretaceous of Mongolia. American Museum Novitates. 3105, 1-29.

**Prieto-Marquez, Bolortsetseg and Horner, 2012. A diminutive deinonychosaur (Dinosauria: Theropoda) from the Early Cretaceous of Oosh (Ovorkhangai, Mongolia). Alcheringa. 36(1), 117-136. DOI: 10.1080/03115518.2011.590401**

Pu, Chang, Lu, Wu, Xu, Zhang and Jia, 2013. A new juvenile specimen of *Sapeornis* (Pygostylia: Aves) from the Lower Cretaceous of northeast China and allometric scaling of this basal bird. Paleontological Research. 17(1), 27-38. DOI: 10.2517/1342-8144-17.1.27

**Pu, Kobayashi, Lu, Xu, Wu, Chang, Zhang and Jia, 2013. An unusual basal therizinosaur dinosaur with an ornithischian dental arrangement from northeastern China. PLoS ONE. 8(5), e63423. DOI: 10.1371/journal.pone.0063423**

Rauhut and Foth, 2014. New information on the theropod dinosaurs from the Late Jurassic lithographic limestones of southern Germany. Journal of Vertebrate Paleontology. Program and Abstracts 2014, 212.

Rauhut, Milner and Moore-Fay, 2010. Cranial osteology and phylogenetic position of the theropod dinosaur *Proceratosaurus bradleyi* (Woodward, 1910) from the Middle Jurassic of England. Zoological Journal of the Linnean Society. 158(1), 155-195. DOI: 10.1111/j.1096-3642.2009.00591.x

Russell, 1969. A new specimen of *Stenonychosaurus* from the Oldman Formation of Alberta. Canadian Journal of Earth Sciences. 6, 595-612. DOI: 10.1139/e69-059

Sanz, Chiappe, Perez-Moreno, Moratalla, Hernandez-Carrasquilla, Buscalioni, Ortega, Poyato-Ariza, Rasskin-Gutman and Martinez-Delclos, 1997. A nestling bird from the Lower Cretaceous of Spain: Implications for avian skull and neck evolution. Science. 276, 1543-1546. DOI: 10.1126/science.276.5318.1543

Senter, 2006a. Forelimb function in *Ornitholestes hermanni* Osborn (Dinosauria, Theropoda). Palaeontology. 49(5), 1029-1034. DOI: 10.1111/j.1475-4983.2006.00585.x

Senter, 2006b. Comparison of forelimb function between *Deinonychus* and *Bambiraptor* (Theropoda: Dromaeosauridae). Journal of Vertebrate Paleontology. 26(4), 897-906. DOI: 10.1671/0272-4634(2006)26[897:COFFBD]2.0.CO;2

**Senter, 2007. A new look at the phylogeny of Coelurosauria. Journal of Systematic Palaeontology. 5(4), 429-463. DOI: 10.1017/S1477201907002143**

Senter, 2009. Pedal function in deinonychosaurs (Dinosauria: Theropoda): A comparative study. Bulletin of Gunma Natural History Museum. 13, 1-14.

**Senter, 2010. Using creation science to demonstrate evolution: Application of a creationist method for visualizing gaps in the fossil record to a phylogenetic study of coelurosaurian dinosaurs. Journal of Evolutionary Biology. 23(8), 1732-1743. DOI: 10.1111/j.1420-9101.2010.02039.x**

Senter, 2011. Using creation science to demonstrate evolution 2: Morphological continuity within Dinosauria. Journal of Evolutionary Biology. 24(10), 2197-2216. DOI: 10.1111/j.1420-9101.2011.02349.x

Senter, Kirkland and DeBlieux, 2012b. *Martharaptor greenriverensis*, a new theropod dinosaur from the Lower Cretaceous of Utah. PLoS ONE. 7(8), e43911. DOI: 10.1371/journal.pone.0043911

Senter, Barsbold, Britt and Burnham, 2004. Systematics and evolution of Dromaeosauridae. Bulletin of Gunma Natural History Museum. 8, 1-20.

**Senter, Kirkland, Bird and Bartlett, 2010. A new troodontid theropod dinosaur from the Lower Cretaceous of Utah. PLoS ONE. 5(12), e14329. DOI: 10.1371/journal.pone.0014329**

Senter, Kirkland, DeBlieux, Madsen and Toth, 2012a. New dromaeosaurids (Dinosauria: Theropoda) from the Lower Cretaceous of Utah, and the evolution of the dromaeosaurid tail. PLoS ONE. 7(5), e36790. DOI: 10.1371/journal.pone.0036790

Sereno, 1998. A rationale for phylogenetic definitions, with application to the higher level taxonomy of Dinosauria. Neues Jahrbuch für Geologie und Paläontologie, Abhandlungen. 210(1), 41-83. DOI: 10.1127/njgpa/210/1998/41

Sereno, 1999. The evolution of dinosaurs. Science. 284, 2137-2147. DOI: 10.1126/science.284.5423.2137

Sereno and Brusatte, 2008. Basal abelisaurid and carcharodontosaurid theropods from the Lower Cretaceous Elrhaz Formation of Niger. Acta Palaeontologica Polonica. 53(1), 15-46.

**Serrano-Branas, Torres-Rodriguez, Reyes-Luna and Conzalez-Ramirez and Gonzalez-Leon, 2015. A new ornithomimid dinosaur from the Upper Cretaceous Packard Shale Formation (Cabullona Group) Sonora, Mexico. Cretaceous Research. 58, 49-62. DOI: 10.1016/j.cretres.2015.08.013**

**Shen, Lu, Liu, Kundrat, Brusatte and Gao, 2017. A new troodontid dinosaur from the Lower Cretaceous Yixian Formation of Liaoning Province, China. Acta Geologica Sinica. 91(3), 763-780. DOI: 10.1111/1755-6724.13307**

Smith and Galton, 1990. Osteology of *Archaeornithomimus asiaticus* (Upper Cretaceous, Iren Dabasu Formation, People's Republic of China). Journal of Vertebrate Paleontology. 10(2), 255-265. DOI: 10.1080/02724634.1990.10011811

**Sues and Averianov, 2014. Dromaeosauridae (Dinosauria: Theropoda) from the Bissekty Formation (Upper Cretaceous: Turonian) of Uzbekistan and the phylogenetic position of *Itemirus medullaris* Kurzanov, 1976. Cretaceous Research. 51, 225-240. DOI: 10.1016/j.cretres.2014.06.007**

Sues and Averianov, 2015a. New material of *Caenagnathasia martinsoni* (Dinosauria: Theropoda: Oviraptorosauria) from the Bissekty Formation (Upper Cretaceous: Turonian) of Uzbekistan. Cretaceous Research. 54, 50-59. DOI: 10.1016/j.cretres.2014.12.001

Sues and Averianov, 2015b. Ornithomimidae (Dinosauria: Theropoda) from the Bissekty Formation (Upper Cretaceous: Turonian) of Uzbekistan. Cretaceous Research. 57, 90-110. DOI: 10.1016/j.cretres.2015.07.012

**Sues and Averianov, 2015c. Therizinosauroidea (Dinosauria: Theropoda) from the Upper Cretaceous of Uzbekistan. Cretaceous Research. 59, 155-178. DOI: 10.1016/j.cretres.2015.11.003**

Suzuki, Chiappe, Dyke, Watabe, Barsbold and Tsogtbaatar, 2002. A new specimen of *Shuvuuia deserti* Chiappe et al., 1998, from the Mongolian Late Cretaceous with a discussion of the relationships of alvarezsaurids to other theropod dinosaurs. Contributions in Science (Los Angeles). 494, 1-18.

**Tsuihiji, Barsbold, Watabe, Tsogtbaatar, Suzuki and Hattori, 2015. New material of a troodontid theropod (Dinosauria: Saurischia) from the Lower Cretaceous of Mongolia. Historical Biology. 28(1-2), 128-138. DOI: 10.1080/08912963.2015.1005086**

**Tsuihiji, Barsbold, Watabe, Tsogtbaatar, Chinzorig, Fugiyama and Suzuki, 2014. An exquisitely preserved troodontid theropod with new information on the palatal structure from the Upper Cretaceous of Mongolia. Naturwissenschaften. 101, 131-142. DOI: 10.1007/s00114-014-1143-9**

**Turner, 2008. Phylogenetic relationships of paravian theropods. PhD Thesis, Columbia University. 666 pp.**

**Turner, Hwang and Norell, 2007a. A small derived theropod from Oosh, Early Cretaceous, Baykhangor Mongolia. American Museum Novitates. 3557, 27 pp.**

**Turner, Makovicky and Norell, 2012. A review of dromaeosaurid systematics and paravian phylogeny. Bulletin of the American Museum of Natural History. 371, 1-206.**

**Turner, Pol, Clarke, Ericson and Norell, 2007b. A basal dromaeosaurid and size evolution preceding avian flight. Science. 317, 1378-1381. DOI: 10.1126/science.1144066**

van der Reest and Currie, 2017. Troodontids (Theropoda) from the Dinosaur Park Formation, Alberta, with a description of a unique new taxon: Implications for deinonychosaur diversity in North America. Canadian Journal of Earth Sciences. 54(9), 919-935. DOI: 10.1139/cjes-2017-0031

Vasquez, 1993. Functional morphology of the avian wing and its implications for *Archaeopteryx* and the evolution of birds. PhD thesis, Yale University. 146 pp.

**Wahl, 2006. Osteology and phylogenetic relationships of a new small maniraptoran from the Upper Jurassic of Wyoming. Master's thesis, Fort Hays State University. 97 pp.**

Wang, Zhang, Sullivan and Xu, 2016. Elongatoolithid eggs containing oviraptorid (Theropoda, Oviraptorosauria) embryos from the Upper Cretaceous of southern China. BMC Evolutionary Biology. 16:67. DOI: 10.1186/s12862-016-0633-0

Wang, O'Connor, Zheng, Wang, Hu and Zhou, 2014. Insights into the evolution of rachis dominated tail feathers from a new basal enantiornithine (Aves: Ornithothoraces). Biological Journal of the Linnean Society. 113, 805-819. DOI: 10.1111/bij.12313

Wellnhofer, 1974. Das fünfte Skelettexemplar von *Archaeopteryx*. Palaeontographica. 147, 169-216.

Wellnhofer and Röper, 2005. Das neunte *Archaeopteryx*- Exemplar von Solnhofen. Archaeopteryx. 23, 3-21.

**Xu, 2002. Deinonychosaurian fossils from the Jehol Group of western Liaoning and the coelurosaurian evolution. PhD Thesis. Chinese Academy of Sciences. 325 pp.**

**Xu and Norell, 2004. A new troodontid dinosaur from China with avian-like sleeping posture. Nature. 431, 838-841. DOI: 10.1038/nature02898**

Xu and Wang, 2004a. A new troodontid (Theropoda: Troodontidae) from the Lower Cretaceous Yixian Formation of Western Liaoning, China. Acta Geologica Sinica. 78(1), 22-26. DOI: 10.1111/j.1755-6724.2004.tb00671.x

**Xu and Wang, 2004b. A new dromaeosaur (Dinosauria: Theropoda) from the Early Cretaceous Yixian Formation of Western Liaoning. Vertebrata PalAsiatica. 42(2), 111-119.**

**Xu and Zhang, 2005. A new maniraptoran dinosaur from China with long feathers on the metatarsus. Naturwissenschaften. 92, 173-177. 10.1007/s00114-004-0604-y**

Xu, Han and Zhao, 2014. Homologies and homeotic transformation of the theropod 'semilunate' carpal. Nature Scientific Reports. 4, 6042. DOI: 10.1038/srep06042

**Xu, Sullivan and Wang, 2013. The systematic position of the enigmatic theropod dinosaur *Yixianosaurus longimanus*. Vertebrata PalAsiatica. 51(3), 169-183.**

Xu, Zheng and You, 2009. A new feather type in a nonavian theropod and the early evolution of feathers. Proceedings of the National Academy of Sciences. 106(3), 832-834. DOI: 10.1073/pnas.0810055106

Xu, Zhou and Wang, 2000. The smallest known non-avian theropod dinosaur. Nature, 408, 705-708. DOI: 10.1038/35047056

**Xu, Cheng, Wang and Chang, 2002c. An unusual oviraptorosaurian dinosaur from China. Nature. 419, 291-293. DOI: 10.1038/nature00966**

**Xu, You, Du and Han, 2011. An *Archaeopteryx*-like theropod from China and the origin of Avialae. Nature. 475, 465-470. DOI: 10.1038/nature10288**

**Xu, Norell, Wang, Makovicky and Wu, 2002a. A basal troodontid from the Early Cretaceous of China. Nature. 415, 780-784. DOI: 10.1038/415780a**

**Xu, Tan, Sullivan, Han and Xiao, 2011. A short-armed troodontid dinosaur from the Upper Cretaceous of Inner Mongolia and its implications for troodontid evolution. PLoS ONE. 6(9), e22916. DOI: 10.1371/journal.pone.0022916**

**Xu, Tan, Wang, Zhao and Tan, 2007. A gigantic bird-like dinosaur from the Late Cretaceous of China. Nature. 447, 844-847. DOI: 10.1038/nature05849**

Xu, Zhou, Sullivan, Wang and Ren, 2016. An updated review of the Middle-Late Jurassic Yanliao Biota: Chronology, taphonomy, paleontology and paleoecology. Acta Geologica Sinica. 90(6), 2229-2243.

**Xu, Zhao, Sullivan, Tan, Sander and Ma, 2012. The taxonomy of the troodontid IVPP V 10597 reconsidered. Vertebrata PalAsiatica. 50(2), 140-150.**

Xu, Wang, Sullivan, Hone, Han, Yan and Du, 2010. A basal parvicursorine (Theropoda: Alvarezsauridae) from the Upper Cretaceous of China. Zootaxa. 2413, 1-19.

Xu, Zhang, Sereno, Zhao, Kuang, Han and Tan, 2002b. A new therizinosauroid (Dinosauria, Theropoda) from the Upper Cretaceous Iren Dabasu Formation of Nei Mongol. Vertebrata Palasiatica. 40(3), 228-240.

**Xu, Currie, Pittman, Xing, Meng, Lu, Hu and Yu, 2017. Mosaic evolution in an asymmetrically feathered troodontid dinosaur with transitional features. Nature Communications. 8:14972. DOI: 10.1038/ncomms14972**

**Xu, Zhao, Norell, Sullivan, Hone, Erickson, Wang, Han and Guo, 2009. A new feathered maniraptoran dinosaur fossil that fills a morphological gap in avian origin. Chinese Science Bulletin. 54(3), 430-435. DOI: 10.1007/s11434-009-0009-6**

**Xu, Zheng, Sullivan, Wang, Xing, Wang, Zhang, O'Connor, Zhang and Pan, 2015. A bizarre Jurassic maniraptoran theropod with preserved evidence of membranous wings. Nature. 521, 70-73. DOI: 10.1038/nature14423**

You, O'Connor, Chiappe and Ji, 2005. A new fossil bird from the Early Cretaceous of Gansu Province, northwestern China. Historical Biology. 17, 7-14. DOI: 10.1080/08912960500284851

Yu, Wang, Chen, Sullivan, Wang, Wang and Xu, 2018. A new caenagnathid dinosaur from the Upper Cretaceous Wangshi Group of Shandong, China, with comments on size variation among oviraptorosaurs. Scientific Reports. 8:5030. DOI:10.1038/s41598-018-23252-2

Zanno, 2006. The pectoral girdle and forelimb of the primitive therizinosauroid *Falcarius utahensis* (Theropoda, Maniraptora): Analyzing evolutionary trends within Therizinosauroidea. Journal of Vertebrate Paleontology. 26(3), 636-650. DOI: 10.1671/0272-4634(2006)26[636:TPGAFO]2.0.CO;2

Zanno, 2010a. Osteology of *Falcarius utahensis*: Characterizing the anatomy of basal therizinosaurs. Zoological Journal of the Linnaean Society. 158, 196-230. DOI: 10.1371/journal.pone.0198155

**Zanno, 2010b. A taxonomic and phylogenetic re-evaluation of Therizinosauria (Dinosauria: Maniraptora). Journal of Systematic Palaeontology. 8(4), 503-543. DOI: 10.1080/14772019.2010.488045**

**Zanno and Makovicky, 2011. On the earliest record of Cretaceous tyrannosauroids in western North America: Implications for an Early Cretaceous Laurasian interchange event. Historical Biology. 23(4), 317-325. DOI: 10.1080/08912963.2010.543952**

**Zanno, Gillette, Albright and Titus, 2009. A new North American therizinosaurid and the role of herbivory in 'predatory' dinosaur evolution. Proceedings of the Royal Society B. 76(1672), 3505-3511. DOI: 10.1098/rspb.2009.1029**

Zhang , Zhou, Xu and Wang, 2002. A juvenile coelurosaurian theropod from China indicates arboreal habits. Naturwissenschaften. 89, 394-398. DOI: 10.1007/s00114-002-0353-8

Zhang, Xu, Sereno, Kwang and Tan, 2001. A long-necked therizinosauroid dinosaur from the Upper Cretaceous Iren Dabasu Formation of Nei Mongol, People's Republic of China. Vertebrata PalAsiatica. 39(4), 282-290.

**Zhang, Zhou, Xu, Wang and Sullivan, 2008. A bizarre Jurassic maniraptoran from China with elongate ribbon-like feathers. Nature. 455, 1105-1108. DOI: 10.1038/nature07447**

**Zheng, Xu, You, Zhao and Dong, 2010. A short-armed dromaeosaurid from the Jehol Group of China with implications for early dromaeosaurid evolution. Proceedings of the Royal Society B. 277, 211-217. DOI: 10.1098/rspb.2009.1178**

Zhou, 1999. Early evolution of birds and avian flight-evidence from Mesozoic fossils and modern birds. PhD thesis, University of Kansas. 216 pp.

Zhou, Wang, Zhang and Xu, 2000. Important features of *Caudipteryx* - evidence from two nearly complete new specimens. Vertebrata PalAsiatica. 38(4), 241-254.

**Addendum: Revisiting *Sciurumimus***

*Sciurumimus* is unique among analyzed taxa in being fairly complete and emerging as a maniraptoromorph when the consensus places it outside Coelurosauria. Known from a complete articulated juvenile specimen, Rauhut et al. (2012) described the taxon as a megalosauroid based on the results of three phylogenetic analyses where it emerged outside Avetheropoda. Oddly, when compsognathid-grade *Juravenator* was added to two of these analyses it joined *Sciurumimus* to also emerge outside Avetheropoda, yet it was a coelurosaur if analyzed without *Sciurumimus*. Our analysis recovers *Sciurumimus* as a compsognathid closest to *Aorun*, former *Sinosauropteryx* specimen NGMC 2124 and *Compsognathus*. *Juravenator* emerges one node stemward sister to *Huaxiagnathus*. In this section, we aim to resolve these discrepencies.

Rauhut et al. (2012: 11750) list several "synapomorphies of megalosauroids and more restricted ingroups present in *Sciurumimus*" taken from their analysis adding it to Benson et al.'s (2010) matrix focusing on basal tetanurines. Rauhut et al. found adding *Sciurumimus* to Benson et al.'s analysis resulted in it being the first branching megalosaurid, and interestingly led to megalosauroids being carnosaurs instead of outside Avetheropoda.

"Elongate anterior process of the maxillary body" (character 9:1 in Benson et al.) is equivalent to our character 311, although our quantification method did not result in *Sciurumimus* being scored as having a long process. We do score the compsognathid *Aorun* as having a long processes however, not included by Benson et al.. Among their three (non-megaraptoran) coelurosaur OTUs, they did score *Guanlong* this way, but should have scored *Compsognathus** as elongate instead of short as well (Peyer, 2006: Fig. 4A).

"A medially closed maxillary fenestra" is our character 518:0 (15:1 in Benson et al.), and is also present in *Ornitholestes* (Witmer, 1997:42) and *Sinocalliopteryx* (Xing et al., 2012: Fig. 2A; misidentified as right maxilla) among basal maniraptoromorphs, neither included by Benson et al.. Note that analysis treats a medially closed fenestra, medially open fenestra and no fenestra as states of an unordered character, when the former two conditions both have the lateral opening in common and so should be considered closer to each other in an ordered character*. Thus megalosauroids have an intermediate condition of partial closure instead of an independant new state.

"A very slender anterior process of the lacrimal" (37:0 in Benson et al.) is a classic megalosauroid character not yet incorporated into TWiG analyses. Benson et al. only score *Eustreptospondylus* and *Torvosaurus* as having the condition, not *Afrovenator*, *Baryonyx* or *Irritator*. Given the varying lengths of the process and degree and duration of dorsoventral tapering, the state is not simple to quantify, and indeed Benson et al. merely define state 0 as "very slender, greatly reduced in height." Yet the condition in *Compsognathus** (Peyer, 2006: Fig. 4A) looks very similar to that in *Eustreptospondylus* (Sadleir et al., 2008: Plate 1 Fig. 6). A related issue is lateral exposure in articulated specimens, as *Irritator* (Sues et al., 2002: Fig. 1) and *Afrovenator* (Sereno et al., 1994: Fig. 2A) have narrowly exposed processes as seen in *Sciurumimus*, but may have been scored as plesiomorphic by Benson et al. based on the entire process if a dorsal portion was hidden. As the process is almost entirely covered dorsally by the articulated prefrontal in *Sciurumimus*, it's possible the entire process is deeper than the articulated specimen would suggest.

"A lateral blade of the lacrimal that does not overhang antorbital fenestra" (42:1 in Benson et al.) has not been added to TWiG analyses yet either. Among megalosauroids, Benson et al. score it as present in *Torvosaurus*, *Baryonyx* and *Irritator* but not *Eustreptospondylus*, *Afrovenator* or *Dubreuillosaurus*. It is actually overlapping in *Baryonyx** (Charig and Milner, 1997: Fig. 5A), as well as in the megalosauroid *Wiehenvenator* (Rauhut et al., 2016: Fig. 9.1). Among basal maniraptoromorphs, no overlap is present in *Ornitholestes* (AMNH 619: M.M. pers. obs.), *Scipionyx* (Dal Sasso and Maganuco, 2011: Fig. 23) and *Juravenator* (Chiappe and Gohlich, 2011: Fig. 8B), none of which were included by Benson et al..

"A deep fossa ventral to the basioccipital condyle" (67:1 in Benson et al.) is contrasted with a transversely narrow groove as in *Piatnitzkysaurus*. No coelurosaurs are scored for the character in Benson et al.'s matrix and it cannot be checked for most due to preservation, but the broad condition is present in *Fukuivenator* (Azuma et al., 2016: Fig. 4) and *Haplocheirus* (Choiniere et al., 2014: Fig. 7B) among basal maniraptoromorphs.

"A splenial foramen that opens anteroventrally" (97:1 in Benson et al.) is only scored as present in *Dubreuillosaurus* and *Monolophosaurus* among megalosauroids by Benson et al., with *Baryonyx* scored as having a closed foramen. An open foramen is also present in *Spinosaurus** (Stromer, 1915: Plate I Fig. 6) however. This state is also seen in *Aorun* (Choiniere et al., 2013:191), *Haplocheirus* (Choiniere et al., 2014:28) and *Compsognathus* (Peyer, 2006:885) among basal maniraptoromorphs, the latter included and scored correctly by Benson et al.. *Guanlong** was left unscored by Benson et al. but has the open condition (Choiniere, 2010:130).

"A slightly dorsally expanded anterior end of the dentary" (77:1 in Benson et al.) is subtle in *Sciurumimus*, with the greatest expansion just 5% taller than the dentary's narrowest point. This is typical of basal maniraptoromorphs, being seen in *Ornitholestes*, *Aorun* (Choiniere et al., 2013: Fig. 4C-D), *Scipionyx* (Dal Sasso and Maganuco, 2011: Fig. 44), *Juravenator* (Chiappe and Gohlich, 2011: Fig. 8B), *Sinosauropteryx* (Currie and Chen, 2001: Fig. 3a), *Compsognathus** (Gohlich et al., 2006: Plate 3 Fig. 2; misscored by Benson et al.), and especially expanded in *Huaxiagnathus* (Hwang et al., 2004: Fig. 2A) and *Haplocheirus* (Choiniere et al., 2014: Fig. 3).

"A pronounced ventral keel in the anterior dorsal vertebrae" (116:1 in Benson et al.) refers to the first and second dorsals, based on the condition in *Monolophosaurus*, *Piatnitzkysaurus* and *Baryonyx*. Whether *Sciurumimus* actually has this condition is not obvious from photographs, where centrum 1 is partially covered by its rib and the ventral portion of centrum 2 broken away. Among basal maniraptoromorphs it is difficult to determine in most slab specimens due to crushing and overlap, but this may be present in *Sinocalliopteryx* (Xing et al., 2012: Fig. 1) and is found in dorsal 2 of *Falcarius* (Zanno, 2010a: Fig. 4F, H) and an anterior dorsal of *Nqwebasaurus* (Choiniere et al., 2012: Fig. 10C, E). No coelurosaurs were scored by Benson et al., although the included *Guanlong** has pronounced keels on its first two dorsals (Choiniere, 2010:139-140, Fig. 3.21A, E-F, J)

"The absence of a posteroventral process of the coracoid" (140:0 in Benson et al.) was only scored in *Megalosaurus* and *Torvosaurus* by Benson et al., not *Piatnitzkysaurus* or baryonychines. However, *Megalosaurus** actually has a concavity posteroventral to and not including the glenoid (Benson, 2010: Fig. 11A, E, G) defining a posteroventral process. As no basal maniraptoromorphs exhibit the morphology, it is shared between *Torvosaurus* and *Sciurumimus* among that group and megalosauroids. The only other tetanurine with state 0 in Benson et al.'s matrix is *Sinraptor**, which was misscored and has a substantial subglenoid notch defining the posterlateral process (Gao, 1999; Fig. 23B, Plate 29 Fig. 2).

Finally, "an enlarged manual ungual I" (159:1 in Benson et al.) is referenced to Rauhut's (2003) character 161 which is discontinuously quantified as "less than half the length of the radius" (0) versus "more than two-thirds of the length of the radius" (1). It is scored as present in baryonychines and *Torvosaurus* and absent in *Afrovenator*. Yet the ungual provisionally referred to *Torvosaurus** (BYU 2020) was found 195 km from the type locality (Galton and Jensen, 1979:6), so its size compared to the type radius does not mean much. Based on the left elements in Rauhut et al.'s Figure 1c, the ratio in *Sciurumimus* is ~64%. This is met by basal maniraptoromorphs *Huaxiagnathus* (~73%- Hwang et al., 2004: Fig. 7A, B), *Sinosauropteryx* (~119%- Currie and Chen, 2001: Fig. 8a) and *Haplocheirus* (58-64%- Choiniere, 2010: Appendix 4), none of which were included by Benson et al.. The ratio in the included coelurosaur *Tanycolagreus** is basically the same as *Sciurumimus*, at 63% (Carpenter et al., 2005a: Fig. 2.3).

Most of the reported megalosauroid characters of *Sciurumimus* are also present in various basal maniraptoromorphs that were not included by Benson et al. with the exception of both it and *Torvosaurus* lacking a coracoid posteroventral process and possibly a very shallow anterior lacrimal process. If the twelve modifications asterisked above are made to Rauhut et al.'s matrix, *Sciurumimus* emerges as a coelurosaur in a trichotomy with *Compsognathus* and *Guanlong* plus *Tanycolagreus*. In these trees, megalosauroids, carnosaurs and coelurosaurs form an unresolved trichotomy.

When added to Choiniere et al.'s (2010) matrix, *Sciurumimus* emerged in a polytomy with *Afrovenator*, Megalosauria and *Monolophosaurus* plus avetheropods. Included taxa we recover as basal maniraptoromorphs are *Ornitholestes*, *Juravenator*, *Huaxiagnathus*, *Sinosauropteryx*, *Compsognathus* and *Haplocheirus*. Using one of the MPTs where *Sciurumimus* is a megalosauroid, examination of the characters which exclude it from more crownward groups shows that only two are different from all of the included compsognathids- "Pleurocoel in axis ... (0) absent" (181:0 in Choiniere et al.) and "Ventral notch at distal edge of ischial obturator process ... (1) present, makes obturator process triangular in lateral view" (355:1 in Choiniere et al.), although the unincluded *Scipionyx* also has an obturator notch. Both are included in our analysis (517:0 and 317:0 respectively).

Numerous misscorings by Choiniere et al. were identified when checking character support for a non-compsognathid *Sciurumimus*, but correcting the following handful led to trees where it is the first branching member of Compsognathidae within Coelurosauria-

*Sinosauropteryx* (Currie and Chen, 2001: Fig. 3a), *Compsognathus* (Peyer, 2006: Fig. 4A), *Juravenator* (Chiappe and Gohlich, 2011: Fig. 8) and *Huaxiagnathus* (Hwang et al., 2004: Fig. 2) were misscored by Choiniere et al. as either lacking nasal-antorbital fossa contact (first two) or having an unknown condition (last two), but all four have the contact (27 in Choiniere et al.).

Choiniere misscored *Juravenator* (Chiappe and Gohlich, 2011: Fig. 8), *Huaxiagnathus* (Hwang et al., 2004: Fig. 2) and *Compsognathus* (Peyer, 2006: Fig. 4; misscored as unknown) as not having a triangular ventrally expanded ventral lacrimal process (57 in Choiniere et al.).

*Garudimimus* was misscored as having axial pleurocoels (Kobayashi and Barsbold, 2005b:1512) (181 in Choiniere et al.).

Choiniere et al. misscored *Allosaurus* (Madsen, 1976: Plate 42D, F), *Albertosaurus* (Parks, 1928a: Fig. 3) and *Tyrannosaurus* (Brochu, 2003: Fig. 86A-C) as having a strongly reduced olecranon process (273 in Choiniere et al.).

*Baryonyx* was misscored by Choiniere et al. as having a ventral median groove on manual phalanx I-1, yet it does not preserve that phalanx (Charig and Milner, 1997:46-47) (299 in Choiniere et al.).

*Sinosauropteryx* was misscored by Choiniere et al. as not having a large manual ungual I with "unguals of other digits distinctly smaller" (Currie and Chen, 2001: Fig. 9) (306 in Choiniere et al.).

*Sciurumimus* has a pre/postacetabular ratio of 75% (Rauhut et al., 2012: Fig. 1d, 2c-d), so is comparable to *Huaxiagnathus* (78%- Hwang et al., 2004: Fig. 9) and *Juravenator* (70%- Chiappe and Gohlich, 2011: Fig. 13) and *Compsognathus* (71%- Peyer, 2006: Fig. 7) and was thus misscored by Rauhut et al. as having a short preacetabular process (315 in Choiniere et al.).

*Compsognathus* (distal tip 34% up metatarsal II- Ostrom, 1978: Fig. 1) and *Juravenator* (32-39%- Chiappe and Gohlich, 2011: Fig. 23) were misscored by Choiniere et al. as having metatarsal I attached to the distal quarter of metatarsal II (408 in Choiniere et al.).
[truncated: 94,233 more chars]
